# Supplementary material for: A phase 2 randomised controlled trial of mazdutide in Chinese overweight adults or adults with obesity
Source: Nat Commun. 2023 Dec 14;14:8289. doi: 10.1038/s41467-023-44067-4 (PMC10719339; doi:10.1038/s41467-023-44067-4)
Supplement: Supplementary file 1 — Supplementary Information [file 41467_2023_44067_MOESM1_ESM.pdf]

A phase 2 randomised controlled trial of mazdutide in Chinese overweight adults or adults with obesity

Supplementary Information

1. Supplementary Figures
2. Supplementary Tables
3. Supplementary Note 1: Clinical Study Protocol
4. Supplementary Note 2: Statistical Analysis Plan

Supplementary Figures

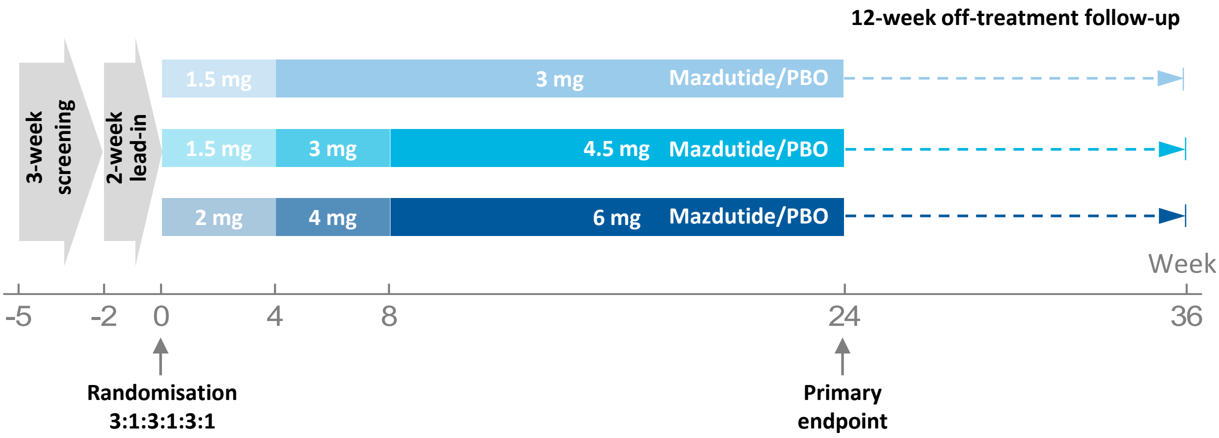

Figure S1: Study design

PBO = placebo

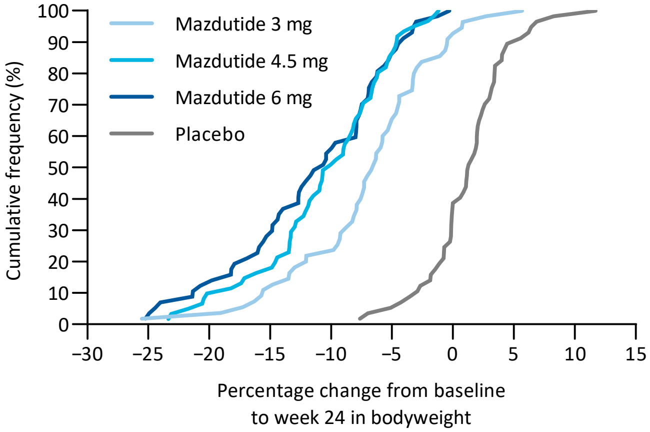

Figure S2: Cumulative distribution plot of percentage change from baseline in body weight at week 24.

Includes participants with body weight values at week 24. Mazdutide 3 mg *n* = 55; mazdutide 4.5 mg *n* = 61; mazdutide 6 mg *n* = 57; placebo *n* = 57.

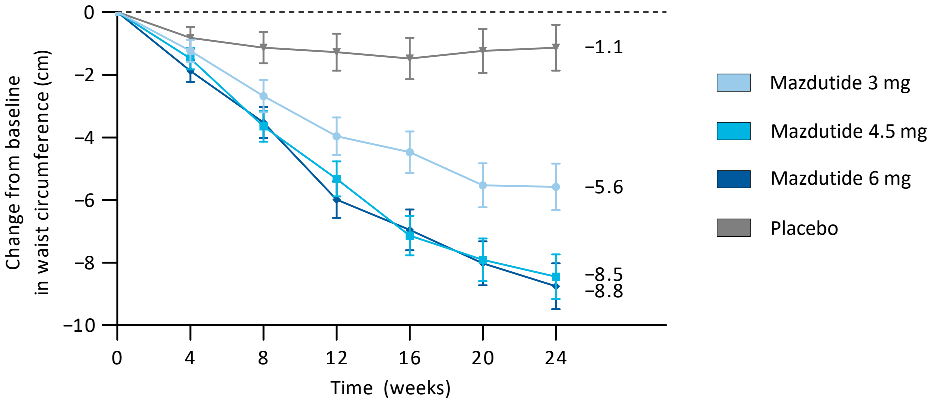

Figure S3: Change from baseline in waist circumference over time.

Data are LSM (SE) from MMRM analysis, mITT population. Mazdutide 3 mg *n* = 62; mazdutide 4.5 mg *n* = 63; mazdutide 6 mg *n* = 61; placebo *n* = 62. LSM=least squares mean. MMRM=mixed model repeated measures. SE = standard error. Source data are provided as a Source Data file.

a

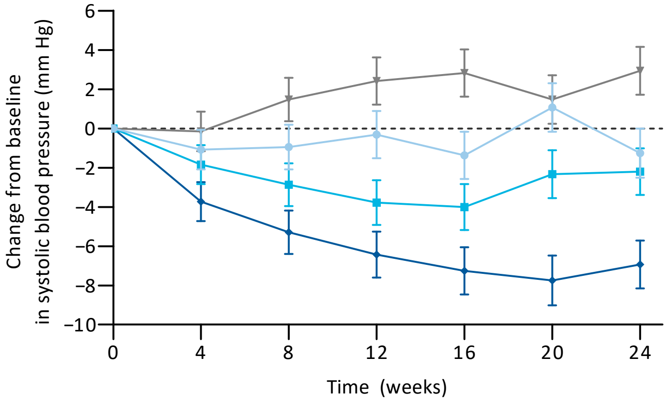

b

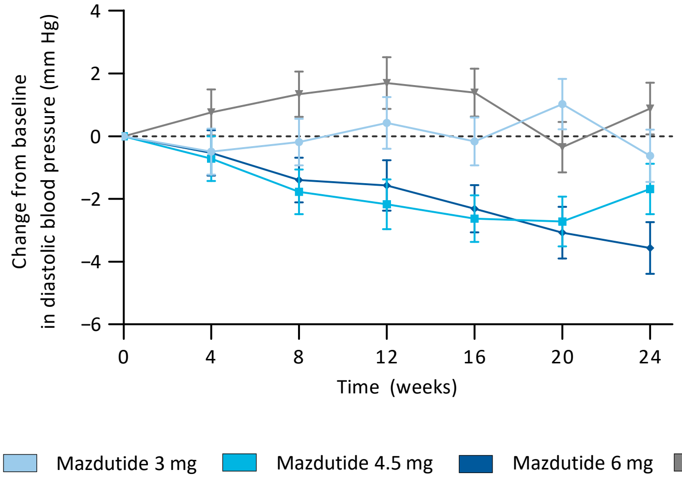

Figure S4: Change from baseline in systolic (A) and diastolic (B) blood pressure over time.

Data are LSM (SE) from MMRM analysis, mITT population. Mazdutide 3 mg *n* = 62; mazdutide 4.5 mg *n* = 63; mazdutide 6 mg *n* = 61; placebo *n* = 62. LSM=least squares mean. MMRM=mixed model repeated measures. SE = standard error. Source data are provided as a Source Data file.

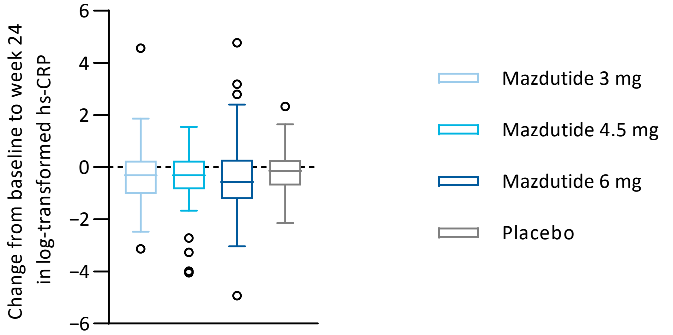

Figure S5: Change from baseline to week 24 in log-transformed hs-CRP.

Data are plotted as boxes and whiskers using Tukey method. Includes participants with hs-CRP measurement at week 24. Mazdutide 3 mg *n* = 55; mazdutide 4.5 mg *n* = 61; mazdutide 6 mg *n* = 57; placebo *n* = 56. hs-CRP=high-sensitivity C-reactive protein.

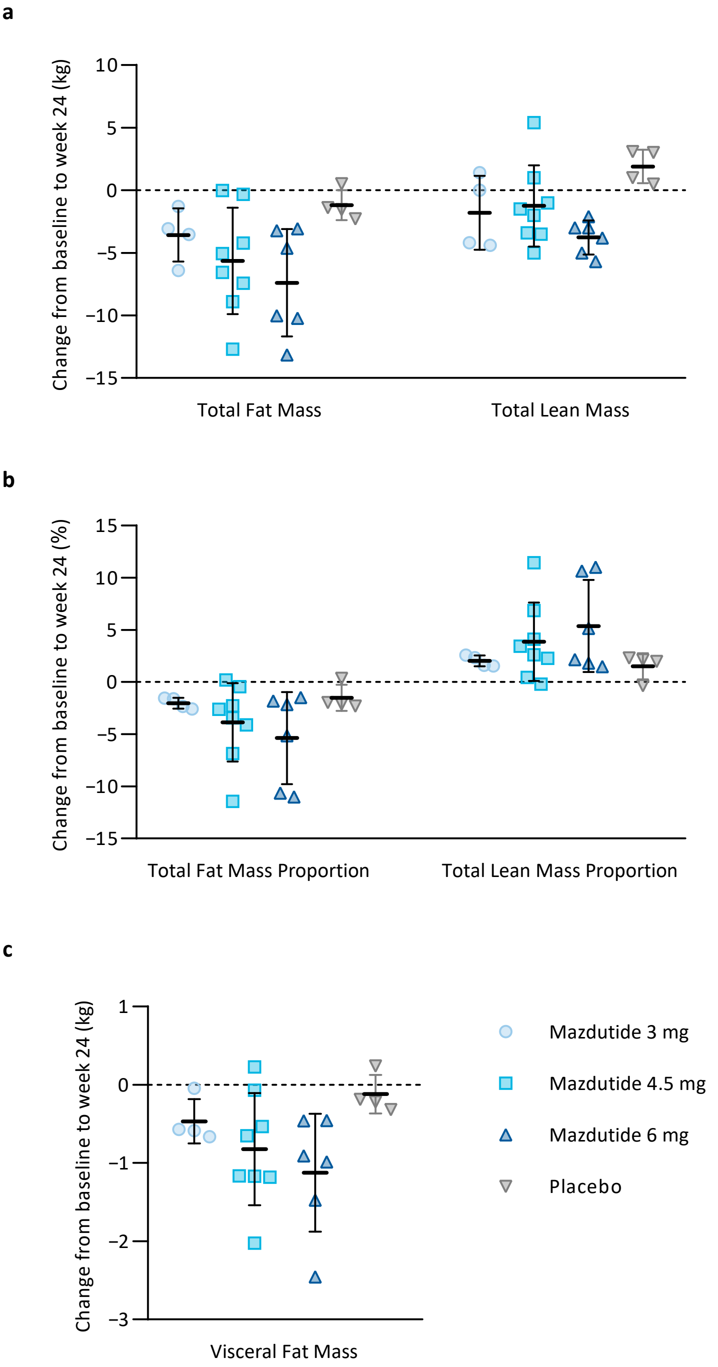

Figure S6: Change from baseline to week 24 in body composition by DEXA.

Data are individual values with error bars representing mean and SD. **A**. change from baseline to week 24 in total fat mass and total lean mass. **B**. change from baseline to week 24 in total fat mass proportion and total lean mass proportion, calculated by respective values divided total body mass. **C**. change from baseline to week 24 in visceral fat mass, calculated in the android region (male only) or gynoid region (female only). Mazdutide 3 mg *n* = 4; mazdutide 4.5 mg *n* = 8; mazdutide 6 mg *n* = 6; placebo *n* = 4. DEXA=dual energy X-ray absorptiometry. SD=standard deviation.

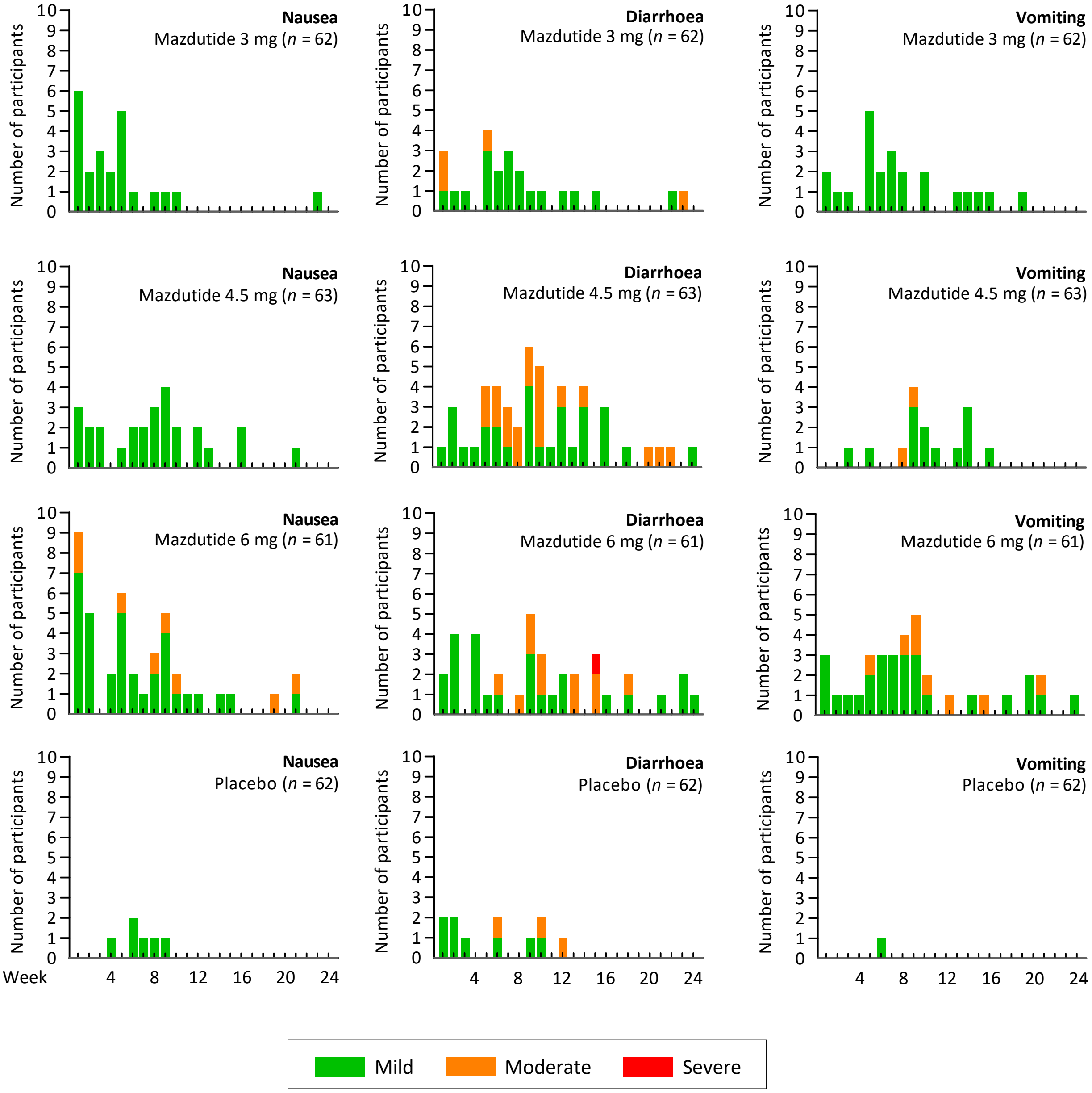

Figure S7: By-week incidence of diarrhoea, nausea and vomiting over time (safety population).

Mazdutide 3 mg *n* = 62; mazdutide 4.5 mg *n* = 63; mazdutide 6 mg *n* = 61; placebo *n* = 62.

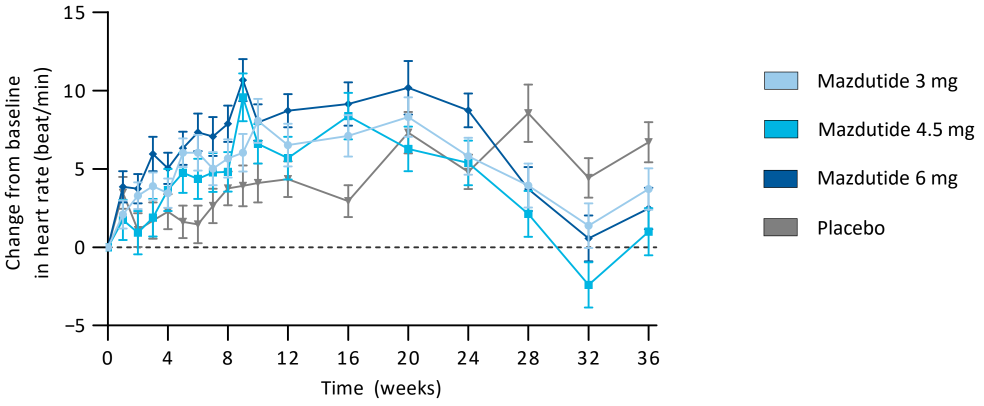

Figure S8: Change from baseline in heart rate over time.

Data are mean (SEM), safety population. Mazdutide 3 mg *n* = 62; mazdutide 4.5 mg *n* = 63; mazdutide 6 mg *n* = 61; placebo *n* = 62. SEM=standard error of the mean. Source data are provided as a Source Data file.

Supplementary Tables

Table S1: Percentage change from baseline in body weight at week 24 (sensitivity analyses)

	Mazdutide 3 mg (n = 62)		Mazdutide 4.5 mg (n = 63)		Mazdutide 6 mg (n = 61)		Placebo (n = 62)
	Mean	p	Mean	p	Mean	p	Mean
**Primary endpoint (sensitivity analysis – ANCOVA + MI)**							
Percentage change from baseline in body weight, %	−7.2 (0.8)		−10.5 (0.7)		−11.6 (0.7)		1.1 (0.8)
ETD versus placebo	−8.3 (−10.1, −6.4)	<0.0001	−11.6 (−13.4, −9.8)	<0.0001	−12.6 (−14.5, −10.8)	<0.0001	
**Primary endpoint (sensitivity analysis – MMRM)**							
Percentage change from baseline in body weight, %	−7.2 (0.7)		−10.6 (0.7)		−11.6 (0.7)		1.0 (0.7)
ETD versus placebo	−8.3 (−10.1, −6.4)	<0.0001	−11.6 (−13.4, −9.8)	<0.0001	−12.6 (−14.5, −10.8)	<0.0001	

Data are LSM (SE) for change and percentage change from baseline and LSM (95% CI) for ETD, from MMRM or ANCOVA analysis, mITT population. All statistical tests were two-sided at a significance level of 0.05, and no adjustments were made for multiplicity. P values were nominal. ANCOVA=analysis of covariate. ETD=estimated treatment difference. LSM=least squares mean. MI=multiple imputation. MMRM=mixed model repeated measures.

Table S2: Change from baseline in HOMA-IR at week 24

	Mazdutide 3 mg (n = 62)		Mazdutide 4.5 mg (n = 63)		Mazdutide 6 mg (n = 61)		Placebo (n = 62)
	Mean	p	Mean	p	Mean	p	Mean
**HOMA-IR**							
Baseline	3.4 (2.3–5.4)		3.5 (2.1–5.8)		3.2 (2.6–3.9)		3.7 (2.4–5.3)
Change from baseline	−1.6 (0.4)		−2.2 (0.4)		−2.1 (0.4)		−0.2 (0.4)
ETD versus placebo	−1.4 (−2.4, −0.3)	0.0094	−2.0 (−3.0, −1.0)	0.0001	−1.8 (−2.9, −0.8)	0.0004	

Data are median (interquartile range) for baseline, LSM (SE) for change from baseline and LSM (95% CI) for ETD at week 24 from MMRM analysis, mITT population. All statistical tests were two-sided at a significance level of 0.05, and no adjustments were made for multiplicity. P values were nominal. ETD=estimated treatment difference. LSM=least squares mean. MMRM=mixed model repeated measures.

Table S3: Efficacy endpoints at week 36

	Mazdutide 3 mg (n = 62)		Mazdutide 4.5 mg (n = 63)		Mazdutide 6 mg (n = 61)		Placebo (n = 62)
	Mean	p	Mean	p	Mean	p	Mean
**Body weight**							
Change from baseline, kg	−4.5 (0.6)		−6.1 (0.6)		−6.9 (0.6)		0.9 (0.6)
ETD versus placebo	−5.4 (−7.1, −3.7)	<0.0001	−7.0 (−8.7, −5.3)	<0.0001	−7.8 (−9.5, −6.1)	<0.0001	
Percentage change from baseline, %	−5.2 (0.7)		−7.2 (0.7)		−8.1 (0.7)		0.8 (0.7)
ETD versus placebo	−6.0 (−8.0, −4.0)	<0.0001	−8.0 (−10.0, −6.0)	<0.0001	−9.0 (−11.0, −7.0)	<0.0001	
**BMI**							
Change from baseline, kg/m^2	−1.7 (0.2)		−2.2 (0.2)		−2.6 (0.2)		0.3 (0.2)
ETD versus placebo	−2.0 (−2.6, −1.3)	<0.0001	−2.5 (−3.2, −1.9)	<0.0001	−2.9 (−3.5, −2.2)	<0.0001	
**Waist circumference**							
Change from baseline, cm	−4.6 (0.7)		−6.8 (0.7)		−6.7 (0.7)		−1.3 (0.7)
ETD versus placebo	−3.3 (−5.3, −1.3)	0.0011	−5.5 (−7.4, −3.5)	<0.0001	−5.4 (−7.4, −3.4)	<0.0001	
**Systolic blood pressure**							
Change from baseline, mm Hg	1.1 (1.2)		−0.5 (1.2)		−4.4 (1.2)		1.8 (1.2)
ETD versus placebo	−0.7 (−3.8, 2.5)	0.6713	−2.2 (−5.3, 0.9)	0.1627	−6.2 (−9.4, −3.0)	0.0002	
**Diastolic blood pressure**							
Change from baseline, mm Hg	−0.8 (0.8)		−2.4 (0.8)		−2.8 (0.8)		−0.1 (0.8)
ETD versus placebo	−0.7 (−2.8, 1.5)	0.5332	−2.3 (−4.4, −0.2)	0.0332	−2.7 (−4.8, −0.5)	0.0157	

Data are LSM (SE) for change and percentage change from baseline and LSM (95% CI) for ETD, from MMRM analysis, mITT population. All statistical tests were two-sided at a significance level of 0.05, and no adjustments were made for multiplicity. P values were nominal. BMI=body-mass index. ETD=estimated treatment difference. LSM=least squares mean. MMRM=mixed model repeated measures.

Table S4: Other safety endpoints (safety population)

	Mazdutide 3 mg (n = 62)	Mazdutide 4.5 mg (n = 63)	Mazdutide 6 mg (n = 61)	Placebo (n = 62)
Participants with ≥3 times upper limit of normal ALT	0	0	0	0
Participants with ≥3 times upper limit of normal AST	0	1 (1.6)	0	0
Participants with ≥3 times upper limit of normal lipase	1 (1.6)	0	1 (1.6)	0
Participants with ≥3 times upper limit of normal amylase	0	0	0	0
Post-baseline calcitonin ≥20 ng/L	0	0	0	0
**Participants with mazdutide ADA**				
At baseline	3 (4.8)	3 (4.8)	1 (1.6)	NA
Post baseline treatment-induced ADA	14 (22.6)	17 (27.0)	20 (32.8)	NA
Post baseline treatment-emergent ADA	1 (1.6)	2 (3.2)	0	NA

Data are n (%). All percentages are relative to the total number of participants in each treatment group in the safety population. A subject is considered to be treatment-induced ADA positive if the subject is ADA negative at baseline and has at least one post baseline positive measurement. A subject is considered to be treatment-emergent ADA positive if the subject is ADA positive at baseline and has at least one post baseline titre that is a 4-fold or greater increase in titre from baseline measurement. ADA=anti-drug antibody. ALT=alanine aminotransferase. AST=aspartate aminotransferase.

Supplementary Note 1: Clinical Study Protocol

Clinical Study Protocol

**Study Title:**	A Randomized, Double-Blind, Placebo-Controlled Phase 2 Study to Evaluate the Efficacy and Safety of IBI362 in Chinese Subjects with Overweight or Obesity
**Protocol Number:**	CIBI362B201
**Version date and version number:**	V5.0/24 Aug 2022
**Product Name:**	Glucagon-like peptide-1 receptor/glucagon receptor (GLP-1R/GCGR) dual agonist injection (R&D code: IBI362)
**Study Phase:**	Phase 2
**Sponsor:**	Innovent Biologics (Suzhou) Co., Ltd.<br>No. 168 Dongping Street, Suzhou Industrial Park, Jiangsu Province, China
**Sponsor Contact:**	Deng Huan, Senior Director of Clinical Medicine<br>Tel: +86-13501731859<br>huan.deng@innoventbio.com

Confidentiality Statement

This document contains confidential information of Innovent Biologics (Suzhou) Co., Ltd.

The contents of this document should not be disclosed to anyone other than the investigator, study consultant or related personnel, institutional review board/independent ethics committee, or any institution.

The information in this document may not be used for any purpose other than the evaluation or conduct of this clinical study without written permission from the Sponsor.

Sponsor Signature Page

Study Title: A Randomized, Double-Blind, Placebo-Controlled Phase 2 Study to Evaluate the Efficacy and Safety of IBI362 in Chinese Subjects with Overweight or Obesity

Protocol No.: CIBI362B201

Title	Name	Signature (Print)	Date
Senior Director, Clinical Medicine	Deng Huan	____________________	______________________
Associate Director, Biostatistics	Li Li	____________________	______________________

Investigator Signature Page

Study Title: A Randomized, Double-Blind, Placebo-Controlled Phase 2 Study to Evaluate the Efficacy and Safety of IBI362 in Chinese Subjects with Overweight or Obesity

Protocol No.: CIBI362B201

This protocol is a trade secret of Innovent Biologics (Suzhou) Co., Ltd. I have read and fully understand this protocol and undertake to conduct this study in accordance with this protocol and the requirements of Good Clinical Practice and in compliance with applicable laws and regulations and the Declaration of Helsinki. At the same time, I undertake not to disclose any confidential information in this study to any third party without the written consent of Innovent Biologics (Suzhou) Co., Ltd.

Instructions for Investigators:<br>Please sign and date this signature page, print the investigator's name, professional title and the name of the study site, and return it to Innovent Biologics (Suzhou) Co., Ltd. After signing.

I have read the entire contents of this protocol and warrant that this study will be conducted as required:

Signature of Investigator: ____________________ Date _________________

Printed Name: ____________________

Investigator Title: ____________________

Tel.: ____________________

Site Name/Address: ____

____

____

Protocol Synopsis

**Protocol Number**	CIBI362B201
**Sponsor**	Innovent Biologics (Suzhou) Co., Ltd.
**Study drug**	IBI362 Injection
**Active ingredient**	GLP-1R (glucagon-like peptide-1 receptor)/GCGR (glucagon receptor) dual agonist
**Study Title**	A Randomized, Double-Blind, Placebo-Controlled Phase 2 Study to Evaluate the Efficacy and Safety of IBI362 in Chinese Subjects with Overweight or Obesity
**Study Phase**	Phase 2
**Study Objectives:**	• **Primary objective:**<br>To evaluate the change from baseline in body weight after administration of IBI362 for 24 weeks, and to recommend the appropriate dose for Phase 3 clinical trial.<br>• **Secondary objectives:**<br>1. To evaluate the safety of IBI362 administered for 24 weeks;<br>2. To evaluate the changes from baseline in indicators related to comorbidities after administration of IBI362 for 24 weeks;<br>3. To evaluate the rebound of body weight from baseline after administration of IBI362 for 24 weeks and 12 weeks after discontinuation;<br>4. To assess the population pharmacokinetic profile and pharmacodynamic profile of IBI362 in subjects with overweight and obesity.<br>• **Exploratory Objectives:**<br>1. To explore the effect of IBI362 on the related indicators of metabolic-related fatty liver disease;<br>2. To explore the effect of IBI362 on body fat.
**Study Endpoints:**	**Primary endpoint:**<br>• Percent change (%) of body weight from baseline after 24 weeks of administration.<br>**Secondary endpoints:**<br>（1） **Safety Endpoints**<br>• To evaluate the safety of IBI362 in subjects with different doses (adverse events, hypoglycemic events, vital signs, physical examination, laboratory tests, 12-lead ECG, etc.);<br>• Evaluate the mental health status of subjects after administration (C-SSRS questionnaire, PHQ-9 questionnaire);<br>• Incidence of anti-drug antibody (ADA) and neutralizing antibody (NAb) against IBI362 in serum before and after administration.

	（2） **Efficacy Endpoints**<br>• The proportion of subjects with body weight loss ≥ 5. 0% from baseline after administration for 24 weeks;<br>• The proportion of subjects with body weight loss ≥ 10. 0% from baseline after administration for 24 weeks;<br>• Absolute change from baseline in body weight (Kg) after administration for 24 weeks;<br>• Changes from baseline in waist circumference, BMI, glycated hemoglobin A1c (HbA1c), fasting plasma glucose, systolic blood pressure, diastolic blood pressure, blood lipids (TC, TG, LDL-C, HDL-C) after administration for 24 weeks;<br>• Percentage change (%) of body weight from baseline after 24-week administration and 12-week withdrawal;<br>• Absolute change in body weight (Kg) from baseline after 12 weeks of withdrawal after 24 weeks of administration;<br>• The proportion of subjects with body weight loss ≥ 5. 0% and 10.0% from baseline after treatment for 24 weeks and 12 weeks of discontinuation;<br>• Changes from baseline in waist circumference, BMI, HbA1c, fasting plasma glucose, systolic blood pressure, diastolic blood pressure and blood lipids (TC, TG, LDL-C and HDL-C) after 12-week discontinuation after administration for 24 weeks;<br>• To evaluate the changes from baseline in serum uric acid and alanine aminotransferase levels after 12 and 24 weeks of administration;<br>• To evaluate the improvement of quality of life (IWQoL-Lite questionnaire) after 24 weeks of administration;<br>（3） **Pharmacokinetic and Pharmacodynamic Characteristics**<br>• To assess the population pharmacokinetic and pharmacodynamic profile of IBI362 in subjects with overweight or obesity:<br>➢ The population pharmacokinetic profile of IBI362 will be analyzed using nonlinear mixed effects modeling;<br>➢ Pharmacodynamic parameters, including changes in fasting plasma glucose and fasting insulin at different time points before and after administration.<br>**Exploratory Endpoints:**<br>• To evaluate the changes of high-sensitivity C-Reactive Protein (hsCRP), Proproteinconvertase Subtilisin/Kexin Type 9 (PCSK9), Fibroblast Growth Factor-21 (FGF-21) and adiponectin levels from baseline after administration for 24 weeks;

	• The total fat mass, regional visceral fat mass and total lean body mass measured by Dual Energy X-ray Absorptiometry (DEXA) were evaluated, and the changes of each index from baseline at week 24 of dosing;<br>• Evaluate the intra-abdominal fat area (VFA), subcutaneous fat area (SFA) and total intra-abdominal fat area (TFA) measured by Magnetic Resonance Imaging (MRI), and the changes of each index from baseline after administration for 24 weeks;<br>• To assess the liver fat content as measured by Magnetic Resonance Imaging Derived Proton Density Fat Fraction (MRI-PDFF), and compare the change from baseline after 24 weeks of dosing.
**Study Design**	This was a multicenter, double-blind, randomized, placebo-controlled study in subjects with Overweight or Obesity. In the first and second stages of this study, subjects will be enrolled and analyzed separately, with no influence on each other.<br>Approximately 240 subjects will be enrolled in the first stage of the study, and eligible subjects will receive a 2-week placebo run-in, then will be randomized to 3.0 mg, 4.5 mg, and 6.0 mg dose groups and will be randomized in a 3: 1 ratio to receive IBI362 and placebo within each group; Randomization was stratified by BMI < 28.0 kg/m^2 and BMI ≥ 28. 0 kg/m^2 at randomization. Subjects will receive weekly dosing for a total of 24 weeks of double-blind treatment. The dose regimens are as follows: 3.0 mg dose group will be titrated from 1.5 mg, 4 weeks later (4 doses) to 3.0 mg, and the treatment will be maintained for 20 weeks; The 4.5 mg dose group should be titrated from 1.5 mg to 3.0 mg after 4 weeks (4 doses), 3.0 mg to 4.5 mg after 4 weeks, and maintained for 16 weeks; The 6.0 mg dose group should be titrated from 2.0 mg to 4.0 mg after 4 weeks (4 doses), 4.0 mg to 6.0 mg after 4 weeks, and maintained for 16 weeks.<br>Approximately 80 subjects are planned to be enrolled in the second stage of the study, and eligible subjects will receive a placebo run-in for 2 weeks and then be randomized at a ratio of IBI362: placebo = 3: 1 after successful run-in. Subjects were dosed weekly, starting at 3.0 mg and titrated to 6.0 mg after 4 weeks (4 doses) and 6.0 mg to 9.0 mg after 4 weeks, and maintained for 16 weeks.<br>The entire trial period consisted of a 3-week screening period, a 2-week placebo run-in period, a 24-week double-blind treatment period, and a 12-week off-treatment follow-up period. Subjects were required to maintain diet and exercise control throughout the study.<br>**Dose Modification Criteria:**

	1. If the subject in the 4.5 mg dose group cannot tolerate the dose after reaching the specified target titration dose (4.5 mg) and the investigator considers that the patient cannot tolerate the dose for further exposure, after discussion with the sponsor, it is recommended that the dose can be reduced to 3.0 mg 2 weeks after reaching the specified target titration dose (4.5 mg) and maintained at this dose until the end of the trial.<br>2. If the subject in the 6.0 mg dose group cannot tolerate the dose after reaching the specified target titration dose (6.0 mg), and the investigator considers that the patient cannot tolerate the dose for further exposure, after discussion with the sponsor, it is recommended that the dose can be reduced to 4.5 mg 2 weeks after reaching the specified target titration dose (6.0 mg) and maintained at this dose until the end of the trial.<br>3. If the subject in the 9.0 mg dose group cannot tolerate the dose of 6.0 mg after reaching the titration dose (e.g., there is moderate to severe vomiting or diarrhea, and the gastrointestinal adverse reactions have not been relieved after symptomatic treatment and/or 1-week interruption of the dose), and the investigator considers that the patient cannot tolerate the dose for further exposure, the dose can be reduced to 3.0 mg after the discussion with the sponsor, and the dose can be re-increased to 6.0 mg 2 weeks later according to the subject's condition. If the dose is still intolerable after increasing to 6.0 mg, the dose can be adjusted back to 3mg and maintained at this dose until the end of the study; If the 6.0 mg dose was tolerated, the dose was continued increased to 9.0 mg 4 weeks later according to the subject's condition.<br>4. If the subject in the 9.0 mg dose group can not tolerate the dose after reaching the specified target titration dose (9.0 mg) (e.g., there is moderate to severe vomiting or diarrhea, gastrointestinal adverse reactions remain unresolved after symptomatic treatment and/or 1-week interruption), and the investigator considers that the patient cannot tolerate the dose for further exposure, the dose can be reduced to 6.0 mg after the discussion with the sponsor and maintained at this dose until the end of the study or continued to be up-titrated to 9.0 mg after 2 weeks of 6.0 mg according to the subject's condition.
**Planned sample size**	Approximately 320
**Inclusion Criteria**	1. Age 18 ~ 75 years (both inclusive), male or female;<br>2. Stage 1: Obese: BMI ≥ 28. 0 kg/m^2; Or overweight: BMI 24.0 kg/m^2 < 28.0 kg/m^2 with at least one of the following comorbidites: i. Strong appetite, hunger before meal, more food intake per meal; ii. Comorbid with one or more of pre-diabetes (impaired fasting glucose and/or impaired glucose

	tolerance), hypertension, dyslipidemia (refer to Appendix 2 for details) and fatty liver (within 6 months prior to screening); iii. Combined weight-bearing joint pain; iv. Obesity-induced dyspnea or obstructive sleep apnea syndrome; Stage 2: Obese: BMI ≥ 30. 0 kg/m^2.<br>3. Body weight change before and after run-in period is less than 5.0%, calculation formula:<br>Percent change in body weight = (weight before randomization-weight on the day of run-in)/weight before randomization * 100%;<br>4. Able to understand the procedures and methods of this study, willing to strictly comply with the clinical trial protocol to complete this trial, and voluntarily sign the informed consent form.
**Exclusion Criteria**	1. The investigator suspects that the subject may be allergic to the study drug or components or drugs of the same class.<br>2. Weight change > 5.0% (chief complaint) controlled with diet and exercise alone for at least 12 weeks prior to screening.<br>3. Use of any of the following medications or treatments prior to screening:<br>1) Use of GLP-1 receptor (GLP-1R) agonists or GLP-1R/GCGR agonists or GIPR/GLP-1R agonists or GIPR/GLP-1R/GCGR agonists within 3 months prior to screening;<br>2) Subjects who have used drugs affecting body weight within 3 months prior to screening, including systemic steroids (intravenous, oral or intra-articular administration), tricyclic antidepressants, psychiatric drugs or sedative drugs (such as imipramine, amitriptyline, mirtazapine, paroxetine, phenelzine, chlorpromazine, thioridazine, clozapine, olanzapine, valproic acid, valproic acid derivatives, lithium salts);<br>3) Patients who have used Chinese herbal medicine, health products, meal replacements that affect body weight within 3 months before screening;<br>4) Have used or are currently using weight loss drugs within 3 months prior to screening, such as sibutramine hydrochloride, orlistat, phentermine, phenylpropanolamine, chlorpheniramine, phentermine, bupropion, lorcaserin, phentermine/topiramate mixture, naltrexone/bupropion mixture, etc.;<br>5) Subjects who have used glucose-lowering drugs, such as metformin, SGLT2 inhibitors, thiazolidinediones (TZDs), etc. within 3 months prior to screening;<br>6) Participation in other clinical trials (treated with an investigational drug) within 3 months prior to screening.<br>4. History or evidence of any of the following:

	1) HbA1c ≥ 6. 5% at screening or patients previously diagnosed with type 1 or type 2 diabetes;<br>2) Fasting venous blood glucose ≥ 7. 0 mmol/L at screening or venous blood glucose ≥ 11. 1 mmol/L 2 hours after 75 g Oral Glucose Toleracnce Test (OGTT) (for subjects with fasting blood glucose of 6.1-6.9 mmol/L at screening, venous blood glucose 2 hours after OGTT glucose load should be collected for confirmation);<br>3) Patients with retinopathy in the past or at screening;<br>4) Previously severe hypoglycemia or recurrent symptomatic hypoglycemia (≥ 2 times in half a year);<br>5) Obesity caused by secondary diseases or drugs, including increased cortisol hormone (such as Cushing's syndrome), obesity caused by pituitary gland and hypothalamus injury, obesity caused by reduction/withdrawal of weight-loss drugs, etc.;<br>6) Previous bariatric surgery (except acupuncture for weight loss, liposuction, and abdominal liposuction within 1 year prior to screening);<br>7) Planned bariatric surgery or acupuncture for weight loss, liposuction and abdominal fat removal, etc. during the study;<br>8) Have had a history of moderate to severe depression; Or have a history of severe mental illness in the past, such as schizophrenia, bipolar disorder, etc.;<br>9) Previous suicidal tendency or suicidal behavior;<br>10) PHQ (Screening Scale for Depression) questionnaire ≥ 15 points at screening or randomization;<br>11) C-SSRS (Columbia Suicide Severity Scale) questionnaire category 4 or 5 at screening or randomization;<br>12) Uncontrolled hypertension within one month prior to screening, defined as systolic blood pressure ≥ 160 mmHg and/or diastolic blood pressure ≥ 100 mmHg (stable for 1 month if using antihypertensive drugs);<br>13) History of malignancy (except cured basal cell carcinoma of the skin and carcinoma in situ of the cervix) in the past or at the time of screening;<br>14) Patients with previous myocardial infarction, angina pectoris, acute and chronic heart failure, cardiomyopathy, or cardiac surgery such as percutaneous coronary intervention, coronary artery bypass grafting, or echocardiography indicating significantly abnormal cardiac function and not suitable for participation in this study as assessed by the investigator;<br>15) Hemorrhagic or ischemic stroke or transient ischemic attack within 6 months prior to screening;

	16) History of thyroid C-cell carcinoma, MEN (multiple endocrine neoplasia) 2A or 2B, or relevant family history;<br>17) Patients with a past history of acute or chronic pancreatitis, gallbladder disease, and pancreatic injury;<br>18) Existence of limb deformity or disability, unable to accurately determine height, weight and other indicators;<br>19) Major and medium-sized surgery, severe trauma, severe infection within 1 month prior to screening, which is not suitable for participation in the study as judged by the investigator;<br>20) Anticipated surgery during the trial, except for outpatient surgery that has no effect on the safety of subjects and trial results as judged by the investigator;<br>21) Subjects who are positive for human immunodeficiency virus (HIV) antibody or hepatitis C (HCV) antibody or syphilis antibody at screening;<br>22) Positive hepatitis B surface antigen (HBsAg) and hepatitis B virus DNA ≥ 1000 IU/ml at screening (anti-hepatitis B virus drugs are not allowed to be enrolled at screening);<br>23) History of alcohol and drug abuse at screening. Average weekly alcohol intake of more than 21 units for males or 14 units for females, or unwillingness to stop drinking 24 hours before the day of dosing and throughout the study (1 unit = 360 ml of beer, or 150 ml of red wine, or 45 ml of distilled spirits/liquor).<br>5. Any of the laboratory test indicators at screening meet the following criteria (if there is a clear reason for re-test, the re-test can be performed within one week, and the investigator should record the reason for re-test):<br>1) Serum calcitonin ≥ 20 ng/L (pg/mL);<br>2) Alanine aminotransferase ≥ 3.0 × Upper Limit of Normal Value (ULN) and/or aspartate aminotransferase ≥ 3.0 × ULN and/or total bilirubin ≥ 1.5 × ULN;<br>3) Glomerular filtration rate eGFR < 60 mL/min/1. 73 m^2, estimated using the CKD-EPI equation (see Appendix 3);<br>4) Presence of thyroid dysfunction (TSH > 6 mIU/L or < 0.4 mIU/L);<br>5) Fasting triglycerides ≥ 5. 64 mmol/L (500 mg/dl);<br>6) Blood amylase or lipase > 2.0 × ULN;<br>7) International normalized ratio (INR) of prothrombin time greater than the upper limit of normal range;<br>8) Hemoglobin < 110 g/L (males) or < 100 g/L (females).<br>6. Heart rate < 50 beats/min or > 100 beats/min on 12-lead ECG at screening;

	7. The following clinically significant 12-lead electrocardiograms (ECGs) abnormalities at screening: 2nd or 3rd degree atrioventricular block, long QT syndrome, or QTcF > 500 ms (calculated as shown in Appendix 4), left or right bundle branch block, pre-excitation syndrome or other significant arrhythmia (except sinus arrhythmia);<br>8. Pregnant or lactating females, males or females of childbearing potential not willing to use contraception throughout the study;<br>9. Blood donation and/or blood loss ≥ 400 mL or bone marrow donation within 3 months prior to screening, or hemoglobinopathy, hemolytic anemia, sickle cell anemia;<br>10. The subject has any other factors that may affect the efficacy or safety evaluation of this study, and is not suitable for participation in this study in the opinion of the investigator.
**Study drug /Method of administration**	3.0 mg dose group (IBI362/placebo): 1.5 mg QW for 4 weeks + 3.0 mg QW for 20 weeks.<br>4.5 mg dose group (IBI362/placebo): 1.5 mg QW for 4 weeks + 3.0 mg QW for 4 weeks + 4.5 mg QW for 16 weeks.<br>6.0 mg dose group (IBI362/placebo): 2.0 mg QW for 4 weeks + 4.0 mg QW for 4 weeks + 6.0 mg QW for 16 weeks.<br>9.0 mg dose group (IBI362/placebo): 3.0 mg QW for 4 weeks +6.0 mg QW for 4 weeks +9.0 mg QW for 16 weeks.
**Statistical Methods**	• Sample Size:<br>For stage 1, assuming a weight loss of approximately 2.3% from baseline after 24 weeks in the placebo group, a weight loss of up to approximately 10% from baseline after 24 weeks in the IBI362 group, a standard deviation of weight loss of approximately 8%, a dropout rate of approximately 20%, an observed efficacy of 8%, and α = 0.05 (two-sided), the sample size of approximately 80 subjects per dose group (approximately 240 subjects in total) will provid a power > 95%, that at least one of the doses of IBI362 would result in superior weight loss versus pooled placebo.<br>For stage 2, assuming a body weight loss of approximately 2.3% from baseline after 24 weeks in the placebo group and 12% from baseline after 24 weeks in the IBI362 group, with a standard deviation of approximately 8%, a dropout rate of approximately 20% and an observed efficacy of approximately 10%, α = 0.05 (two-sided), a total of approximately 80 subjects will provide a power > 95%, that IBI362 9.0 mg would result in superior weight loss versus placebo.<br>• Hypothesis testing:

	Stage 1:<br>In this trial, the primary endpoint will be tested between each IBI362 dose group and placebo:<br>Mean percent change from baseline body weight after 24 weeks of IBI362 treatment: w1<br>Mean percentage change from baseline body weight in placebo group after 24 weeks: w0<br>H0: w1=w0<br>Ha: w 1 ≠ w 0<br>The mean percent change in body weight was calculated separately for each treatment group and compared to placebo. Statistical superiority of either IBI362 dose group compared to placebo in reduction of body weight from baseline after 24 weeks of dosing was considered to be established if the difference from placebo was significant ($p < 0.05$).<br>No multiplicity adjustment will be made in this trial.<br>Stage 2:<br>In this trial, the primary endpoint will be tested between IBI362 and placebo in the 9.0 mg dose group:<br>Mean percent change from baseline body weight after 24 weeks of IBI362 treatment: w1<br>Mean percentage change from baseline body weight in placebo group after 24 weeks: w0<br>H0: w1=w0<br>Ha: w 1 ≠ w 0<br>Mean percent change in body weight will be calculated for IBI362 and compared to placebo. Statistical superiority of IBI362 9.0 mg compared to placebo in reduction of body weight from baseline after 24 weeks of dosing will be considered if the difference between IBI362 and placebo is significant ($p < 0.05$).<br>• Interim Analysis:<br>In order to support the sponsor's communication with regulatory authorities, such as the IBI362 development strategy and the Phase 3 clinical study design, at least one interim analysis of efficacy and safety data will be conducted in this study before the primary endpoint visit. The results of the interim analysis will not affect the operation of the trial and the subsequent study progress, and will only be used to support the dose recommendation for phase 3 trial. The clinical study design will not be changed due to the results of the interim analysis.

	The subjects in the two stages of this study were enrolled and analyzed separately, and the operation and analysis of the two stages of the study were not affected by each other.<br>• Statistical Methods:<br>➢ Efficacy Analysis:<br>The primary efficacy endpoint of this trial is the percentage change from baseline in body weight after 24 weeks of injection. An ANCOVA model with baseline body weight as a covariate will be used to estimate the percent change from baseline in mean body weight for each IBI362 dose group compared to placebo, the difference between groups, and its 95% confidence interval.<br>For other continuous efficacy endpoints, mean ± standard deviation, maximum, minimum, and median will be summarized by group, and p-values will be calculated for each IBI362 group versus placebo using a two-sample t-test, with point estimates and 95% confidence intervals for the corresponding differences.<br>For categorical endpoints, rates within each group will be calculated and 95% confidence intervals will be calculated using Clopper-Pearson, and the chi-square test will be used to compare the difference between IBI362 and placebo groups and the corresponding 95% confidence intervals will be calculated. The odds ratio and its 95% confidence interval for each IBI362 dose group versus placebo will also be estimated for the proportion of subjects with body weight changes from baseline ≥ 5. 0% and ≥ 10. 0% using a logistic model including baseline body weight as a covariate.<br>➢ Safety Analysis:<br>The number of subjects with each AE was summarized by Medical Dictionary for Regulatory Activities (MedDRA) system organ class, preferred term, and adverse event grade, the number and percentage of subjects with each category of AE (including causality, severity, SAE, etc.) were summarized, and each category of AE was further summarized by MedDRA system organ class and preferred term.<br>➢ Laboratory tests:<br>Measured values and changes of hematology, blood biochemistry, physical condition, vital signs and other indicators before and after administration will be described using mean ± standard deviation, maximum, minimum and median by dose group, respectively. Cross classification table will be used to describe normal and abnormal changes before and after administration.<br>➢ Immunogenicity:

	The incidence of ADA and NAb to IBI362 at all follow-up visits will be summarized separately for each dose group.<br>➢ Other:<br>Describe the measured value and change value of each measurement point of ECG. Cross classification table will be used to describe the normal and abnormal changes before and after administration in each dose group. Hypoglycemic events and injection site reactions were summarized by treatment group.<br>➢ Pharmacokinetic parameters:<br>The population pharmacokinetic profile of IBI362 will be analyzed using nonlinear mixed effects modeling.<br>➢ Pharmacodynamic parameters:<br>Measured values and changes from baseline of each pharmacodynamics (PD) parameter at baseline and at each time point after administration were summarized for each treatment group, respectively, and descriptive statistical analysis was performed (mean ± standard deviation, maximum, minimum and median). PD parameters were: fasting plasma glucose, fasting insulin.<br>➢ Exploratory Analysis:<br>Exploratory indicators: high-sensitivity C-reactive protein (hsCRP), proprotein convertase subtilisin/kexin type 9 (PCSK9), fibroblast growth factor-21 (FGF-21) and adiponectin levels and their changes from baseline will be summarized and described in each treatment group at baseline and after 24 weeks of treatment.<br>Total fat content, local visceral fat content, total lean body mass as measured by dual-energy X-ray absorptiometry (DEXA), and intra-abdominal fat area (VFA), subcutaneous fat area (SFA), and total intra-abdominal fat area (TFA) as measured by MRI were summarized by treatment group at each visit, and changes from baseline after 24 weeks of dosing were compared.<br>Liver fat content as measured by MRI-PDFF will be summarized by treatment group at each visit and change from baseline after 24 weeks of dosing.

Visit Schedule

Table 1. Trial Procedures and Evaluation Table of Stage 1

	Screening Period [1]	Run-in Period [2]		Double-Blind Treatment Period															Follow-up Period				Early Withdrawal
				Titration [3]/Maintenance Treatment Period								Maintenance Treatment Period											
**Study Visit (V)**	**1**	**2**	**3**	**4**	**5**	**6**	**7**	**8**	**9**	**10**	**11**	**12**	**13**	**14**	**15**	**16**	**17**	**18**	**19**	**20**	**21**	**22**	
**Study Weeks (W)**	**-5 ~ -3**	**-2**	**-1**	**1**	**2**	**3**	**4**	**5**	**6**	**7**	**8**	**9**	**10**	**11**	**13**	**17**	**21**	**24**	**25**	**28**	**32**	**36**	
**Study Day (D)**	**-35 ~ -15**	**-14**	**-7**	**1**	**8**	**15**	**22**	**29**	**36**	**43**	**50**	**57**	**64**	**71**	**85**	**113**	**141**	**162**	**169**	**190**	**218**	**246**	
**Time Window (D)**	**/**	**± 2**	**± 2**	**± 1**	**± 1**	**± 1**	**± 1**	**± 1**	**± 1**	**± 1**	**± 1**	**± 1**	**± 1**	**± 1**	**± 2**	**± 2**	**± 2**	**± 2**	**± 2**	**± 3**	**± 3**	**± 3**	
Signed informed consent	X																						
Assign screening number	X																						
Medical History/Prior Medications	X																						
Demographics	X																						
Vital signs[4]	X	X	X	X	X	X	X	X	X	X	X	X	X	X	X	X	X	X	X	X	X	X	X
Physical Examination[5]	X			X		X		X		X		X			X	X	X		X	X	X	X	X
Inclusion/Exclusion Criteria Assessment	X																						
Assignment of randomization number[24]				▲																			
Height	X																						
Weight[6]	X	X	X	X	X	X	X	X	X	X	X	X	X	X	X	X	X	X	X	X	X	X	X
Waist circumference [7]	X		X	X				X				X			X	X	X		X	X	X	X	X
Hip circumference [8]	X		X	X				X				X			X	X	X		X	X	X	X	X
Laboratory tests[9]	X			X				X				X			X	X	X		X			X	X
Blood chemistry (including lipids) [10]				●											●				●				●

	Screening Period [1]	Run-in Period [2]		Double-Blind Treatment Period															Follow-up Period				Early Withdrawal
				Titration [3]/Maintenance Treatment Period								Maintenance Treatment Period											
**Study Visit (V)**	**1**	**2**	**3**	**4**	**5**	**6**	**7**	**8**	**9**	**10**	**11**	**12**	**13**	**14**	**15**	**16**	**17**	**18**	**19**	**20**	**21**	**22**	
**Study Weeks (W)**	**-5 ~ -3**	**-2**	**-1**	**1**	**2**	**3**	**4**	**5**	**6**	**7**	**8**	**9**	**10**	**11**	**13**	**17**	**21**	**24**	**25**	**28**	**32**	**36**	
**Study Day (D)**	**-35 ~ -15**	**-14**	**-7**	**1**	**8**	**15**	**22**	**29**	**36**	**43**	**50**	**57**	**64**	**71**	**85**	**113**	**141**	**162**	**169**	**190**	**218**	**246**	
**Time Window (D)**	**/**	**± 2**	**± 2**	**± 1**	**± 1**	**± 1**	**± 1**	**± 1**	**± 1**	**± 1**	**± 1**	**± 1**	**± 1**	**± 1**	**± 2**	**± 2**	**± 2**	**± 2**	**± 2**	**± 3**	**± 3**	**± 3**	
Blood Pregnancy/Urine Pregnancy Test[11]	X			X				X				X			X	X	X		X	X	X	X	X
Serological testing for infectious diseases [12]	X																						
12-lead ECG[13]	X	X	X	X	X	X	X	X	X	X	X	X	X	X	X	X	X	X	X	X	X	X	X
Echocardiogram	X																		X				
Glycosylated haemoglobin (HbA1c) [14]	X			●											●				●				●
OGTT[15]	X																						
Thyroid function[16]	X																		X				X
Serum calcitonin	X																		X				X
Immunogenicity tests[17]				●		●		●				●				●			●		●		●
PK[18]				●		●		●				●				●			●		●		●
PD[19]				●		●		●				●				●			●		●		●
Injection site reaction[5]				X	X	X	X	X	X	X	X	X	X	X	X	X	X	X					
Abdominal MRI20				X															X				X
Dual Energy X-Ray (DEXA) [20]				X															X				X
PCSK9, FGF21, adiponectin, hsCRP [21]				●															●				●
PHQ-9 questionnaire[22]	X			X											X				X			X	X

	Screening Period [1]	Run-in Period [2]		Double-Blind Treatment Period															Follow-up Period				Early Withdrawal
				Titration [3]/Maintenance Treatment Period								Maintenance Treatment Period											
**Study Visit (V)**	**1**	**2**	**3**	**4**	**5**	**6**	**7**	**8**	**9**	**10**	**11**	**12**	**13**	**14**	**15**	**16**	**17**	**18**	**19**	**20**	**21**	**22**	
**Study Weeks (W)**	**-5 ~ -3**	**-2**	**-1**	**1**	**2**	**3**	**4**	**5**	**6**	**7**	**8**	**9**	**10**	**11**	**13**	**17**	**21**	**24**	**25**	**28**	**32**	**36**	
**Study Day (D)**	**-35 ~ -15**	**-14**	**-7**	**1**	**8**	**15**	**22**	**29**	**36**	**43**	**50**	**57**	**64**	**71**	**85**	**113**	**141**	**162**	**169**	**190**	**218**	**246**	
**Time Window (D)**	**/**	**± 2**	**± 2**	**± 1**	**± 1**	**± 1**	**± 1**	**± 1**	**± 1**	**± 1**	**± 1**	**± 1**	**± 1**	**± 1**	**± 2**	**± 2**	**± 2**	**± 2**	**± 2**	**± 3**	**± 3**	**± 3**	
C-SSRS questionnaire[22]	X			X											X				X			X	X
IWQoL-Lite questionnaire[22]				X											X				X				X
Recording of Adverse Events and Concomitant Medications		X	X	X	X	X	X	X	X	X	X	X	X	X	X	X	X	X	X	X	X	X	X
Injecting the Subject [23]		☆	☆	★	★	★	★	★	★	★	★	★	★	★	★	★	★	★					
Diet and exercise control	X	X	X	X	X	X	X	X	X	X	X	X	X	X	X	X	X	X	X	X	X	X	
Drug Dispensing														X	X	X	X						
Drug recovery															X	X	X	X					
Diary Card Dispensing	X	X	X	X	X	X	X	X	X	X	X	X	X	X	X	X	X	X	X	X	X		
Diary Card Recycling		X	X	X	X	X	X	X	X	X	X	X	X	X	X	X	X	X	X	X	X	X	
Compliance Assessment			X	X	X	X	X	X	X	X	X	X	X	X	X	X	X	X	X				

Notes:

1. If the examination in screening period is more than 5 days from the day of administration in run-in period, it should be confirmed again within 5 days before administration, including vital signs, physical examination, hematology, blood biochemistry, blood lipid, coagulation function, urinalysis, myocardial enzyme spectrum, blood amylase, lipase, thyroid function, serum calcitonin, ECG and blood/urine pregnancy test.
2. The run-in period consisted of a placebo run-in period of 2 weeks.
3. The titration period was 4 weeks for subjects in the 3.0 mg dose group and 8 weeks for subjects in the 4.5 mg and 6.0 mg dose groups.
4. Window for vital signs: to be completed within 1 hour prior to dosing (except for non-dosing visits).
5. Physical examination: in the physical examination of skin and mucosa after administration, attention should be paid to the observation of abnormalities at the injection site. In addition to the examination time points listed in the table, the local skin and mucous membrane at the injection site should be observed for

abnormalities immediately (± 2 min), 20 min (± 5 min), 40 min (± 5 min) and 60 min (± 5 min) after administration, including but not limited to skin erythema/redness, swelling, pain/tenderness, congestion/hemorrhage, etc.

6. Body weight: The subjects were required to fasting, after urination and take off their coat during each measurement. The same subject used the same weight scale each time and avoided strenuous exercise before measurement. See Appendix 8 for details.
7. Waist circumference: (1) standing position, relaxed shoulders and abdomen, smooth breathing, feet 25-30cm apart; (2) The horizontal position of measurement: the midpoint of the line between the anterior superior iliac spine and the inferior margin of the 12th costal line on the midaxillary line; (3) Use a tape ruler around the abdomen in the above-mentioned horizontal position, with the tape ruler closely adhering to the skin, but not strangling the skin; (4) The measurement should not be made consciously with the abdomen closed or lifted, and the measurement should be taken at the calm end of expiration, with the waist circumference in cm to the nearest mm (e.g., 89.3 cm). See Appendix 9 for details.
8. Hip circumference: (1) The subject stands naturally, with shoulders relaxed, arms naturally drooping and moderately open, legs together, legs evenly loaded, hips relaxed, and eyes ahead; (2) Horizontal positions measured: symphysis pubis anteriorly and greater trochanter of femur posteriorly; (3) Generally equivalent to the most protruding part of the buttock; (4) Wrap the buttocks horizontally with a tape ruler and record the values. See Appendix 10 for details.
9. Laboratory tests include: (refer to Section 6.1.1)

（1）Hematology: White Blood Cell (WBC), Red Blood Cell (RBC), Platelet (PLT), Hemoglobin (HGB), Hematocrit (HCT), differential white blood cell count (neutrophils, basophils, eosinophils, monocytes and lymphocytes);

（2）Blood biochemistry: Aspartate Aminotransferase (AST), Alanine Aminotransferase (ALT), Total Bilirubin (TBIL), Direct Bilirubin (DBIL), Albumin (ALB), Total Protein (TP), Glutamyl Transpeptidase (GGT), Alkaline Phosphatase (ALP), Lactate Dehydrogenase (LDH), serum potassium, serum sodium, serum calcium, serum chloride, Uric Acid (UA), urea, creatinine (Cr), Fasting Blood Glucose (FBG);

（3）Blood lipids: Total Cholesterol (TC), Triglyceride (TG), High-Density Lipoprotein Cholesterol (HDL-C), Low-Density Lipoprotein Cholesterol (LDL-C)

（4）Coagulation function: Prothrombin Time (PT), Activated Partial Thromboplastin Time (APTT), International Normalized Ratio (INR);

（5）Urinalysis: urine pH, urine protein (UPRO), urine glucose (UGLU), urine red blood cell (URBC), urine white blood cell (UWBC);

（6）Myocardial enzyme spectrum: Creatine Kinase (CK), creatine kinase isoenzyme (CK-MB), troponin (cTnI or cTnT) and myoglobin;

（7）Blood amylase and lipase (if the conditions of the study site are limited, blood amylase should be tested at least).

10. On V4 (D1), V15 (D85) and V19 (D169), another 2 mL of fasting peripheral blood should be collected and sent to the central laboratory for blood biochemistry (including blood lipids).
11. Blood/urine pregnancy test is applicable to women of childbearing potential (refer to Section 4.3. 1.1 for the definition of childbearing potential), and blood pregnancy test is required at V1, V4 and V19 visits.

12. Serological detection of infectious diseases: including Human Immunodeficiency Virus (HIV) antibody, hepatitis B virus (HBV), hepatitis C virus (HCV) antibody and syphilis antibody. If hepatitis B surface antigen is positive, hepatitis B virus DNA testing is required.
13. 12-lead electrocardiogram (ECG) indicators include R interval, PR interval, Heart Rate (HR), QT interval and QTcF (refer to Appendix 4 for calculation formula). If the subject has an abnormal ECG, the investigator can perform additional ECG according to the actual situation. ECG on V4 (D1) and V19 (D169) should be performed twice consecutively with an interval of 2 ~ 10min.
14. Glycosylated hemoglobin test by central laboratory: blood collection points are V4 (D1), V15 (D85) and V19 (D169). If the subject withdraws early, one sample will be collected at the early withdrawal visit as far as possible. 2 mL of whole blood will be collected at each blood collection point under fasted state. Refer to the central laboratory manual for detailed procedures.
15. If the subjects' fasting blood glucose is 6.1-6.9 mmol/L at screening, venous blood glucose should be collected two hours after OGTT for confirmation. Refer to Appendix 5 for OGTT method.
16. Thyroid function indicators include Thyroid Stimulating Hormone (TSH), free triiodothyronine (Free Triiodothyronine, FT3) and free thyroxine (Free Thyroxine, FT4).
17. Immunogenicity sampling points: within 1h before administration at V4, V6, V8, V12 and V16, and at V19 (D169) and V21 (D218) visits. If a subject withdraws early, whenever possible, 1 immunogenicity sample will be collected at the early withdrawal visit. A total of 5 mL of whole blood will be collected at each blood collection point. Detailed procedures will be described in the central laboratory manual.
18. PK sampling points: within 1h before administration on V4 and 4 h ± 1 h after administration on V4; Within 1h before administration at V6, V8, V12 and V16, and at V19 (D169) and V21 (D218) visits. If a subject withdraws prematurely, a PK sample will be collected at the early withdrawal visit whenever possible. 3mL of whole blood will be collected at each blood collection point. Refer to the central laboratory manual for detailed operating procedures.
19. PD sampling points: Pre-dose PD sampling at V4, V6, V8, V12, V16 and all PD sampling at V19 (D169), V21 (D218) visits will be performed under fasting conditions. If a subject withdraws prematurely, a PD sample will be collected at the early withdrawal visit whenever possible. A total of 4 mL of whole blood will be collected at each blood collection point. Detailed procedures will be described in the central laboratory manual.
20. Dual-energy X-ray (DEXA) and abdominal MRI are recommended to be performed at sites where conditions permit.
    - DEXA method determination: The subject lies on the examination table, uses the DEXA standard mode, the scanning frame moves from the head side to the foot side, and scans. The scanning conditions were voltage (76 ± 3) KV and current 0.15 mA, the tube voltage of two X-rays was 38 V and 70 KV, respectively, the scanning particle range standard was 197 cm x 60 cm, the width was fixed at 60 cm, the scanning time was about 5 min, and the radiation absorbed dose was 0.002 mGy. At the end of measurement, DEXA device automatically records the parameters of total body and local visceral fat weight as well as total lean body mass. Considering different detection instruments, the parameters of local visceral fat content may include L4 region (male or female), Android

region (male only), or Gynoid region (female only). It is suggested that the adopted equipment is PRODIGY dual-energy X-ray absorptiometry (Lunar, GE, USA) or Hologic (Halloger) dual-energy X-ray absorptiometry (Halloger, USA).

- Abdominal MRI detection items include: routine upper abdominal detection items (liver, gallbladder, pancreas, spleen and kidney), abdominal body fat detection (intra-abdominal fat area, subcutaneous fat area, total abdominal fat area), MRI-PDFF.

21. PCSK9, FGF-21, adiponectin, hsCRP: blood collection points are V4 (D1) and V19 (D169), and if the subject withdraws early, one sample will be collected at the early withdrawal visit as far as possible. 5 mL of whole blood will be collected at each blood collection point. Detailed procedures will be described in the central laboratory manual.
22. PHQ-9 questionnaire (Depression Screening Scale), C-SSRS questionnaire (Columbia Suicide Severity Scale), IWQoL-Lite questionnaire (Weight Impact on Quality of Life Scale).
23. If the theoretical dose is missed for not more than 2 days, the drug can be resumed within 2 days; If the theoretical dose is missed for more than 2 days, the dose may not be administered at this visit, and the dose will be administered normally at the next visit; The interval between the two doses must be guaranteed to be 5 days or more. Administration between visits may be performed at home or in the hospital.
24. If the subject can be enrolled after the end of run-in period, the subject can be randomized between D-1 and D1.
25. In this study, fasting blood collection was required to be fasted for at least 8 hours, and administration was performed after fasting blood collection.
26. ☆ is the lead-in period drug, □ is the treatment period drug; ● for central laboratory testing, ▲ for assignment of randomization number.

Table 2. Trial Flow and Evaluation Form of Stage 2

	Screening period	Run-in Period [1]		Double-Blind Treatment Period								Follow-up Period				Early Withdrawal
				Titration/Maintenance Treatment Period			Maintenance Treatment Period									
**Study Visit (V)**	**1**	**2**	**3**	**4**	**5**	**6**	**7**	**8**	**9**	**10**	**11**	**12**	**13**	**14**	**15**	
**Test Weeks (W)**	**-5 ~ -3**	**-2**	**-1**	**1**	**3**	**5**	**9**	**13**	**17**	**21**	**24**	**25**	**28**	**32**	**36**	
**Test Day (D)**	**-35 ~ -15**	**-14**	**-7**	**1**	**15**	**29**	**57**	**85**	**113**	**141**	**162**	**169**	**190**	**218**	**246**	
**Time Window (D)**	**/**	**± 2**	**± 2**	**± 1**	**± 1**	**± 1**	**± 1**	**± 2**	**± 2**	**± 2**	**± 2**	**± 2**	**± 3**	**± 3**	**± 3**	
Signed informed consent	X															
Assign screening number	X															
Medical History/Prior Medications	X															
Demographics	X															
Vital signs[2]	X	X	X	X	X	X	X	X	X	X	X	X	X	X	X	X
Physical Examination[3]	X			X	X	X	X	X	X	X		X	X	X	X	X
Inclusion/Exclusion Criteria Assessment	X															
Assignment of randomization number				▲												
Height	X															
Weight[4]	X	X	X	X	X	X	X	X	X	X	X	X	X	X	X	X
Waist circumference [5]	X		X	X		X	X	X	X	X		X	X	X	X	X
Hip circumference [6]	X		X	X		X	X	X	X	X		X	X	X	X	X
Laboratory tests[7]	X			X		X	X	X	X	X		X			X	X
Blood chemistry (including lipids) [8]				●				●				●				●

	Screening period	Run-in Period [1]		Double-Blind Treatment Period								Follow-up Period				Early Withdrawal
				Titration/Maintenance Treatment Period			Maintenance Treatment Period									
**Study Visit (V)**	**1**	**2**	**3**	**4**	**5**	**6**	**7**	**8**	**9**	**10**	**11**	**12**	**13**	**14**	**15**	
**Test Weeks (W)**	**-5 ~ -3**	**-2**	**-1**	**1**	**3**	**5**	**9**	**13**	**17**	**21**	**24**	**25**	**28**	**32**	**36**	
**Test Day (D)**	**-35 ~ -15**	**-14**	**-7**	**1**	**15**	**29**	**57**	**85**	**113**	**141**	**162**	**169**	**190**	**218**	**246**	
**Time Window (D)**	**/**	**± 2**	**± 2**	**± 1**	**± 1**	**± 1**	**± 1**	**± 2**	**± 2**	**± 2**	**± 2**	**± 2**	**± 3**	**± 3**	**± 3**	
Blood Pregnancy/Urine Pregnancy Test[9]	X			X		X	X	X	X	X		X	X	X	X	X
Serological testing for infectious diseases [10]	X															
12-lead ECG[11]	X	X	X	X	X	X	X	X	X	X	X	X	X	X	X	X
Echocardiogram	X											X				
Glycosylated haemoglobin (HbA1c) [12]	X			●				●				●				●
OGTT[13]	X															
Thyroid function [14]	X											X				X
Serum calcitonin	X											X				X
Immunogenicity tests[15]				●	●	●	●		●			●		●		●
PK[16]				●	●	●	●		●			●		●		●
PD[17]				●	●	●	●		●			●		●		●
Injection site reaction				X	X	X	X	X	X	X	X					
Abdominal MRI[18]				X								X				X
Dual Energy X-Ray (DEXA) [18]				X								X				X

	Screening period	Run-in Period [1]		Double-Blind Treatment Period								Follow-up Period				Early Withdrawal
				Titration/Maintenance Treatment Period			Maintenance Treatment Period									
**Study Visit (V)**	**1**	**2**	**3**	**4**	**5**	**6**	**7**	**8**	**9**	**10**	**11**	**12**	**13**	**14**	**15**	
**Test Weeks (W)**	**-5 ~ -3**	**-2**	**-1**	**1**	**3**	**5**	**9**	**13**	**17**	**21**	**24**	**25**	**28**	**32**	**36**	
**Test Day (D)**	**-35 ~ -15**	**-14**	**-7**	**1**	**15**	**29**	**57**	**85**	**113**	**141**	**162**	**169**	**190**	**218**	**246**	
**Time Window (D)**	**/**	**± 2**	**± 2**	**± 1**	**± 1**	**± 1**	**± 1**	**± 2**	**± 2**	**± 2**	**± 2**	**± 2**	**± 3**	**± 3**	**± 3**	
PCSK9, FGF21, adiponectin, hsCRP [19]				●								●				●
PHQ-9 Questionnaire[20]	X			X				X				X			X	X
C-SSRS Questionnaire[20]	X			X				X				X			X	X
IWQoL-Lite Questionnaire[20]				X				X				X				X
Recording of Adverse Events and Concomitant Medications		X	X	X	X	X	X	X	X	X	X	X	X	X	X	X
Injecting the subject [21, 22]		☆	☆	★	★	★	★	★	★	★	★					
Diet and exercise control education	X	X	X	X	X	X	X	X	X	X	X	X	X	X	X	
Drug Dispensing				X	X	X	X	X	X	X						
Drug recovery					X	X	X	X	X	X	X					
Diary Card Dispensing	X	X	X	X	X	X	X	X	X	X	X	X	X	X		
Diary Card Recycling		X	X	X	X	X	X	X	X	X	X	X	X	X	X	
Compliance Assessment			X	X	X	X	X	X	X	X	X	X				

Notes:

1. The run-in period consists of placebo run-in for 2 weeks. If the subjects are assessed after the run-in period, they can be enrolled and randomized between D-1 and D1.
2. Window for vital signs: It is recommended to be completed before ECG and blood draws, and patients should sit still for at least 5 minutes before vital signs are performed. At V4 and V12, examination of the ipsilateral arm should be performed twice, with an interval of at least one minute between the two examinations.
3. Physical examination: Anal and genital examinations are allowed as appropriate.
4. Body weight: The subjects were required to fasting, after urination and take off their coat during each measurement. The same subject used the same weight scale each time and avoided strenuous exercise before measurement. See Appendix 8 for details.
5. Waist circumference: (1) standing position, relaxed shoulders and abdomen, smooth breathing, feet 25-30cm apart; (2) The horizontal position of measurement: the midpoint of the line between the anterior superior iliac spine and the inferior margin of the 12th costal line on the midaxillary line; (3) Use a tape ruler around the abdomen in the above-mentioned horizontal position, with the tape ruler closely adhering to the skin, but not strangling the skin; (4) The measurement should not be made consciously with the abdomen closed or lifted, and the measurement should be taken at the calm end of expiration, with the waist circumference in cm to the nearest mm (e.g., 89.3 cm). See Appendix 9 for details.
6. Hip circumference: (1) The subject stands naturally, with shoulders relaxed, arms naturally drooping and moderately open, legs together, legs evenly loaded, hips relaxed, and eyes ahead; (2) Horizontal positions measured: symphysis pubis anteriorly and greater trochanter of femur posteriorly; (3) Generally equivalent to the most protruding part of the buttock; (4) Wrap the buttocks horizontally with a tape ruler and record the values. See Appendix 10 for details.
7. Laboratory tests include: (refer to Section 6.1.1)

(1) Hematology: White Blood Cell (WBC), Red Blood Cell (RBC), Platelet (PLT), Hemoglobin (HGB), Hematocrit (HCT), differential white blood cell count (neutrophils, basophils, eosinophils, monocytes and lymphocytes);

(2) Blood biochemistry: Aspartate Aminotransferase (AST), Alanine Aminotransferase (ALT), Total Bilirubin (TBIL), Direct Bilirubin (DBIL), Albumin (ALB), Total Protein (TP), Glutamyl Transpeptidase (GGT), Alkaline Phosphatase (ALP), Lactate Dehydrogenase (LDH), serum potassium, serum sodium, serum calcium, serum chloride, Uric Acid (UA), urea, creatinine (Cr), Fasting Blood Glucose (FBG);

(3) Blood lipids: Total Cholesterol (TC), Triglyceride (TG), High-Density Lipoprotein Cholesterol (HDL-C), Low-Density Lipoprotein Cholesterol (LDL-C)

(4) Coagulation function: Prothrombin Time (PT), Activated Partial Thromboplastin Time (APTT), International Normalized Ratio (INR);

(5) Urinalysis: urine pH, urine protein (UPRO), urine glucose (UGLU), urine red blood cell (URBC), urine white blood cell (UWBC);

(6) Myocardial enzymes: Creatine Kinase (CK), Creatine Kinase Isoenzyme (CK-MB), Troponin (cTnI or cTnT), Myoglobin;

(7) Blood amylase, blood lipase (if the conditions of the study site are limited, blood amylase should be tested at least).

8. On V4 (D1), V8 (D85) and V12 (D169), another 2 mL of fasting peripheral blood should be collected and sent to the central laboratory for blood biochemistry (including blood lipids).
9. Blood/urine pregnancy test is applicable to women of childbearing potential (refer to Section 4.3.1.1 for the definition of childbearing potential), and blood pregnancy test is required at V4, V8 and V12 visits.
10. Serum virological test: including Human Immunodeficiency Virus (HIV) antibody, hepatitis B virus (HBV), hepatitis C virus (HCV) antibody and syphilis antibody. Hepatitis B virus DNA testing may be performed at the same time. If hepatitis B surface antigen is positive, hepatitis B virus DNA testing must be performed.
11. 12-lead electrocardiogram (ECG) indicators include RR interval, PR interval, Heart Rate (HR), QT interval and QTcF (refer to Appendix 4 for calculation formula). If the subject has abnormal ECG, the investigator can perform additional ECG according to the actual situation. ECG on V4 (D1) and V12 (D169) should be performed twice consecutively with an interval of 2 ~ 10min.
12. Central laboratory glycosylated hemoglobin test: blood collection points are V4 (D1), V8 (D85) and V12 (D169). If the subject withdraws early, one sample will be collected at the early withdrawal visit as far as possible. 2 mL of whole blood will be collected at each blood collection point under fasted state. Refer to the central laboratory manual for detailed procedures.
13. If the subjects' fasting blood glucose is 6.1-6.9 mmol/L at screening, venous blood glucose should be collected two hours after OGTT for confirmation. Refer to Appendix 5 for OGTT method.
14. Thyroid function indicators include Thyroid Stimulating Hormone (TSH), free triiodothyronine (Free Triiodothyronine, FT3) and free thyroxine (Free Thyroxine, FT4).
15. Immunogenicity sampling points: within 1h before administration at V4, V5, V6, V7 and V9, and at V12 (D169) and V14 (D218) visits. If a subject withdraws early, whenever possible, 1 immunogenicity sample will be collected at the early withdrawal visit. A total of 5 mL of whole blood will be collected at each blood collection point. Detailed procedures will be described in the central laboratory manual.
16. PK sampling points: within 1h before administration on V4 and 4 h ± 1 h after administration on V4; Within 1h before administration at V5, V6, V7 and V9, and at V12 (D169) and V14 (D218) visits. If a subject withdraws prematurely, a PK sample will be collected at the early withdrawal visit whenever possible. 3mL of whole blood will be collected at each blood collection point. Refer to the central laboratory manual for detailed operation procedures.
17. PD sampling points: Pre-dose PD sampling at V4, V5, V6, V7, V9 and all PD sampling at V12 (D169), V14 (D218) visits will be performed under fasting conditions. If a subject withdraws prematurely, a PD sample will be collected at the early withdrawal visit whenever possible. A total of 4 mL of whole blood will be collected at each blood collection point. Detailed procedures will be described in the central laboratory manual.
18. Dual-energy X-ray (DEXA) and abdominal MRI are recommended to be performed at sites where conditions permit.
    - Determination of DEXA method: The subject lies on the examination table, use DEXA standard mode, move the scanning frame from the head side to the foot side, and perform scanning. The scanning conditions were voltage (76 ± 3) KV and current 0.15 mA, the tube voltage of two X-rays was 38 V and 70

KV, respectively, the scanning particle range standard was 197 cm x 60 cm, the width was fixed at 60 cm, the scanning time was about 5 min, and the radiation absorbed dose was 0.002 mGy. At the end of measurement, DEXA device automatically records the parameters of total body and local visceral fat weight as well as total lean body mass. Considering different detection instruments, the parameters of local visceral fat content may include: L4 region (male or female), Android region (male only), or Gynoid region (female only). It is suggested that the adopted equipment is PRODIGY dual-energy X-ray absorptiometry (Lunar, GE, USA) or Hologic (Halloger) dual-energy X-ray absorptiometry (Halloger, USA).

- Abdominal MRI includes: routine upper abdominal examination (hepatobiliary, pancreatic, spleen and kidney), abdominal body fat examination (intra-abdominal fat area, subcutaneous fat area, total abdominal fat area), MRI-PDFF.

19. PCSK9, FGF-21, adiponectin, hsCRP: Blood sampling will be performed at V4 (D1) and V12 (D169), and if the subject withdraws early, one sample will be collected at the early withdrawal visit as far as possible. 5 mL of whole blood will be collected at each blood collection point. Detailed procedures will be described in the central laboratory manual.
20. PHQ-9 questionnaire (Depression Screening Scale), C-SSRS questionnaire (Columbia Suicide Severity Scale), IWQoL-Lite questionnaire (Weight Impact on Quality of Life Scale).
21. If the theoretical dose is missed for not more than 2 days, the drug can be resumed within 2 days; If the theoretical dose is missed for more than 2 days, the dose may not be administered at this visit, and the dose will be administered normally at the next visit; The interval between the two doses must be guaranteed to be 5 days or more. Administration between visits may be performed at home or in the hospital.
22. In this study, fasting blood collection was required to be fasted for at least 8 hours, and administration was performed after fasting blood collection.
23. Diet and exercise control propaganda and education: during the trial, the original diet and exercise lifestyle was maintained steadily, and binge eating and strenuous exercise were taboo.
24. ☆ is the lead-in period drug, □ is the treatment period drug; ● for central laboratory testing, ▲ for assignment of randomization number.

Study Design Diagram

Stage 1:

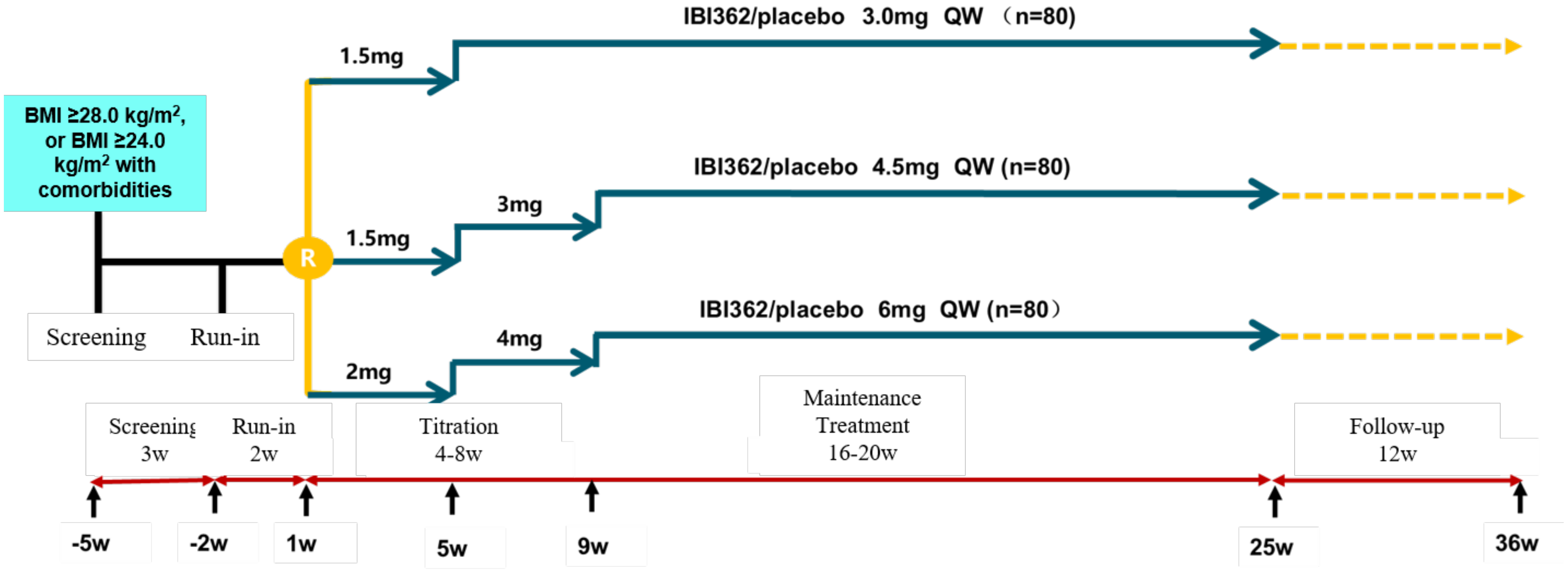

1. If the subjects in the 4.5 mg dose group still cannot tolerate the dose after reaching the specified target titration dose (4.5 mg), and the investigator considers that the patients cannot tolerate the dose for further exposure, after discussion with the sponsor, it is recommended that the dose can be reduced to 3.0 mg after reaching the specified target titration dose (4.5 mg) for 2 weeks, and the dose can be maintained until the end of the trial.

2. If the subject in the 6.0 mg dose group still cannot tolerate the dose after reaching the specified target titration dose (6.0 mg), and the investigator thinks that the patient cannot tolerate the dose for further exposure, after discussion with the sponsor, it is recommended that the dose can be reduced to 4.5 mg 2 weeks after reaching the specified target titration dose (6.0 mg) and maintained at this dose until the end of the trial.

Stage 2:

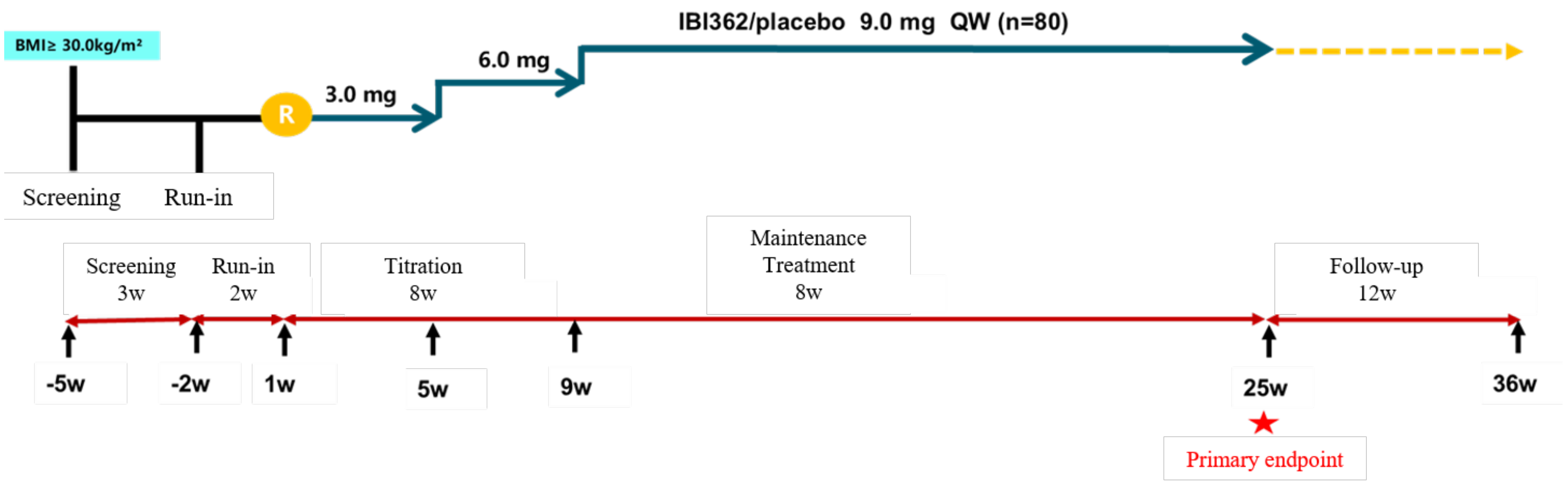

1. If the subject in the 9.0 mg dose group cannot tolerate the dose of 6.0 mg after reaching the titration dose (e.g., there is moderate to severe vomiting or diarrhea, and the gastrointestinal adverse reactions have not been relieved after symptomatic treatment and/or 1-week interruption of the dose), and the investigator considers that the patient cannot tolerate the dose for further exposure, the dose can be reduced to 3.0 mg after the discussion with the sponsor, and the dose can be re-increased to 6.0 mg 2 weeks later according to the subject's condition. If the dose is still intolerable after up-titration to 6.0 mg, the dose can be adjusted back to 3mg and maintained at this dose until the end of the study; If the 6.0 mg dose was tolerated, the dose was continued up-titrated to 9.0 mg 4 weeks later according to the subject's condition.

2. If the subject in the 9.0 mg dose group cannot tolerate the dose after reaching the specified target titration dose (9.0 mg) (e.g., there is moderate to severe vomiting or diarrhea, gastrointestinal adverse reactions remain unresolved after symptomatic treatment and/or 1-week

interruption), and the investigator considers that the patient cannot tolerate the dose for further exposure ,the dose can be reduced to 6.0 mg after the discussion with the sponsor and maintained at this dose until the end of the study or continued to be up-titrated to 9.0 mg after 2 weeks of 6.0 mg according to the subject's condition.

Figure 1. Schematic of Study Design

Content

Sponsor Signature Page **2**
Investigator Signature Page **3**
Protocol Synopsis **4**
Visit Schedule 15
Study Design Diagram 27
Content **31**
Table of Tables 34
List of Figures 34
List of Abbreviations and Definitions of Terms **35**
1. Introduction **38**
1.1. Study Background 38
1.2. Risk/Benefit Assessment 44
2. Study Objectives and Endpoints **48**
3. Study Design **51**
3.1. Overall Design 51
3.2. Study Design Rationale 52
3.3. Independent Data Monitoring Committee 53
3.4. Definition of End of Study 53
3.5. Clinical Criteria for Study Discontinuation/Early Termination 53
4. Study Population **54**
4.1. Inclusion Criteria 54
4.2. Exclusion Criteria 55
4.3. Study Restrictions and Considerations 58
4.4. Subject Screening 59
4.5. Subject discontinued treatment 59
4.6. Subject Withdrawal from the Study 60
4.7. Lost to follow-up 61
5. Study Treatment and Concomitant Therapy **61**
5.1. Treatment Regimen 61
5.2. Study drug 62
5.3. Concomitant Therapy 63
5.4. Drug Interactions 63
5.5. Treatment compliance 64
5.6. Drug management 64
5.7. Records of Study Drug 65
5.8. Complaint Handling 65
6. Study Assessments and Procedures **65**
6.1. Safety Assessments 65
6.2. Efficacy evaluation 67

6.3. Pharmacokinetic Sample Collection and Analysis 68
6.4. Pharmacodynamics 68
6.5. Immunogenicity 68
6.6. Health Outcomes/Quality of Life Assessments 69
6.7. Biomarker Specimen Collection and Assessment 69
7. Safety Reporting and Adverse Event Management 69
7.1. Definition of Adverse Events 69
7.2. Definition of Serious Adverse Events 73
7.3. Assessment of Severity of Adverse Events 74
7.4. Causal relationship judgment between adverse event and study drug 75
7.5. Recording of Adverse Events 75
8. Statistical Considerations 78
8.1. Statistical Analysis Plan 78
8.2. Hypothesis testing 78
8.3. Sample Size Estimation 79
8.4. Statistical Analysis Populations 79
8.5. Statistical Analysis Methods 80
8.6. Control of bias 84
9. Study Quality Assurance and Quality Control 85
9.1. Clinical Monitoring 85
9.2. Data Management 86
9.3. Quality Assurance Audit 87
10. Ethics 87
10.1. Ethics Committee 87
10.2. Ethical conduct in the study 88
10.3. Subject Information and Informed Consent 88
10.4. Data Protection 89
10.5. Protocol Violation 89
11. Study Management 89
11.1. Data Handling and Record Retention 89
11.2. Access to Raw Data/Documents 90
11.3. Protocol Amendment 90
11.4. Investigator Responsibilities 90
11.5. Publication Policy 90
11.6. Finance and Insurance 91
12. References 92
13. Appendices 94
Appendix 1 Protocol Amendment History 94
Appendix 2 Dyslipidemia Reference Standards 95
Appendix 3 CKD-EPI formula (eGFR estimation formula) 96

Appendix 4 Calculation formula of QTcF 97
Appendix 5 Oral Glucose Tolerance Test (OGTT) Methods 98
Appendix 6 Pancreatic enzyme monitoring and management procedures for asymptomatic patients with pancreatitis[1, 2] 99
Appendix 7 List of Causal Relationship between Adverse Events and Study Drug101
Appendix 8 SOP for Fasting Body Weight Measurement 103
Appendix 9 Waist Circumference Measurement Instructions 104
Appendix 10 Hip Circumference Measurement Instructions 105
Appendix 11 Blood Pressure Test Instructions 106

List of Tables

Table 1. Trial Procedures and Evaluation Table of Stage 1 15
Table 2. Trial Flow and Evaluation Form of Stage 2.. 21
Table 3. Effective methods of contraception (at least 1 method must be used) 58
Table 4. Treatment Regimens 61
Table 5. Routine Laboratory Test evalution 65
Table 6. Hepatic Impairment Requiring Reporting as an SAE....................................... 70
Table 7. Grading Criteria for Gastrointestinal Reactions 72

List of Figures

Figure 1. Schematic of Study Design 30
Figure 2. I8P-MC-OXAB Study: Change from Baseline in Fasting Plasma Glucose ... 45
Figure 3. I8P-MC-OXAB Study: Change from Baseline in Body Weight..................... 45
Figure 4. CIBI362B101 Study: Change from Baseline in Body Weight........................ 46
Figure 5. CIBI362B101 Study: Percent Change from Baseline in Body Weight........... 47

List of Abbreviations and Definitions of Terms

Abbreviations	Definition
ADA	Anti-Drug Antibody
AE	Adverse Event
AESI	Adverse Event of Special Interest
ALB	Albumin
ALP	Alkaline Phosphatase
ALT	Alanine Aminotransferase
ANC	Absolute Neutrophil Count
APTT	Activated Partial Thromboplastin Time
AST	Aspartate Aminotransferase
AUC	Area Under Curve
β-HCG	Β Human Chorionic Gonadotropin
BMI	Body Mass Index
CAMP	Cyclic Adenosine Monophosphate
CDCP	Center For Diseases Control And Prevention
CK	Creatine Kinase
CRA	Clinical Research Associate
CSR	Clinical Study Report
CT	Computed Tomography
Cr	Creatinine
DBIL	Direct Bilirubin
DEXA	Dual Energy X-Ray Absorptiometry
DLT	Dose Limited Toxicity
EC	Ethics Committee
ECG	Electrocardiograms
ECRF	Electronic Case Report Form
EDC	Electronic Data Capture
FBG	Fasting Blood Glucose
FDA	Food And Drug Administration
FGF21	Recombinant Human Fibroblast Growth Factor-21
FT3	Free Triiodothyronine
FT4	Free Thyroxine
GCP	Good Clinical Practice
GCGR	Glucagon Receptor
GDB	Global Burden of Disease
GGT	Glutamyl Transpeptidase
GLP-1	Glucagon Like Peptide-1,

Abbreviations	Definition
GLP-1R	Glucagon-Like Peptide-1 Receptor
HbA1c	Hemoglobin A1c
HBcAb	Hepatitis B Core Antibody
HBeAb	Hepatitis B E Antibody
HBeAg	Hepatosis B E Antigen
HBsAb	Hepatitis B Surface Antibody
HBsAg	Hepatosis B Surface Antigen
HCT	Hematocrit
HCV	Hepatitis C Virus
HDL-C	High-Density Lipoprotein Cholesterol
HsCRP	High-Sensitivity C-Reactive Protein
HIV	Human Immunodeficiency Virus
HGB	Hemoglobin
HR	Heart Rate
ICF	Informed Consent Form
IDMC	Independent Data Monitoring Committee
INR	International Normalized Ratio
IrAE	Immune-Related Adverse Event
LDH	Lactate Dehydrogenase
LDL-C	Low-Density Lipoprotein Cholesterol
MedDRA	Medical Dictionary for Regulatory Activities
MITT	Modified Intentions to Treat
MRI	Magnetic Resonance Imaging
MRI-PDFF	Magnetic Resonance Imaging Derived Proton Density Fat Fraction
MTD	Maximum Tolerated Dose
NAb	Neutralizing Antibodies
NIA	National Institute on Aging
NOAEL	No Observable Adverse Effect Level
OGTT	Oral Glucose Toleracnce Test
OXM	Oxyntomodulin
PCSK9	Proproteinconvertase Subtilisin/Kexin Type 9
PD	Pharmacodynamics
PK	Pharmacokinetics
PLT	Platelet
PT	Prothrombin Time
PTT	Partial Thromboplastin Time

Abbreviations	Definition
RBC	Red Blood Cell
SAE	Serious Adverse Event
SAP	Statistical Analysis Plan
SFA	Subcutaneous Fat Area
SOC	System Organ Class
SS	Safety Set
TBIL	Total Bilirubin
TC	Total Cholesterol
TEAE	Treatment Emergent Adverse Event
TFA	Total Intraabdominal Fat Area
TG	Triglyceride
TP	Total Protein
TRAE	Treatment-Related Adverse Event
TSH	Thyroid Stimulating Hormone
UA	Uric Acid
ULN	Upper Limited Of Normal Value
UGLU	Urine Glucose
UPRO	Urine Protein
URBC	Urine Red Blood Cell
UWBC	Urinary White Blood Cell
VFA	Intra-abdominal Fat Area
WBC	White Blood Cell
WHO	World Health Organization

1. Introduction

1.1. Study Background

IBI362 is a long-acting synthetic peptide similar to mammalian oxyntomodulin (OXM) that utilizes a fatty acyl side chain to prolong its duration of action, allowing for once-weekly administration. When administered exogenously, OXM can improve glucose tolerance and lead to weight loss. In humans, this hormone is thought to exert its biological effects by activating the Glucagon-Like Peptide-1 Receptor (GLP-1R) and Glucagon Receptor (GCGR) [1][2]. As an OXM analog, the effects of IBI362 are thought to be mediated through simultaneous activation of GLP-1R and GCGR. In addition to GLP-1R agonists having insulin secretion-promoting and blood glucose-lowering effects, it has been found that IBI362 may also have a weight-lowering effect through its effects on GCGR.

The results of single and multiple ascending dose studies of IBI 362 showed a good safety and tolerability profile, as well as a significant reduction in body weight. Therefore, the Sponsor intends to develop IBI362 in the indications of type 2 diabetes, obesity/overweight, and nonalcoholic steatohepatitis at the same time.

1.1.1. Disease Background

Obesity refers to excessive accumulation and (or) abnormal distribution of body fat, usually accompanied by weight gain. "China Adult Obesity Prevention Expert Consensus" pointed out that [3], in 2005 WHO work report estimated that there are about 1.6 billion adults (15 years of age or older) with overweight, at least 400 million adults with obesity; At least 20 million children under the age of 5 with obesity. In 2014, the WHO Global Burden of Disease (GDB) study showed that, since 1980, the proportion of adults with overweight or obesity in the world has increased by 28%, and that of children has increased by 47%. The total number of overweight and obese people in the world has increased from 857 million in 1980 to 2.1 billion in 2013, with the United States ranking first, followed by China [4]. According to the latest data from the Center for Diseases Control and Prevention (CDCP), the prevalence of adult obesity was 42.4% in 2017-2018, of which the prevalence of severe obesity was 9.2%; The prevalence of obesity in children and minors (aged 2-19 years) is 18.5%, about 13.7 million [5]. As many as 300,000 Americans die from obesity-related diseases every year, and the direct economic losses caused by obesity and inactivity account for about 9.4% of health care spending in the United States [6]. China is also facing the obesity pandemic problem. From 1992 to 2015, the overweight rate increased from 13% to 30%, and the obesity rate increased from 3% to 12%. Meanwhile, from 2002 to 2015, the overweight rate of children and adolescents increased from 4.5% to 9.6%, and the obesity rate increased from 2.1% to 6.4% [7]. China has surpassed the United States to become the world's most obese country, according to the 2015 Global Adult Weight Survey. Among them, the number of male obesity in China is 43.2 million, the number of female obesity is 46.4 million, and the number of

overweight is nearly 400 million, and the prevalence rate shows a gradually increasing trend [7].

Obesity can lead to a number of comobidities or related diseases, which can affect life expectancy or reduce the quality of life. In the more serious obese patients, cardiovascular disease, diabetes and some cancer incidence and mortality significantly increased. According to the large-scale measurement data of Chinese population, the Chinese Obesity Working Group of China Office of International Life Science Society summarized and analyzed the relationship between Body Mass Index (BMI) and the prevalence rate of related diseases. The results showed that the risk of hypertension in the subjects with BMI ≥ 24 kg/m^2 was 3-4 times higher than that in the subjects with normal weight (BMI 18.5-23.9 kg/m^2), and the risk of diabetes was 2-3 times higher than that in the subjects with normal weight [3]. Even 5-10% weight loss reduces the risk of developing type 2 diabetes, hypertension, dyslipidemia, and obstructive sleep apnea [8][9].

Lifestyle intervention is the basic treatment for overweight or obesity, but still a considerable part of patients can not lose weight for various reasons, or can not achieve the desired weight loss goal, which can consider to use drugs to assist weight loss. At present, foreign guidelines stipulate that for obese patients with body mass index (BMI) ≥ 30 kg/m^2 or BMI ≥ 27 kg/m^2 accompanied by at least one obesity concomitant disease, such as diabetes, hypertension, dyslipidemia, obstructive sleep apnea syndrome, etc., weight loss drugs can be considered. Our guidelines suggest that, on the premise of adequate diet, exercise and behavioral therapy, drug therapy can be taken for those with the following conditions: having a strong appetite and eating a large amount of meals; Complicated with hyperglycemia, hypertension, dyslipidemia and fatty liver; Combined with weight-bearing joint pain; Causing dyspnea or obstructive sleep apnea syndrome; Patients with BMI ≥ 24 kg/m^2 and the above complications or BMI ≥ 28. 0 kg/m^2 and no matter whether there are comorbidities, who cannot lose 5.0% of body weight after 3-6 months of simple diet and exercise treatment.

At present, the main drugs approved by FDA for the treatment of obesity include benzphetamine, diethylpropion, methamphetamine, phendimetrazine, phentermine, phentermine resin complex, orlistat and liraglutide injection. At present, only orlistat has been approved to be marketed in China. Although these weight-loss drugs have some weight-loss effect, the side effects are also of concern and limit their use. For example, bupropion may cause tachycardia and insomnia, phentermine may cause insomnia, dry mouth and constipation, the combination of phentermine and fenfluramine may cause primary pulmonary hypertension and heart valve insufficiency (but phentermine alone has no obvious correlation), orlistat may cause fat-soluble vitamin deficiency, flatulence, defecation urgency, steatorrhea, etc.

1.1.2. Mechanism of study drug

Glucagon-Like Peptide-1 (GLP-1) is a peptide hormone secreted from the gut that

has multiple mechanisms aimed at lowering blood glucose and weight loss, including increased glucose-dependent insulin secretion, suppression of glucagon secretion, delay of gastric emptying, and central appetite suppression.

Glucagon, a hormone secreted by pancreatic islet α cells, consists of a single-chain polypeptide 29 amino acids in length. Glucagon exerts its physiological effects by specifically binding to glucagon receptor (GCGR) on the surface of target cells in liver and kidney, activating intracellular adenylate cyclase and increasing intracellular Cyclic Adenosine Monophosphate (cAMP) level. Glucagon is a catabolic hormone, short-term injection of glucagon can promote glycogenolysis and gluconeogenesis, so that blood glucose increases. However, studies have found that long-term activation of GCGR by glucagon injection reduces appetite, stimulates fatty acid breakdown, and significantly increases energy expenditure in adipose tissue [10].

OXM is a peptide hormone secreted by human intestinal L cells after nutrient intake. OXM is a dual agonist of the glucagon-like peptide-1 receptor (GLP-1R) and glucagon receptor (GCGR), which combines the actions of GLP-1 and glucagon. Injection of OXM in humans significantly reduces body weight and appetite, and increases energy expenditure, and may be more effective than GLP-1R agonists in the treatment of obesity [11][12].

1.1.3. Study Rationale

OXM, an endogenous gut peptide hormone, combines the anorexic and hypoglycemic effects of GLP-1R agonists with the GCGR-mediated increase in energy expenditure [13][14]. GLP-1R knockout mice (GLP-1 R $^{-/-}$) showed a decrease in body weight after slow infusion of OXM, but to a lesser extent compared to wild type (WT) mice. This suggests that the weight-reducing effect of OXM requires the simultaneous activation of both GLP-1R and GCGR receptors [15]. Preclinical data from rodents suggest that GLP-1R/GCGR agonists reduce body weight more effectively than GLP-1R agonists. Lao et al Reported that their dual GLP-1R/GCGR agonist showed higher weight loss in diet-induced obese rhesus monkeys [16][17]. Preclinical data for IBI362 also showed significant weight loss (loss of appetite, weight loss) in diet-induced obese mice.

The study drug, IBI362, is an OXM analog (OXM3). Endogenous OXM is thought to activate both GLP-1R and GCGR, which play an important role in blood glucose regulation and weight loss. It was found that endogenous OXM activated GLP-1R weaklier than GCGR in cAMP signaling-related ERK1/2 phosphorylation, but activated GLP-1R and GCGR in a similar proportion in cAMP-mediated $Ca2^{+}$ influx [18]. This study drug, OXM3, adjusts the ratio of agonistic GLP-1R and GCGR to 3: 1 by modifying endogenous OXM, increasing the combined body weight-lowering effect of GLP-1 and GCG, while increasing agonistic GLP-1R does not increase the risk of hypoglycemia due to GLP-1 glucose-dependent glucose lowering.

This study is planned to assess the change from baseline in body weight after 24 weeks of IBI362 injection in subjects with overweight or obesity who are controlled with diet and exercise for at least 12 weeks and who have a weight change of less than 5.0%.

Completed single dose study in healthy subjects (I8P-MC-OXAA): A single-center, double-blind, placebo-randomized controlled SAD study (escalating doses of 0.03 mg, 0.1 mg, 0.3 mg, 1.0 mg, 2.5 mg, 5.0 mg) to assess the safety, tolerability, pharmacodynamics/pharmacodynamics (PK/PD) of IBI362 in healthy subjects. The results showed that all the subjects who were escalated to 2.5 mg were tolerable, but all the 6 subjects experienced gastrointestinal-related AE (mainly nausea and vomiting) when escalated to 5 mg. Therefore, 2.5 mg could be used as the maximum tolerated dose (MTD) after single-dose administration.

Completed multiple dose study in healthy subjects (I8P-MC-OXAB): A single-center, double-blind, placebo-randomized controlled MAD study (escalating doses of 0.05 mg, 0.2 mg, 0.5 mg, 1.5 mg) to assess the safety, tolerability, PK/PD of IBI362 in healthy subjects. The results showed that after 4 weeks of dosing, subjects in the 1.5 mg dose group were tolerable with the most pronounced body weight loss on Day 29 (-3.45 kg).

I8P-MC-OXAD, a multiple-dose study in patients with type 2 diabetes mellitus, has completed data analysis in Cohort 1 (escalating doses of 1.5 mg, 3.0 mg, 4.5 mg) and was safe and well tolerated with a mean reduction in HbA1c of 1.85%.

CIBI362B101 study was a randomized, double-blind, placebo-controlled clinical study to assess the safety, tolerability, and PK/PD profile of multiple injections of IBI362 in Chinese patients with overweight or obesity. The safety results showed that the overall safety and tolerability of the subjects were good. No Dose Limiting Toxicity (DLT) events, Serious Adverse Event (SAE) and severe adverse events occurred. Weight loss results showed that the change from baseline in body weight was significantly decreased in the IBI362 group compared with the placebo group in all cohorts. At the same time, it was found that the indicators related to comorbidities caused by obesity (fasting blood glucose, HbA1c, blood pressure, blood lipids, etc.) also significantly decreased, bringing comprehensive metabolic improvement to patients.

1.1.4. Results of preclinical studies of the study drug

Preclinical safety studies with IBI362 were conducted primarily in safety pharmacology, *in vitro* hERG, 1-and 6-month long-term toxicity studies in rats and monkeys, reproductive toxicity, and genotoxicity. In the cardiovascular, respiratory, body temperature, and neurological safety pharmacology studies conducted in male monkey telemetry cardiovascular safety pharmacology studies and in the combined 1-and 6-month repeat-dose toxicity studies, IBI362 administration resulted in significant increases in heart rate and secondary shortening of the QT interval in monkeys. The respiratory system,

body temperature, and nervous system were not significantly affected by IBI362. In an hERG channel assay *in vitro*, the IC50 for hERG inhibition was > 300 μM, and IBI362 is not expected to have an effect on ventricular repolarization via hERG inhibition. In the long-term toxicity test of 6-month continuous administration in male rats, increases in Creatine Kinase (CK) and AST were observed, which may be due to reversible changes caused by skeletal muscle damage. The above changes were not observed in the 6-month long-term toxicity test in monkeys. Decreased food consumption and body weight were observed in the test, which were expected pharmacological effects; There was no significant toxicity on fertility and early embryo development of SD rats and embryo-fetal development of SD rats and New Zealand white rabbits; The results of *in vitro* Ames test, chromosome aberration test and *in vivo* micronucleus test in rats were negative.

Based on reversible skeletal muscle damage accompanied by increased CK and AST in male rats, the 6-month toxicology (twice weekly) No Observable Adverse Effect Level (NOAEL) in male and female rats was 0.05 mg/kg and 0.1 mg/kg, respectively, corresponding to plasma exposures in rats of 0.05 and 0.1 times, respectively, the plasma exposure (AUC) in humans at 6.0 mg QW. The AUC of monkeys dosed with 0.5 mg/kg QW was 67-fold higher than that of male rats, but there were no similar changes in muscle damage in rats. The AUC at 0.5 mg/kg QW in monkeys was 3.7-fold higher than the expected AUC in humans at 6.0 mg QW.

1.1.5. Clinical study results of the study drug

As of 07 December 2021, a total of 5 clinical studies of IBI362have been completed, including three studies in China, CIBI362A101, CIBI362B101 and CIBI362B102; And a single dose escalation study of I8P-MC-OXAA and a multiple dose escalation study of I8P-MC-OXAB conducted by Eli Lilly and Company (Eli Lilly and Company, USA) overseas.

In the dose escalation study, no severe hypoglycemic events were reported during the study, no deaths occurred, and all subjects completed the study. One serious adverse reaction was asymptomatic atrial fibrillation in the single-dose trial. One case of moderate liver enzyme elevation occurred in the multiple-dose study, and the remaining subjects had mild adverse events. Adverse events reported were mainly gastrointestinal-related events and asymptomatic hypoglycaemia, which were transient. In general, an increase in pulse rate (PR) was observed in the subjects. Safety data from subjects receiving different doses of IBI362 suggest that the safety risks of IBI362 are manageable. Similar to the adverse events observed in healthy subjects, the majority of the events were related to gastrointestinal-related adverse events, which occurred as expected given the mechanism of action of GLP-1 agonists to suppress appetite via the central nervous system. In addition, there was an increase in mean heart rate in the IBI362 arm compared to the placebo arm, but no serious cardiac disorder-related adverse events were occured.

Considering the convenience of practical clinical use, a pre-filled solution for

injection formulation of IBI362 was developed in addition to the lyophilized powder formulation used in earlier studies, and the CIBI362B102 study was conducted in healthy humans to compare the safety and PK profiles of the two different formulations. Comparison of PK parameters showed that at the same dose, compared with IBI362 for injection, the median T_{max} of IBI362 for injection was 24h, and the median T_{max} of IBI362 for lyophilized powder was 72h, and the C_{max} and AUC of IBI362 for injection were increased by approximately 29% and 18%, respectively, suggesting that the difference between the two formulations was mainly in the drug absorption process. Both formulations were safe, with no subjects experiencing a Serious Adverse Event (SAE), no severe Treatment Emergent Adverse Event (TEAE), and no TEAE leading to discontinuation or withdrawal from the study. The incidences of TEAE and Treatment Related Adverse Events (TRAE) were slightly lower in the IBI362 solution for injection group (prefilled solution for injection formulation) than in the IBI362 solution for injection group (lyophilized powder formulation).

Two clinical studies in patients (CIBI362A101 and CIBI362B101), a multicenter, randomized, double-blind, placebo-controlled clinical study to assess the safety, tolerability, and PK/PD of multiple doses of IBI362 in Chinese subjects with type 2 diabetes mellitus and inadequate glycemic control (CIBI362A101) and a multicenter, randomized, double-blind, placebo-controlled clinical study to assess the safety, tolerability, and PK/PD of multiple doses of IBI362 in Chinese subjects with overweight or obesity (CIBI362B101), respectively. The data showed that IBI362 showed good glucose-lowering and weight-loss efficacy in both diabetic and obese patients. In addition, it reduces lipid, blood pressure, and liver enzyme levels, suggesting a possible overall metabolic improvement. The predominant adverse events in patients with diabetes or obesity were similar to those observed in healthy subjects, most of which were related to gastrointestinal-related adverse events and were mostly mild, given the mechanism of action of GLP-1 agonists in suppressing appetite via the central nervous system. The adverse events in the study were basically consistent with those of drugs of the same class, and there were no unexpected adverse events.

1.2. Risk/Benefit Assessment

1.2.1. Potential Risks

The results of the preclinical long-term toxicity safety study of IBI362 indicated that it may cause reversible skeletal muscle damage in male rats, but these changes were not observed in the 6-month long-term toxicity study in monkeys, and decreased food consumption and body weight loss were expected pharmacological effects.

In addition, based on the clinical safety study results of I8P-MC-OXAA study, I8P-MC-OXAB study, I8P-MC-OXAD study and domestic CIBI362B101 study, AE related to gastrointestinal reactions increased with the dose increase in dose escalation study, mainly including nausea, decreased appetite, vomiting, headache and diarrhea. Considering that most of the adverse events related to the study are gastrointestinal reactions, it is suggested that the investigator should pay special attention in the clinical study and give the corresponding symptomatic treatment to reduce the risk of the subjects using the drug. Meanwhile, the subjects with digestive system diseases should be excluded in the clinical study to avoid aggravation of the original diseases caused by drug stimulation.

1.2.2. Potential Benefits

Results from a multiple-dose study of IBI362 in healthy subjects (I8P-MC-OXAB study) showed that due to the small dose range, the maximum tolerated dose was not found, and no obvious dose-related decrease in fasting plasma glucose was found, but the fasting plasma glucose decreased significantly after 4 weeks of administration at the maximum dose of 1.5 mg compared with other low doses, as shown in Figure 2.

At the same time, after administration of 1.5 mg QW for 4 weeks, the body weight decreased by an average of 3.45 kg, and the body weight loss was most significant on Day 29 of administration, and the effect of body weight reduction was maintained until D113 (the last administration date was D29). See Figure 3 for details.

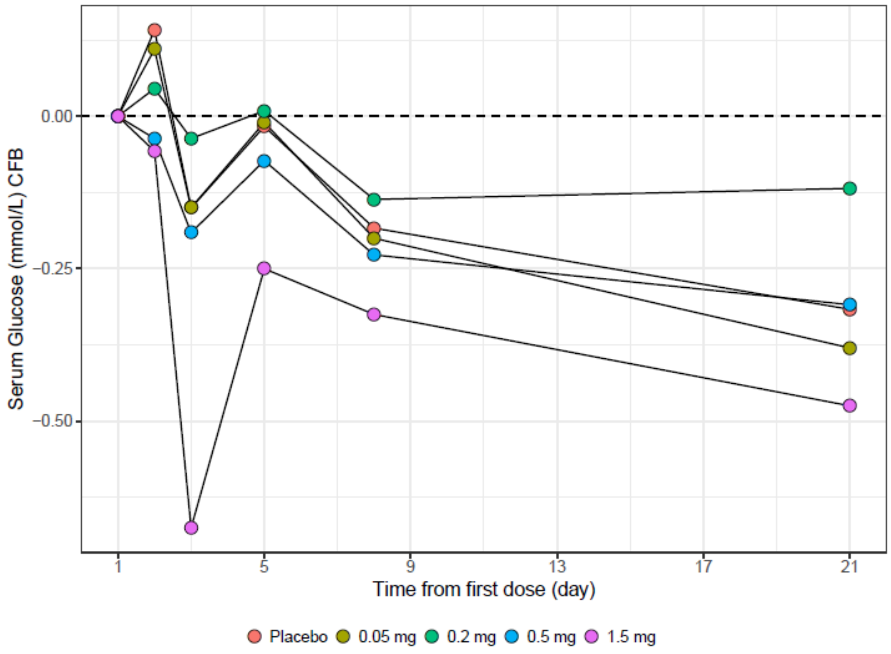

Figure 2. I8P-MC-OXAB Study: Change from Baseline in Fasting Plasma Glucose

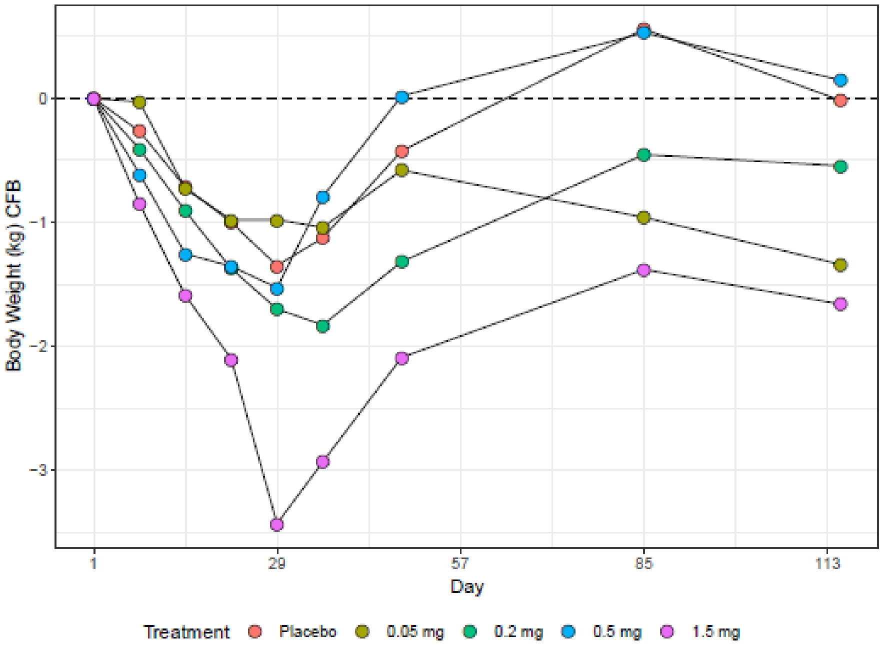

Figure 3. I8P-MC-OXAB Study: Change from Baseline in Body Weight

A Phase 1 study (CIBI362B101) of IBI362 in subjects with overweight or obesity has been completed with 5 cohorts of divided doses including:

Cohort 1: 1-4 weeks: 1.0 mg; 5-8 weeks: 2.0 mg; 9-12 weeks: 3.0 mg.

Cohort 2: 1-4 weeks: 1.5 mg; 5-8 weeks: 3.0 mg; 9-12 weeks: 4.5 mg.

Cohort 3: 1-4 weeks: 2.0 mg; 5-8 weeks: 4.0 mg; 9-12 weeks: 6.0 mg.

Cohort 4: 1-4 weeks: 2.5 mg; 5-8 weeks: 5.0 mg; 9-12 weeks: 7.5 mg; 13-16 weeks:

10.0 mg.

Cohort 5: 1-4 weeks: 3.0 mg; 5-8 weeks: 6.0 mg; 9-12 weeks: 9.0 mg.

The results showed a significant decrease in body weight change from baseline in all IBI362 dose groups compared to placebo. At week 13, the least squares mean (LS mean) (95% CI) change from baseline in body weight for subjects in the IBI362 group in Cohorts 1, 2, 3, and 5 was − 3. 80 kg (− 5. 62, − 1. 98), − 5. 72 kg (− 7. 56, − 3. 87), − 5. 02 kg (− 6. 92, − 3. 12), and − 9. 29 kg (− 11. 10, − 7. 47), with point estimates (95% CI) for differences from placebo of − 3. 46 kg (− 5. 62, − 1. 29), − 5. 38 kg (− 7. 57, − 3. 18), − 4. 68 kg (− 6. 91, − 2. 44), and − 8. 94 kg (− 11. 11, − 6. 78), respectively, At week 17, the least squares mean change from baseline in body weight for subjects in the IBI362 group in Cohort 4 was − 7. 60 kg (− 9. 78, − 5. 43) with a point estimate (95% CI) of the difference from placebo of − 6. 28 kg (− 9. 12, − 3. 44), with a nominal p-value < 0.0001. See Figure 4 and Figure 5 for details. At the same time, it was found that the indicators related to the comorbidities caused by obesity (fasting blood glucose, HbA1c, blood pressure, blood lipids, etc.) were also significantly decreased.

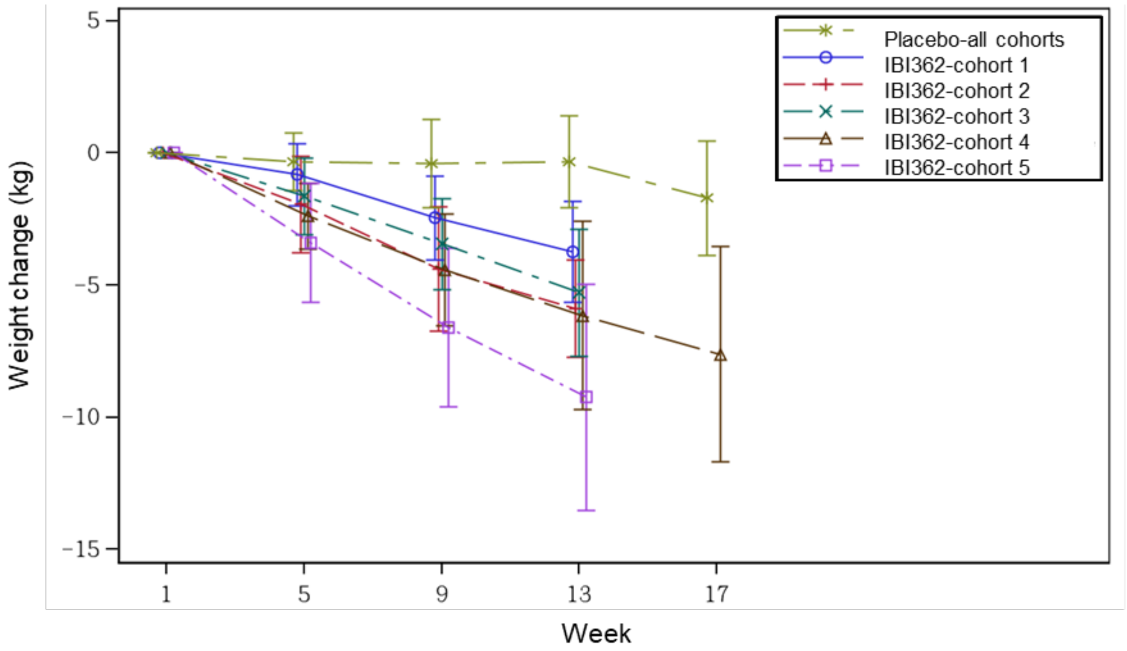

[1] Mean ± standard deviation change from baseline in body weight.

Figure 4. CIBI362B101 Study: Change from Baseline in Body Weight

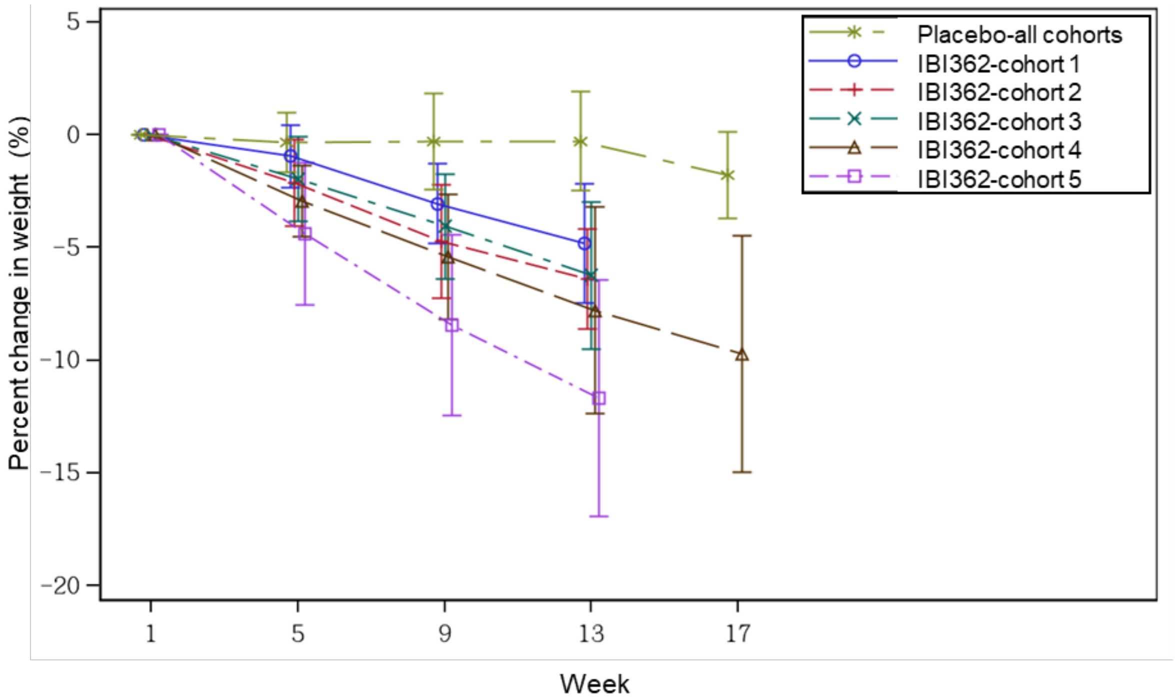

[1] Mean ± standard deviation percent change from baseline in body weight

Figure 5. CIBI362B101 Study: Percent Change from Baseline in Body Weight

2. Study Objectives and Endpoints

**Study Objectives**	**Study Endpoints**
Primary Objective	Primary Endpoint
● To evaluate the change from baseline in body weight after administration of IBI362 for 24 weeks, and to recommend the appropriate dose for Phase 3 clinical trial.	• Percent change (%) of body weight from baseline after 24 weeks of administration.
Secondary Objectives	Secondary Endpoints
● To evaluate the safety of IBI362 administered for 24 weeks.	• To evaluate the safety of IBI362 in subjects with different doses (adverse events, hypoglycemic events, vital signs, physical examination, laboratory tests, 12-lead ECG, etc.);<br>• Evaluate the mental health status of subjects after administration (C-SSRS questionnaire, PHQ-9 questionnaire);<br>• Incidence of anti-drug antibody (ADA) and neutralizing antibody (NAb) against IBI362 in serum before and after administration.
● To evaluate the changes from baseline in indicators related to comorbidities after administration of IBI362 for 24 weeks;<br>● To evaluate the rebound of body weight from baseline after administration of IBI362 for 24 weeks and 12 weeks after discontinuation.	• The proportion of subjects with body weight loss ≥ 5. 0% from baseline after administration for 24 weeks;<br>• The proportion of subjects with body weight loss ≥ 10. 0% from baseline after administration for 24 weeks;<br>• Absolute change from baseline in body weight (Kg) after administration for 24 weeks;<br>• Changes from baseline in waist circumference, BMI, glycosylated hemoglobin A1c (HbA1c), fasting plasma glucose, systolic blood pressure, diastolic blood pressure, blood lipids (TC, TG, LDL-C, HDL-C) after administration for 24 weeks;

	• Percentage change (%) of body weight from baseline after 24-week continuous administration and 12-week withdrawal;<br>• Absolute change in body weight (Kg) from baseline after 12 weeks of withdrawal after 24 weeks of administration;<br>• The proportion of subjects with body weight loss greater than 5.0% and 10% from baseline after treatment for 24 weeks and 12 weeks of discontinuation;<br>• Changes from baseline in waist circumference, BMI, HbA1c, fasting plasma glucose, systolic blood pressure, diastolic blood pressure and blood lipids (TC, TG, LDL-C and HDL-C) after 12-week discontinuation after administration for 24 weeks;<br>• To evaluate the changes from baseline in serum uric acid and alanine aminotransferase levels after 12 and 24 weeks of administration;<br>• To evaluate the improvement of quality of life (IWQoL-Lite questionnaire) after 24 weeks of administration.
● To assess the population pharmacokinetic profile and pharmacodynamic profile of IBI362 in subjects with overweight or obesity.	• To assess the population pharmacokinetic and pharmacodynamic profile of IBI362 in subjects with overweight or obesity:<br>➢ The population pharmacokinetic profile of IBI362 will be analyzed using nonlinear mixed effects modeling;<br>➢ Pharmacodynamic parameters, including changes in fasting plasma glucose and fasting insulin at different time points before and after administration.
Exploratory Objectives	Exploratory Endpoints

• To explore the effect of IBI362 on the related indicators of metabolic-related fatty liver disease;<br>• To explore the effect of IBI362 on body fat.	• To evaluate the changes of high-sensitivity C-Reactive Protein (hsCRP), Proproteinconvertase Subtilisin/Kexin Type 9 (PCSK9), Fibroblast Growth Factor-21 (FGF-21) and adiponectin levels from baseline after administration for 24 weeks;<br>• The total fat mass, regional visceral fat mass and total lean body mass measured by Dual Energy X-ray Absorptiometry (DEXA) were evaluated, and the changes of each index from baseline at week 24 of dosing;<br>• Evaluate the intra-abdominal fat area (VFA), subcutaneous fat area (SFA) and total abdominal fat area (TFA) measured by Magnetic Resonance Imaging (MRI), and the changes of each index from baseline after administration for 24 weeks;<br>• To assess liver fat content as measured by MRI-PDFF, and compae changes from baseline after 24 weeks of dosing.

3. Study Design

3.1. Overall Design

This was a multicenter, double-blind, randomized, placebo-controlled study in subjects with overweight or obesity. In the first and second stages of this study, subjects will be enrolled and analyzed separately, with no influence on each other.

Approximately 240 subjects will be enrolled in the first stage of the study, and eligible subjects will receive a 2-week placebo run-in, then will be randomized to 3.0 mg, 4.5 mg, and 6.0 mg dose groups and will be randomized in a 3: 1 ratio to receive IBI362 and placebo within each group; Randomization was stratified by BMI < 28.0 kg/m^2 and BMI ≥ 28. 0 kg/m^2 at randomization. Subjects will receive weekly dosing for a total of 24 weeks of double-blind treatment. The dose regimens are as follows: 3.0 mg dose group will be titrated from 1.5 mg, 4 weeks later (4 doses) to 3.0 mg, and the treatment will be maintained for 20 weeks; The 4.5 mg dose group should be titrated from 1.5 mg to 3.0 mg after 4 weeks (4 doses), 3.0 mg to 4.5 mg after 4 weeks, and maintained for 16 weeks; The 6.0 mg dose group should be titrated from 2.0 mg to 4.0 mg after 4 weeks (4 doses), 4.0 mg to 6.0 mg after 4 weeks, and maintained for 16 weeks.

Approximately 80 subjects are planned to be enrolled in the second stage of the study, and eligible subjects will receive a placebo run-in for 2 weeks and then be randomized at a ratio of IBI362: placebo = 3: 1 after successful run-in. Subjects were dosed weekly, starting at 3.0 mg and titrated to 6.0 mg after 4 weeks (4 doses) and 6.0 mg to 9.0 mg after 4 weeks, and maintained for 16 weeks.

The entire trial period consisted of a 3-week screening period, a 2-week placebo run-in period, a 24-week double-blind treatment period, and a 12-week off-treatment follow-up period. Subjects were required to maintain diet and exercise control throughout the study.

Dose Modification Criteria:

1. If the subject in the 4.5 mg dose group cannot tolerate the dose after reaching the specified target titration dose (4.5 mg) and the investigator considers that the patient cannot tolerate the dose for further exposure, after discussion with the sponsor, it is recommended that the dose can be reduced to 3.0 mg 2 weeks after reaching the specified target titration dose (4.5 mg) and maintained at this dose until the end of the trial.

2. If the subject in the 6.0 mg dose group cannot tolerate the dose after reaching the specified target titration dose (6.0 mg), and the investigator considers that the patient cannot tolerate the dose for further exposure, after discussion with the sponsor, it is recommended that the dose can be reduced to 4.5 mg 2 weeks after reaching the specified target titration dose (6.0 mg) and maintained at this dose until the end of the trial.

3. If the subject in the 9.0 mg dose group cannot tolerate the dose of 6.0 mg after reaching the titration dose (e.g., there is moderate to severe vomiting or diarrhea, and the gastrointestinal adverse reactions have not been relieved after symptomatic treatment and/or 1-week interruption of the dose), and the investigator considers that the patient cannot tolerate the dose for further exposure, the dose can be reduced to 3.0 mg after the discussion with the sponsor, and the dose can be re-increased to 6.0 mg 2 weeks later according to the subject's condition. If the dose is still intolerable after increasing to 6.0 mg, the dose can be adjusted back to 3mg and maintained at this dose until the end of the study; If the 6.0 mg dose was tolerated, the dose was continued increased to 9.0 mg 4 weeks later according to the subject's condition.

4. If the subject in the 9.0 mg dose group can not tolerate the dose after reaching the specified target titration dose (9.0 mg) (e.g., there is moderate to severe vomiting or diarrhea, gastrointestinal adverse reactions remain unresolved after symptomatic treatment and/or 1-week interruption), and the investigator considers that the patient cannot tolerate the dose for further exposure, the dose can be reduced to 6.0 mg after the discussion with the sponsor and maintained at this dose until the end of the study or continued to be up-titrated to 9.0 mg after 2 weeks of 6.0 mg according to the subject's condition.

3.2. Study Design Rationale

3.2.1. Rationale for Dose Selection

Four dose regimens (1.5 mg QW*4+3mg QW*20; 1.5 mg QW*4+3 mg QW*4+4.5 mg QW*16; 2 mg QW*4+4 mg QW*4+6 mg QW*16; 3 mg QW*4+6 mg QW*4+9 mg QW*16) of IBI362 are proposed to be studied in Chinese subjects with overweight or obesity based on the following considerations:

1. Proven safe dosing regimen

A Phase 1b study of IBI362 in subjects with overweight or obesity (CIBI362B101) was designed with 5 cohorts:

Cohort 1: 1-4 weeks: 1.0 mg; 5-8 weeks: 2.0 mg; 9-12 weeks: 3.0 mg.

Cohort 2: 1-4 weeks: 1.5 mg; 5-8 weeks: 3.0 mg; 9-12 weeks: 4.5 mg.

Cohort 3: 1-4 weeks: 2.0 mg; 5-8 weeks: 4.0 mg; 9-12 weeks: 6.0 mg.

Cohort 4: 1-4 weeks: 2.5 mg; 5-8 weeks: 5.0 mg; 9-12 weeks: 7.5 mg; 13-16 weeks: 10.0 mg.

Cohort 5: 1-4 weeks: 3.0 mg; 5-8 weeks: 6.0 mg; 9-12 weeks: 9.0 mg.

The safety results of the study showed that the majority of TEAE were mild, no severe TEAE were reported, no TEAE leading to permanent discontinuation, no TESAE, and no severe hypoglycemic events occurred. The overall safety and tolerability of subjects in all cohorts was good. No dose modifications of study drug occurred during

the study, and the maximum tolerated dose (MTD) was 10 mg.

2. Better weight loss

The completed Phase 1b study of IBI362 in subjects with overweight or obesity (CIBI362B101) showed that different doses of IBI362 showed significant weight reduction. At week 13 (Day 85), the least squares mean (95% CI) change from baseline in body weight for subjects in the IBI362 group was − 3. 80 kg (− 5. 62, − 1. 98), − 5. 72 kg (− 7. 56, − 3. 87), − 5. 02 kg (− 6. 92, − 3. 12), and − 9. 29 kg (− 11. 10, − 7. 47) in Cohorts 1 (1.0 mg QW*4 + 2.0 mg QW*4 + 3.0 mg QW*4), 2 (1.5 mg QW*4 + 3.0 mg QW*4 + 4.5 mg QW*4), 3（2.0mg QW*4 + 4.0mg QW*4 + 6.0mg QW*4）and 5（3.0mg QW*4 + 6.0mg QW*4 + 9.0mg QW*4）. in Cohort 4 (2.5 mg QW*4 + 5.0 mg QW*4 + 7.5 mg QW*4), the subcutaneous injection of IBI362 to 10 mg continued after the week 13, the weight continued to decline, and the weight loss effect has not yet reached the plateau. Decreases in body mass index and waist circumference were observed over the course of the study in all IBI362 dose groups.

3.2.2. Rationale for Primary Endpoint Selection

It is set according to the relevant provisions on the evaluation criteria of efficacy indicators in Technical Guidelines for Clinical Trials of Weight Control Drugs issued by CDE of CFDA in 2021, Guideline for Clinical Studies of Antiobesity Drugs issued by FDA and Guideline for Clinical Evaluation of Antiobesity Drugs (Draft) issued by EMA.

3.3. Independent Data Monitoring Committee

Not applicable.

3.4. Definition of End of Study

A subject was considered to have completed the study if he/she completed follow-up or withdrew from the study.

The end of the study is the time when the last subject completes follow-up.

3.5. Clinical Criteria for Study Discontinuation/Early Termination

The study may be suspended or prematurely terminated if there are sufficient reasons. The Party suspending or terminating the Study shall provide written notice to the Subjects, Investigators, Funding Institution and Regulatory Authorities and document the reason for the suspension or termination of the Study. If the study is prematurely terminated or suspended, the principal investigator shall promptly inform the subjects and the Ethics Committee (EC), and provide the reasons for the termination or suspension of the study. If applicable, the investigator will contact the subject and notify the subject of changes in the scheduled timing of the visit.

Situations that may require termination or suspension include, but are not limited to:

- Determine that there is an unexpected, significant or unacceptable risk to the

subject;

- The justification for effectiveness indicates that the study should be terminated/suspended;
- Inability to meet the requirements for protocol compliance;
- Incomplete and/or insufficient data for assessment.
- Planned change or discontinuation of study drug development.

The study will be continued only if issues related to safety, protocol compliance, and data quality are addressed and the requirements of the EC and/or the National Medicines Administration are met.

If the sponsor decides no longer to supply study drug, adequate notification will be made to allow appropriate adjustments to the subject's treatment.

4. Study Population

Deviations from eligibility criteria may impact the scientific integrity of the study, regulatory acceptability, and/or subject safety and are not permitted. Therefore, subjects must meet the protocol-specified criteria.

4.1. Inclusion Criteria

Subjects must meet all of the following inclusion criteria to be included in the study:

1. Age 18 ~ 75 years (both inclusive), male or female;
2. Stage 1: Obese: BMI ≥ 28. 0 kg/m^2; Or overweight: BMI 24.0 kg/m^2 < 28.0 kg/m^2 with at least one of the following comorbidites: i. Strong appetite, hunger before meal, more food intake per meal; ii. Comorbid with one or more of pre-diabetes (impaired fasting glucose and/or impaired glucose tolerance), hypertension, dyslipidemia (refer to Appendix 2 for details) and fatty liver (within 6 months prior to screening); iii. Combined weight-bearing joint pain; iv. Obesity-induced dyspnea or obstructive sleep apnea syndrome;

   Stage 2: Obese: BMI ≥ 30. 0 kg/m^2;
3. Body weight change before and after run-in period is less than 5.0%, calculation formula:

   Percent change in body weight = (weight before randomization-weight on the day of run-in)/weight before randomization * 100%;
4. Able to understand the procedures and methods of this study, willing to strictly comply with the clinical trial protocol to complete this trial, and voluntarily sign the informed consent form.

4.2. Exclusion Criteria

Subjects were excluded from the study if they met any of the following exclusion criteria:

1. The investigator suspects that the subject may be allergic to the study drug or components or drugs of the same class;
2. Weight change > 5.0% (chief complaint) controlled with diet and exercise alone for at least 12 weeks prior to screening;
3. Use of any of the following medications or treatments prior to screening:
   1) Use of GLP-1 receptor (GLP-1R) agonists or GLP-1R/GCGR agonists or GIPR/GLP-1R agonists or GIPR/GLP-1R/GCGR agonists within 3 months prior to screening;
   2) Subjects who have used drugs affecting body weight within 3 months prior to screening, including systemic steroids (intravenous, oral or intra-articular administration), tricyclic antidepressants, psychiatric drugs or sedative drugs (such as imipramine, amitriptyline, mirtazapine, paroxetine, phenelzine, chlorpromazine, thioridazine, clozapine, olanzapine, valproic acid, valproic acid derivatives, lithium salts);
   3) Patients who have used Chinese herbal medicine, health products, meal replacements that affect body weight within 3 months before screening;
   4) Have used or are currently using weight loss drugs within 3 months prior to screening, such as sibutramine hydrochloride, orlistat, phentermine, phenylpropanolamine, chlorpheniramine, phentermine, bupropion, lorcaserin, phentermine/topiramate mixture, naltrexone/bupropion mixture, etc.;
   5) Subjects who have used glucose-lowering drugs, such as metformin, SGLT2 inhibitors, thiazolidinediones (TZDs), etc. within 3 months prior to screening;
   6) Participation in other clinical trials (treated with an investigational drug) within 3 months prior to screening.
4. History or evidence of any of the following:
   1) HbA1c ≥ 6. 5% at screening or patients previously diagnosed with type 1 or type 2 diabetes;
   2) Fasting venous blood glucose ≥ 7. 0 mmol/L at screening or venous blood glucose ≥ 11. 1 mmol/L 2 hours after 75 g oral glucose tolerance test (OGTT) (for subjects with fasting blood glucose of 6.1-6.9 mmol/L at screening, venous blood glucose 2 hours after OGTT glucose load should be collected for confirmation);
   3) Patients with retinopathy in the past or at screening;
   4) Previously severe hypoglycemia or recurrent symptomatic hypoglycemia (≥ 2 times in half a year);

5) Obesity caused by secondary diseases or drugs, including increased cortisol hormone (such as Cushing's syndrome), obesity caused by pituitary gland and hypothalamus injury, obesity caused by reduction/withdrawal of weight-loss drugs, etc.;
6) Previous bariatric surgery (except acupuncture for weight loss, liposuction, and abdominal liposuction within 1 year prior to screening);
7) Planned bariatric surgery or acupuncture for weight loss, liposuction and abdominal fat removal, etc. during the study;
8) Have had a history of moderate to severe depression; Or have a history of severe mental illness in the past, such as schizophrenia, bipolar disorder, etc.;
9) Previous suicidal tendency or suicidal behavior;
10) PHQ (Screening Scale for Depression) questionnaire ≥ 15 points at screening or randomization;
11) C-SSRS (Columbia Suicide Severity Scale) questionnaire category 4 or 5 at screening or randomization;
12) Uncontrolled hypertension within one month prior to screening, defined as systolic blood pressure ≥ 160 mmHg and/or diastolic blood pressure ≥ 100 mmHg (stable for 1 month if using antihypertensive drugs);
13) History of malignancy (except cured basal cell carcinoma of the skin and carcinoma in situ of the cervix) in the past or at the time of screening;
14) Patients with previous myocardial infarction, angina pectoris, acute and chronic heart failure, cardiomyopathy, or cardiac surgery such as percutaneous coronary intervention, coronary artery bypass grafting, or echocardiography indicating significantly abnormal cardiac function and not suitable for participation in this study as assessed by the investigator;
15) Hemorrhagic or ischemic stroke or transient ischemic attack within 6 months prior to screening;
16) History of thyroid C-cell carcinoma, MEN (multiple endocrine neoplasia) 2A or 2B, or relevant family history;
17) Patients with a past history of acute or chronic pancreatitis, gallbladder disease, and pancreatic injury;
18) Existence of limb deformity or disability, unable to accurately determine height, weight and other indicators;
19) Major and medium-sized surgery, severe trauma, severe infection within 1 month prior to screening, which is not suitable for participation in the study as judged by the investigator;
20) Anticipated surgery during the trial, except for outpatient surgery that has no effect on the safety of subjects and trial results as judged by the investigator;

21) Subjects who are positive for human immunodeficiency virus (HIV) antibody or hepatitis C (HCV) antibody or syphilis antibody at screening;
22) Positive hepatitis B surface antigen (HBsAg) and hepatitis B virus DNA ≥ 1000 IU/ml at screening (anti-hepatitis B virus drugs are not allowed to be enrolled at screening);
23) History of alcohol and drug abuse at screening. Average weekly alcohol intake of more than 21 units for males or 14 units for females, or unwillingness to stop drinking 24 hours before the day of dosing and throughout the study (1 unit = 360 ml of beer, or 150 ml of red wine, or 45 ml of distilled spirits/liquor).

5. Any of the laboratory test indicators at screening meet the following criteria (if there is a clear reason for re-test, the re-test can be performed within one week, and the investigator should record the reason for re-test):

1) Serum calcitonin ≥ 20 ng/L (pg/mL);
2) Alanine aminotransferase ≥ 3.0 × Upper Limit of Normal Value (ULN) and/or aspartate aminotransferase ≥ 3.0 × ULN and/or total bilirubin ≥ 1.5 × ULN;
3) Glomerular filtration rate eGFR < 60 mL/min/1. 73 m^2, estimated using the CKD-EPI equation (see Appendix 3);
4) Presence of thyroid dysfunction (TSH > 6 mIU/L or < 0.4 mIU/L);
5) Fasting triglycerides ≥ 5. 64 mmol/L (500 mg/dl);
6) Blood amylase or lipase > 2.0 × ULN;
7) International normalized ratio (INR) of prothrombin time greater than the upper limit of normal range;
8) Hemoglobin < 110 g/L (males) or < 100 g/L (females).

6. Heart rate < 50 beats/min or > 100 beats/min on 12-lead ECG at screening;

7. The following clinically significant 12-lead electrocardiograms (ECGs) abnormalities at screening: 2nd or 3rd degree atrioventricular block, long QT syndrome or QTcF > 500 ms (calculated as shown in Appendix 4), left or right bundle branch block, pre-excitation syndrome, or other significant arrhythmia (except sinus arrhythmia);

8. Pregnant or lactating females, males or females of childbearing potential not willing to use contraception throughout the study;

9. Blood donation and/or loss of ≥ 400 mL or bone marrow donation within 3 months prior to screening, or hemoglobinopathy, hemolytic anemia, sickle cell anemia;

10. The subject has any other factors that may affect the efficacy or safety evaluation of this study, and is not suitable for participation in this study in the opinion of the investigator.

4.3. Study Restrictions and Considerations

• Subjects should not donate blood while participating in this study and for 8 weeks after the end of the study.

• Men or women of childbearing potential must use contraception during the study and for 8 weeks after the end of the study.

• Participation in other clinical trials is not allowed.

• Maintain the original diet, exercise and lifestyle stably during the trial, overeating and strenuous exercise are not allowed.

• Alcohol was prohibited during the trial.

4.3.1. Other restrictions

4.3.1.1 Childbearing age

Females of childbearing potential are defined as those who have had menarche, have not undergone sterilization (i.e., bilateral tubal ligation, bilateral salpingectomy, or total hysterectomy), and have not yet reached menopause.

Female subjects of childbearing potential who are sexually active with a non-sterilized male partner, and non-sterile male subjects who are sexually active with a female partner of childbearing potential must use at least 1 of the acceptable effective methods of contraception listed in Table 3 from the time of Screening until 8 weeks after the end of the study and should discuss the discontinuation of contraception with a responsible physician after that time point. Periodic abstinence, rhythm methods, and extracorporeal sperm withdrawal methods are not acceptable methods of contraception.

Table 3. Effective methods of contraception (at least 1 method must be used)

Barrier method	IUD method
Male condom with spermicide	With copper T-ring
Diaphragm plus spermicide	None
Diaphragm plus spermicide	None

Women were considered postmenopausal after 12 months of menopause without an alternative medical cause. The requirements according to age are as follows:

- Women > 50 years of age are considered postmenopausal if they have been amenorrheic for 12 months or more after cessation of exogenous hormone therapy and their luteinizing hormone and follicle-stimulating hormone levels are within the accepted postmenopausal range.
- Women ≤ 50 years of age were considered postmenopausal if they had been amenorrheic for 12 months or more after cessation of all exogenous hormonal

therapy, had had radiation-induced oophorectomy with the last menses occurring > 1 year earlier, had had chemotherapy-induced amenorrheic with the last menses > 1 year apart, or had undergone surgical sterilization (bilateral oophorectomy or hysterectomy).

4.3.1.2 Pregnancy

No studies have been conducted to determine whether IBI362 crosses the placental barrier and is not recommended during pregnancy. Pregnant women cannot be enrolled in this study.

4.3.1.3 Lactation

It is not known whether IBI362 is excreted in breast milk. Given that many drugs are present in human milk, breastfeeding lactating women cannot be enrolled in this study.

4.4. Subject Screening

4.4.1. Enrollment Procedure

The investigator will enroll subjects as follows:

1. Obtain informed consent with the subject's signature prior to any study-related procedures.

2. Subject eligibility will be formally determined by the Principal Investigator or appropriately trained designee after reviewing the inclusion/exclusion criteria.

The sponsor will monitor enrollment at each dose to ensure that the sample size at each dose meets the study requirements.

Patients who do not meet the relevant criteria for this study (screen failures) may be re-screened. If re-screening of a patient is considered, the investigator must contact the sponsor medical monitor. Each patient may be re-screened once after 1 month. At the time of re-screening, the patient must re-sign the Informed Consent Form (ICF) and will be re-assigned an identification number.

4.4.2. Handling Procedures for Incorrectly Enrolled Subjects

The inclusion criteria must be strictly followed. If a subject is found to be enrolled that does not meet the eligibility criteria, the sponsor's clinical study physician and the investigator will discuss whether to continue the subject in the study, with or without the study drug.

4.5. Subject discontinued treatment

Occurrence of adverse events that the investigator considers to require discontinuation of treatment, including but not limited to: severe gastrointestinal adverse reactions, which are considered to be pharmacodynamic related and persistently unrelieved after symptomatic treatment. In addition to dose reduction according to dose adjustment criteria, the investigator may consider the subject to discontinue treatment in

combination with specific clinical conditions; Pancreatitis confirmed by clinical symptoms or imaging examination; Serious arrhythmia, etc.

Discontinuation of study treatment does not mean withdrawal from the study.

Since some data on clinical events after discontinuation of treatment may be important for the study, this information must be collected until the subject's last scheduled visit, even if the subject has discontinued treatment.

Subjects may discontinue treatment at any time for any reason, or at the discretion of the investigator in the event of any adverse event. In addition, the investigator or sponsor may terminate a subject's treatment if the subject is unsuitable for treatment, violates the protocol, or for administrative and/or other safety reasons.

For subjects who discontinue treatment but continue to be monitored in the study, at least relevant examinations as specified at week 25 should be completed, including but not limited to weight, waist circumference, safety examination, etc.

4.6. Subject Withdrawal from the Study

A subject may withdraw consent at any time for any reason or be withdrawn from the study at the discretion of the investigator. In addition, the investigator or sponsor may request that a subject be withdrawn from the study if enrollment into the study is not appropriate, the protocol is violated, or for administrative and/or other safety reasons.

Including the subject's own withdrawal and the investigator's recommendation to withdraw the subject:

- The investigator considers that the subject is not suitable to continue to participate in the trial due to the occurrence of adverse events that require discontinuation of the treatment in the opinion of the investigator;
- Subjects who have poor compliance, no longer receive medication or tests before completing all the trials, and cannot insist on completing the trial as planned, including subjects who are unable to control diet well, fail to take medication as prescribed, or have other factors that may affect the efficacy observation;
- Participants in other clinical trials during the trial, and participation in other trials is defined as signing the informed consent form for other trials;
- Withdrawal of informed consent by the subject;
- Unblinding due to various reasons;
- Female subject is pregnant;
- The investigator considers that it is not suitable to continue to participate in this clinical trial.

The reason for withdrawal of a subject from the study should be recorded in the Electronic Case Report Form (eCRF).

When a subject discontinues/withdraws from the study, the subject should be asked to return to the study site for early withdrawal visit procedures, including: physical examination; Measurement of vital signs; Body weight, waist circumference and hip circumference; Laboratory tests (refer to Section 6.1.1); 12-lead ECG; Serum calcitonin; Pregnancy testing (for women of childbearing potential, refer to Section 4.3.1.2); HbA1c; DEXA; Abdominal MRI; Assessment of adverse events; Record concomitant medication; PCSK9, FGF-21, adiponectin, hsCRP blood collection; Blood collection for immunogenicity; PK/PD blood sampling; Any adverse events occurring at the time of discontinuation/withdrawal should be followed up according to safety requirements.

4.7. Lost to follow-up

Subjects will be considered lost to follow-up if they do not return to the study site for at least 3 scheduled visits and cannot be contacted by study site personnel.

If a subject does not return to the study site for a specified study visit, the following actions must be taken:

- The site attempted to contact the subject, reschedule missed visits, explain to the subject the importance of adhering to the visit schedule, and confirm if the subject is willing and/or should continue in the study.
- Prior to the subject being deemed lost to follow-up, the investigator or designee will make every effort to recontact the subject (three phone calls if possible, a certified letter to the subject's most recent mailing address if necessary, or a valid local contact information). These attempts to contact the subject should be documented in the subject's medical record or study file.

If a subject still cannot be contacted, he/she will be considered lost to follow-up and withdrawn from the study.

5. Study Treatment and Concomitant Therapy

5.1. Treatment Regimen

An overview of the treatments used in this trial is provided in Table 4 below.

Table 4. Treatment Regimens

Therapeutic drug	Study Assignment	Dose, Route, and Period	Frequency of administration	Method of administration
**IBI362/Placebo**	3.0 mg dose group	1.5 mg administered for 4 weeks + 3.0 mg for 20 weeks	QW	Abdominal subcutaneous injection
	4.5 mg dose group	1.5 mg for 4 weeks + 3.0 mg for 4 weeks + 4.5 mg for 16 weeks		

Therapeutic drug	Study Assignment	Dose, Route, and Period	Frequency of administration	Method of administration
	6.0 mg dose group	2.0 mg for 4 weeks + 4.0 mg for 4 weeks + 6.0 mg for 16 weeks		
	9.0 mg dose group	3.0 mg for 4 weeks + 6.0 mg for 4 weeks + 9.0 mg for 16 weeks.		

Note: For ease of dosing, the protocol allowed a deviation of ± 5% from the calculated total dose per infusion.

5.2. Study drug

5.2.1. Description of Study Drug

IBI362 drug product is IBI362 injection, the main active ingredient is IBI362, and excipients include trometamol, mannitol, propylene glycol (for injection), disodium edetate, dilute hydrochloric acid, sodium hydroxide, and water for injection. The dosage form is injection and the mode of administration is subcutaneous injection. IBI362 solution for injection is packaged in a pre-filled pen containing a pre-filled syringe assembly and a bromobutyl rubber plunger for the pre-filled syringe; Auto-injector components are used as secondary packaging materials. Presentation: 0.5 ml: 1.5 mg (pre-filled pen), 0.5 ml: 2mg (pre-filled pen), 0.5 ml: 3mg (pre-filled pen), 0.5 ml: 4mg (pre-filled pen), 0.5 ml: 6mg (pre-filled pen), 0.5 ml: 9mg (pre-filled pen).

Placebo: The IBI362 placebo formulation is identical to the IBI362 drug product, contains no active ingredient and consists of the excipients trometamol, mannitol, propylene glycol (for injection), edetate disodium, dilute hydrochloric acid, sodium hydroxide and water for injection. The dosage form is injection and the mode of administration is subcutaneous injection. It is packaged as a pre-filled pen-injector, the primary packaging material is a pre-filled syringe assembly and a bromobutyl rubber plunger for pre-filled syringe; Auto-injector components are used as secondary packaging materials. Presentation: 0.5 ml in pre-filled pen.

Storage condition and shelf life: Store at 2 ~ 8 °C. The validity period is tentatively set as 36 months.

Manufacturer: Innovent Biologics (Suzhou) Co., Ltd.

5.2.2. Study Drug Packaging and Labeling

The study drug IBI362 and placebo will be packaged in boxes of 1 vial each. Protocol number, drug code, drug number, strength, quantity, batch number, expiry date, storage conditions, main ingredients, dosage and administration, and information of the sponsor are printed on the packaging box of the study drug.

5.2.3. Preparation and use of study drug

This product is administered subcutaneously in the abdomen.

The investigator should ensure that the pharmacist or study nurse will use the study drug according to the protocol-specified method and dose.

5.3. Concomitant Therapy

Medications that the subject is receiving at the time of enrollment or during the study should be recorded. Such as reason for use, date of administration (including start and end dates), dose information (including dose and frequency), etc. The medical monitor should be contacted if there are any questions regarding concomitant or prior therapy.

5.3.1. Prohibited Concomitant Therapy

The following medications and measures are contraindicated:

- Growth hormone and its analogues;
- Any systemic corticosteroid (including intravenous, oral, intra-articular) administered for ≥ 7 days. Corticosteroid hormone: Mainly for glucocorticoid hormone, including short-acting: hydrocortisone, cortisone; Intermediate effect: prednisone, prednisolone, methylprednisone, triamcinolone; Long-acting: dexamethasone, betamethasone, etc.;
- Drugs for weight control, such as sibutramine hydrochloride, orlistat, phentermine, phenylpropanolamine, chlorpheniramine, phentermine, bupropion, lorcaserin, phentermine/topiramate mixture, naltrexone/bupropion mixture as well as some health products with "weight loss" indications and meal replacements;
- Drugs that have an effect on body weight: tricyclic antidepressants, drugs for psychiatric disorders and neuroleptics such as imipramine, amitriptyline, mirtazapine, paroxetine, phenelzine, chlorpromazine, thioridazine, clozapine, olanzapine, valproic acid, valproic acid derivatives, lithium salts; Antidiabetic agents: metformin, SGLT2 inhibitors, GLP-1 receptor agonists, and other agents that cause weight gain or loss;
- Any drug or herbal medicine known to have common toxic effects on major organs, or any drug that may interfere with the interpretation of efficacy and safety data.

5.4. Drug Interactions

There are no data on drugs interacting with IBI362.

5.5. Treatment compliance

During the study, subjects were required to return their unused study drug and completed diaries to assess subject dosing compliance. Dosing compliance should be emphasized every time study drug is dispensed. When study drug is returned, the subject's dosing compliance should be assessed based on the number of conversations with the subject and the amount of study drug returned. The investigator (or designee) will record in the source documents the amount of study drug dispensed and returned at each visit, as well as the reason for non-compliance. If 4 or more doses of study drug are intentionally missed or if more than the prescribed dose is repeatedly taken, compliance will be considered poor and the subject will be permanently discontinued from study drug. If the drug is discontinued once during the study due to any reason, the next dose can be administered at the dose specified in the protocol; If the drug is discontinued for more than 1 time, it should be decided whether the dose needs to be adjusted after communication with the sponsor according to the actual situation (dose and time of discontinuation, etc.).

5.6. Drug management

5.6.1. Study Drug Receipt and Accountability

IBI362 will be provided by the sponsor according to the anticipated enrollment plan at the study site. IBI362 will be shipped to the site via a third party shipping qualified logistics company. Authorized personnel at the site will sign the dispatch form to acknowledge receipt of the drug.

IBI362 will only be used in this study and will only be administered by personnel authorized by the investigator. In order to fully control the distribution and use of IBI362, the quantity will be registered at each visit.

5.6.2. Storage and Management of Study Drug

IBI362 should be refrigerated at 2 ~ 8 °C, not frozen, and should be transported to each study site in a cold chain for storage and distribution by a specially-assigned person.

The study drug should be stored in a refrigerator that can only be opened by authorized personnel. After receiving the drug, the investigator should confirm that the transportation temperature of the drug is within the specified range, and sign for receipt after checking, and store the drug at the specified temperature. In case of abnormal temperature during transportation or storage in the study site, the drug should be transferred to the specified temperature as soon as possible, and temporarily not used by the subjects, and timely reported to the sponsor, and disposed of according to the sponsor's opinions.

All investigational drugs provided by the Sponsor will be used only for this

investigational study and not for purposes other than those specified in this protocol. The investigator must undertake not to supply the study drug to any person unrelated to the study.

5.6.3. Return and Destruction of Study Drug

In this study, used containers of IBI362 need to be recycled.

All unused study drugs should be returned to the sponsor for destruction after completion/termination of the study or expiration of the expiration date. The clinical research associate designated by the sponsor will be responsible for arranging the recovery of the study drug.

5.7. Records of Study Drug

The designated personnel of the study site should timely record the receipt, distribution, use, inventory, destruction, recovery and damage of the study drug according to the requirements of relevant regulations and guidelines.

5.8. Complaint Handling

In order to ensure the safety and quality of monitoring of study participants, and to assist in process and drug improvement, the sponsor will collect product complaints related to the study drug used in the clinical trial.

Complaints related to concomitant drugs will be reported directly to the manufacturer according to the product description.

The Investigator or his/her designee is responsible for completing the following product complaint process as specified in this study:

- A study-specific complaint form was used to document the reported product complaints and the associated complete description.
- Fax or email the completed Product Complaint Form to the Sponsor or its designee within 24 hours.
- If the investigator is required to return the product for investigation, the investigator should return a copy of the product complaint form with the product.

6. Study Assessments and Procedures

6.1. Safety Assessments

6.1.1. Laboratory Tests

6.1.1.1. Routine Laboratory Safety Assessments

Table 5. Routine Laboratory Test evalution

Blood routine	RBC, HGB, HCT, WBC, PLT, differential white blood cell count (LYM, ANC, MONO, EOS, BASO)

Blood biochemistry	Liver function (TBIL, DBIL, ALT, AST, GGT, ALP, ALB, TP, LDH), renal function (BUN, UA, Cr), blood electrolytes (Na, K, Cl, Ca), FBG
Blood lipids	TC, TG, HDL-C, LDL-C
Coagulation function indicators	PT, APTT, INR
Urinalysis	Urine pH, urine protein, urine glucose, urine red blood cells, urine white blood cells
Myocardial enzyme spectrum	CK, CK-MB, cTnI or cTnT, myoglobin
Other [1]	Blood amylase, blood lipase
Viral serology	HBsAg, HBsAb, HBcAb, HBeAb, HBeAg, HCV antibody, HIV antibody, syphilis antibody
Thyroid function indicators	TSH, FT3, FT4

[1] At a minimum, a blood amylase test is required if site conditions are limited

RBC: red blood cell count; HGB: hemoglobin; HCT: hematocrit; WBC: white blood cell count; PLT: platelet count; LYM: lymphocytes; ANC: neutrophil; MONO: monocytes; EOS: eosinophils; BASO: basophils.

TBIL: serum total bilirubin; DBIL: direct bilirubin; ALT: alanine aminotransferase; AST: aspartate aminotransferase; GGT: aminoacyl transpeptidase; ALP: alkaline phosphatase, ALB: albumin; TP: total protein; LDH: lactate dehydrogenase; UA: uric acid; BUN: urea; Cr: creatinine; FBG: fasting serum glucose.

TC: total cholesterol; TG: triglycerides; HDL-C: high density lipoprotein cholesterol; LDL-C: low density lipoprotein cholesterol.

PT: prothrombin time; APTT: activated partial thromboplastin time; INR: international normalized ratio.

CK: creatine kinase; CK-MB: creatine kinase isoenzyme; CTnI: troponin I; CTnT: troponin T.

HBcAb: hepatitis B core antibody; HBeAb: hepatitis B E antibody; HBeAg: hepatitis B E antigen; HBsAb: hepatitis B surface antibody; HBsAg: hepatitis B surface antigen; HCV: hepatitis C virus; HIV: human immunodeficiency virus.

TSH: thyroid stimulating hormone, FT3: free triiodothyronine, FT4: free thyroxine.

6.1.1.2. Pregnancy test

For women of childbearing potential (as defined in Section 4.3.1.1), a serum human chorionic gonadotropin (β-HCG) or urine pregnancy test will be performed according to the schedule of visits in Table 1 and Table 2. If the result is positive, the subject is not eligible/must be discontinued. In addition to the time scheduled in the visit form, a blood

pregnancy test should be performed if pregnancy is suspected during the study.

6.1.2. Clinical examination

6.1.2.1. Physical examination

A complete physical examination will include general condition, respiratory, cardiovascular, abdomen, skin, head and neck (including ears, eyes, nose, and throat), lymph nodes, thyroid, musculoskeletal (including spine and extremities), genital/anal, and neurological assessments. Anal and genital examinations are allowed not to be performed according to the actual situation.

Refer to Visit Schedule Table 1 and Table 2 for examination time.

6.1.2.2. Vital Signs

Vital signs will be performed as described in Visit Schedule Table 1 and Table 2. Vital signs include temperature, pulse, respiratory rate, and blood pressure.

6.1.2.3. 12-lead ECG

Resting 12-lead ECGs will be analyzed locally according to Visit Schedule Table 1 and Table 2.

A 12-lead ECG will be performed after the subject has rested in a recumbent position for at least 5 minutes. All 12-lead ECGs should be recorded while the subject is resting in a recumbent position. Further ECGs will be performed when clinically indicated, e.g. In the event of a cardiac-related adverse event. The investigator completed the ECG assessment on the day of the examination and recorded the assessment on the ECG. The same method of assessment should be used throughout the study.

The investigator should assess all ECGs according to the clinically significant abnormal/not clinically significant abnormal category. In case of clinically significant abnormal findings, the investigator should record the findings as AE in the eCRF.

6.2. Efficacy evaluation

6.2.1. Primary efficacy evaluation indicators

At the primary endpoint, percent change from baseline in body weight and change from baseline in waist circumference and BMI.

6.2.2. Functional Imaging DEXA and Abdominal MRI

DEXA will be used to measure total fat content, local visceral fat content, and total lean body mass and to compare changes from baseline at 24 weeks of dosing to explore the effect of IBI362 on body fat.

MRI was used to measure intra-abdominal fat area (VFA), subcutaneous fat area (SFA) and total abdominal fat area (TFA), and the changes of each index from baseline after 24 weeks of administration were compared.

MRI-PDFF is used to measure and evaluate the liver fat content, and compare the change from baseline after 24 weeks of administration.

6.2.3. Relevant indicators for efficacy evaluation

HbA1c test: used as a reference standard for the subject's three-month average glycemic control.

Glucose load test (OGTT): If the subjects' fasting blood glucose is 6.1-6.9 mmol/L at screening, venous blood glucose should be collected two hours after OGTT for confirmation. See Appendix 5 for the specific operating procedures.

6.3. Pharmacokinetic Sample Collection and Analysis

6.3.1. Sample Collection

PK sampling will be performed according to the Visit Schedule Table 1 and Table 2 and will be performed at a central laboratory. 3 mL of whole blood was collected into anticoagulant vacutainers for PK analysis of IBI362. Details of sampling methods, sample storage, transportation and analysis were provided in the Laboratory Manual provided by the central laboratory designated by the sponsor.

6.3.2. Determination method of plasma drug concentration

The concentrations of IBI362 in plasma were determined using a validated liquid chromatography tandem mass spectrometry (LC-MS/MS) method and tested by the central laboratory designated by the Sponsor. All subjects will be required to collect whole blood samples at the blood collection points specified in the protocol to detect the plasma drug concentration.

6.4. Pharmacodynamics

6.4.1. Sample Collection

Pharmacodynamic sampling will be performed according to Visit Schedule Table 1 and Table 2, and tests will be performed at a central laboratory. 4 mL of whole blood will be collected for all PD measures (fasting insulin/fasting plasma glucose). Details of sampling methods, sample storage, transportation and analysis were provided in the Laboratory Manual provided by the central laboratory designated by the sponsor.

6.5. Immunogenicity

Immunogenicity sampling will be performed according to the Visit Schedule Table 1 and Table 2, and testing will be performed at a central laboratory. 5 mL of whole blood will be collected for immunogenicity testing. Details of sampling methods, sample storage, transportation and analysis were provided in the Laboratory Manual provided by the central laboratory designated by the sponsor.

Each subject will be tested for ADA, and ADA positive serum specimens will continue to be tested for NAb.

6.6. Health Outcomes/Quality of Life Assessments

The PHQ-9 questionnaire (Depression Screening Scale) and the C-SSRS questionnaire (Columbia Suicide Severity Scale) were used to assess the mental health status of subjects after continuous drug administration; The IWQoL-Lite questionnaire (Impact of Weight on Quality of Life Scale) was used to assess the improvement in the subject's quality of life.

6.7. Biomarker Specimen Collection and Assessment

6.7.1. Sample Collection

The biomarkers in this study include: PCSK9, FGF21, adiponectin and hsCRP. The changes of parameters related to metabolic fatty liver disease and indicators related to glucose metabolism will be monitored by collecting blood samples and detecting the changes of markers.

An additional 2mL of peripheral blood should be collected on D1, D85 and D169 according to Visit Schedule Table 1 and Table 2, and the serum should be separated, dispensed, cryopreserved and transported to the central laboratory for blood biochemistry (including blood lipid) test.

Details of sampling methods, sample storage, transportation and analysis were provided in the Laboratory Manual provided by the central laboratory designated by the sponsor.

6.7.2. Storage and Destruction of Biological Samples

Samples will be disposed of or destroyed and consolidated and anonymized. Additional analyses may be performed on anonymized, pooled samples to further evaluate and validate the analytical method. Any results obtained from these analyses may be reported separately from the Clinical Study Report (CSR).

Incurred sample reproducibility analysis, if performed, will be performed concurrently with the bioanalysis of incurred samples. The results of these assessments will not be reported in the CSR but will be presented separately in a bioanalytical report.

6.7.3. Unscheduled Visit

An unscheduled visit may be performed at the request of the patient or investigator. The investigator will perform relevant examinations according to the patient's condition, including but not limited to vital signs, targeted physical examination, hematology/blood biochemistry/urinalysis and imaging evaluation. All unscheduled visit test results should be recorded in the eCRF.

7. Safety Reporting and Adverse Event Management

7.1. Definition of Adverse Events

An adverse event (AE) is defined as any untoward medical occurrence in a clinical

trial subject starting with the signing of the informed consent form, whether or not causally related to the study drug, which is considered to be an AE, including but not limited to the following:

- Exacerbation of pre-existing (before entering the clinical trial) medical condition/disease (including aggravation of symptoms, signs, laboratory test abnormalities);
- Any newly occurring untoward medical condition (including symptoms, signs, newly diagnosed diseases);
- Abnormal clinically significant laboratory values or results.

7.1.1. Adverse Events of Special Interest

The following adverse events were of special interest in this study: allergic reactions, injection site reactions, transaminase elevations, creatinine elevations, QTc prolongation, gastrointestinal reactions (nausea, vomiting, diarrhoea), decreased appetite, dizziness, hypoglycemic events, acute pancreatitis, arrhythmia. Investigators should pay close attention to the occurrence of the above adverse events in the clinical study, and timely handle them.

7.1.1.1. Hepatic function abnormal events

Abnormalities in AST and/or ALT levels accompanied by abnormally elevated total bilirubin levels that meet the conditions in Table 6 and have no other causes of liver injury will be considered as drug-induced liver injury. Such situations should always be considered important medical events.

Table 6. Hepatic Impairment Requiring Reporting as an SAE

**Baseline Period**	Normal (AST/ALT and total bilirubin)	Abnormal (AST/ALT and total bilirubin)
**Treatment Period**	ALT or AST ≥ 3 × ULN<br>With total bilirubin ≥ 2 × ULN<br>And alkaline phosphatase ≤ 2 × ULN<br>And no hemolysis	AST or ALT ≥ 8 × ULN<br>Concomitant total bilirubin increase ≥ 1 × ULN or total bilirubin value ≥ 3 × ULN

Subjects should return to the study site for evaluation as soon as possible (preferably within 48 hours) after learning of an abnormal result. The evaluation should include laboratory tests, detailed medical history and physical assessment, and the possibility of liver neoplasia (primary or secondary) should be considered. In addition to repeat AST and ALT, laboratory tests to be performed should include ALB, CK, TBIL, direct and indirect bilirubin, GGT, PT/INR, and alkaline phosphatase. At the same time, collect detailed medical history, including: history of drinking, acetaminophen, soft drugs, various supplements, traditional Chinese medicine, chemical drug contact history, family history, occupational exposure, sexual behavior history, travel history, history of contact

with patients with jaundice, surgery, blood transfusion, history of liver disease or allergic disease, history of heart disease, history of immune disease, etc. Further investigations may include tests for acute hepatitis A, B, C, and E, imaging of the liver (e.g., biliary tract), autoantibodies, and cardiac ultrasound. If repeat testing confirms that the laboratory criteria in the table above are met, the possibility of potential drug-induced liver injury should be considered in the absence of other causes of liver function test abnormalities, without waiting for all liver function etiological tests to be made. Such cases of potential drug-induced liver injury should be reported as SAE.

7.1.1.2. Hypoglycaemic events

According to the classification criteria for hypoglycemia of the American Diabetes Association (ADA)/European Association of Diabetes (EASD) 2017 version, hypoglycemia is defined as follows:

- Warning level (Grade 1) of hypoglycaemia with a plasma glucose concentration of 70 mg/dl or less (3.9 mmol/L):

  Symptomatic hypoglycemia: a plasma glucose concentration of less than 70 mg per deciliter (3.9 mmol per liter) accompanied by symptoms associated with hypoglycemia.

  Asymptomatic hypoglycemia: a plasma glucose concentration of less than 70 mg per deciliter (3.9 mmol per liter) without symptoms associated with hypoglycemia.

  Unclassified hypoglycaemia: plasma glucose concentration no higher than 70 mg per deciliter (3.9 mmol per liter) with no documented information on hypoglycaemic symptoms.

- Clinically significant hypoglycemia (Grade 2) with a plasma glucose concentration of up to 54 mg/dl (3.0 mmol/L):

  Symptomatic hypoglycemia: a plasma glucose concentration of less than 54 mg per deciliter (3.0 mmol per liter) accompanied by symptoms associated with hypoglycemia.

  Asymptomatic hypoglycaemia: plasma glucose concentration not greater than 54 mg per deciliter (3.0 mmol per liter) without symptoms associated with hypoglycaemia.

  Unclassified hypoglycaemia: plasma glucose concentration no higher than 54 mg per deciliter (3.0 mmol per liter) with no documented information on hypoglycaemic symptoms.

- Severe hypoglycemia (Grade 3):

A behavior that requires assistance from another person to give carbohydrate, glucagon, or other aid in recovery. During this period, the patient develops a change in state of consciousness, is unable to take care of the aforementioned restorative treatment,

is unconscious or semi-unconscious, or becomes comatose (with or without seizure symptoms), and requires intravenous nutrition. During the onset of such symptoms, sometimes the plasma glucose concentration is undetectable at the time of symptom onset, but the symptoms are considered to be directly related to a decrease in blood glucose (plasma glucose concentration not higher than 70 mg per deciliter (3.9 mmol per liter) if neurological symptoms resolve after recovery of the glucose concentration.

Severe hypoglycaemia requiring medical attention: Severe hypoglycaemic events that require treatment by a healthcare provider (e.g., emergency care provider, emergency room staff).

Other Hypoglycaemic Events

- Relative hypoglycaemic events: symptoms associated with hypoglycaemia occur with a plasma glucose concentration above 70 mg per deciliter (3.9 mmol per liter), but it is expected that the plasma glucose level may rapidly approach the threshold of 70 mg per deciliter (3.9 mmol per liter).
- Possible symptomatic hypoglycaemic events: hypoglycaemic symptoms occur without a plasma glucose level measurement, but are thought to be associated with a decrease in the plasma glucose concentration not higher than 70 mg per deciliter (3.9 mmol per liter).

The investigator will determine whether a hypoglycemic event is severe based on the patient's need for medical assistance in addition to the expected assistance that is routinely received by the patient. All hypoglycaemic events will be recorded in the hypoglycaemic event module of the electronic event report form, and all severe hypoglycaemic events must be reported as serious adverse events.

During the study, if the subject experiences hypoglycemia symptoms at home, such as hunger, weakness, dizziness and headache, cold sweat in hands and feet, whole body shivering, coma, etc., the subject should contact the investigator in time and come to the hospital for examination as soon as possible according to the actual situation and give reasonable treatment.

7.1.1.3. Gastrointestinal reaction

Nausea, vomiting, and diarrhea are the primary gastrointestinal reactions of interest and will be recorded as AE in the eCRF. Each event was assessed for severity, duration (start and stop dates), and relationship to study drug or protocol procedures as deemed by the investigator. The severity of nausea, vomiting and diarrhea can be quantified by referring to the CTCAE grading in Table 7. However, the severity of AE in this study should be recorded as mild, moderate and severe according to the National Institute on Aging (NIA) criteria. It is recommended that CTCAE grade 1 be mild, CTCAE grade 2 be moderate, and CTCAE grade 3 be severe.

Table 7. Grading Criteria for Gastrointestinal Reactions

Gastrointestinal reaction	CTCAE Grade				
	Grade 1	Grade 2	Grade 3	Grade 4	Grade 5
Nausea	Decreased appetite without change in eating habits	Decreased oral food intake without significant weight loss, dehydration, or malnutrition	Inadequate intake of energy and water by mouth; Need for nasogastric feeding, total parenteral nutrition, or hospitalization	-	-
Vomiting	No intervention required	Outpatient intravenous fluid replacement; Medical intervention required	Need for nasogastric feeding, total parenteral nutrition, or hospitalization	Life Threatening	Death
Diarrhea	Increase in stool frequency < 4 times per day compared to baseline; Slightly increased stoma discharge	Increase in stool frequency from 4 to 6 times per day compared to baseline; Moderate increase in stoma discharge; Limitation of activities of daily living with the aid of instruments	Increase in stool frequency ≥ base per day compared to baseline; Requires hospitalization; Severe increase in stoma discharge compared to baseline; Limitation of self-care activities of daily living	Life-threatening; Need for emergency treatment	Death

7.1.1.4. Acute pancreatitis

Serum amylase and lipase will be monitored at the time points specified in the protocol, and additional tests may be added at the investigator's clinical discretion.

Blood amylase and/or lipase ≥ 3 times ULN, even if the patient does not have symptoms of acute pancreatitis, further diagnostic evaluation is required (refer to Appendix 6).

7.2. Definition of Serious Adverse Events

A serious adverse event is an adverse event that meets at least one of the following criteria:

- Results in death;
- Is life-threatening ("life-threatening" in the definition is an AE in which the subject was at risk of death at the time of its occurrence and does not include an AE that might have caused death if the event were to worsen);
- Requires inpatient hospitalization or prolongation of existing hospitalization, excluding the following:

- ✓ Rehabilitation facilities;
- ✓ Nursing home;
- ✓ Regular emergency room admissions;
- ✓ Same-day surgery (e.g. Outpatient/same-day/ambulatory surgery);
- ✓ Hospitalization or prolongation of hospitalization not associated with worsening of an AE is not per se an SAE. For example, hospital admission due to pre-existing disease, without occurrence of new adverse events or aggravation of pre-existing disease (e.g., to check for persistent laboratory abnormalities before the trial); Hospitalization for administrative reasons (e.g., routine annual physical examination); Hospitalization specified in the trial protocol during the clinical trial (e.g., operating according to the requirements of the trial protocol); Elective hospitalization (e.g., elective surgery) that is not associated with worsening of the adverse event; Scheduled treatments or surgeries should be recorded throughout the trial protocol and/or in the baseline data of the individual subject; Admitted for blood product use only.

- Results in permanent or significant disability/incapacity (significantly interfering with the ability to perform normal life functions).
- Resulting in a congenital anomaly/birth defect (offspring of a subject using the product).

Other important medical events: Events that may jeopardize the subject and may require medical or surgical intervention to prevent one of the outcomes listed above, although they do not result in death, are not life-threatening, or require hospitalization, are also considered serious based on appropriate medical judgment. Examples include drug-induced liver injury (Section 7.1.1.1) and severe hypoglycaemic events (Section 7.1.1.2).

7.3. Assessment of Severity of Adverse Events

The investigator will determine the severity according to the grading criteria for adverse events issued by the NIA.

- Mild: The symptoms or signs are perceptible, but easily tolerated and are minor irritants that do not affect normal activities, do not require treatment or medical identification, and are transient.
- Moderate: The event causes a low degree of inconvenience or concern to the patient and may interfere with daily activities, but usually improves with simple therapeutic measures; Moderate adverse events may cause some dysfunction.
- Severe: The event interrupts the patient's normal daily life and usually requires

systemic drug therapy or other treatment, often resulting in disability.

7.4. Causal relationship judgment between adverse event and study drug

For adverse events in clinical trials. The medically qualified investigator was required to provide an assessment of the causal relationship between the study drug and the adverse event. The elements in Appendix 7 were used to assess the causal relationship between the study drug and the adverse event.

7.5. Recording of Adverse Events

The investigator should use medical terminology/concepts to record AE or SAE. The use of spoken language and abbreviations should be avoided. All AE (including SAE) should be recorded on the Adverse Event Form of the eCRF.

7.5.1. Collection and timing of adverse events

Investigators were informed of adverse events by asking subjects non-inducing questions.

All AE, including SAE, whether observed by the investigator or spontaneously reported by the subject, were collected from the time of signing the informed consent through the end of the study.

Adverse Event of Special Interest (AESI) will be collected from the time a subject is randomized and takes the first dose of study drug until the end of the study.

7.5.2. Follow-up of adverse events

Adverse events should be followed up until they have returned to baseline or the investigator considers that no further follow-up is necessary for reasonable reasons (e.g., no recovery or improvement). If the adverse event cannot be recovered, a reasonable explanation should be recorded in the eCRF. The recovery of the subject's AE or SAE and its date should be recorded in the eCRF and medical records, whether or not related to the study drug.

7.5.3. Contents of Adverse Event Records

The investigator should fully record any adverse event, including diagnosis (if no diagnosis, record symptoms and signs including abnormal laboratory tests), start and stop dates and times (if applicable), change in severity, whether it is an SAE, whether it is an AESI, action taken with the study drug, treatment given due to the AE and the outcome of the event, and relationship of the adverse event to the study drug.

For SAE, the investigator should also provide the date the AE meets the criteria for an SAE, the date the investigator learns of the SAE, the rationale for the AE being an SAE, the hospitalization date, the discharge date, the probable cause of death, the date of death, whether an autopsy was performed, causality assessment with study procedures, causality assessment with other drugs, and other possible causes of the SAE. The

investigator should also provide the basis for the judgment of relatedness and the description of SAE. In the SAE description, the subject's number, age, gender, height and weight should also be included; Indications and disease stages of the subjects treated with the investigational drug and relevant systemic conditions; Occurrence, development, outcome and outcome of clinical course of SAE; Laboratory test results related to SAE (test time, unit and normal range must be provided); Previous history and concomitant diseases related to SAE as well as their occurrence and duration; Medication history related to SAE, concomitant drugs and their treatment initiation, duration, usage and dosage, etc.; Details of initiation, duration, and administration of study drug.

The items regarding AE recording are described below:

Diagnosis, symptoms and signs

If a diagnosis is already available, the diagnosis should be recorded on the eCRF rather than the individual signs and symptoms (e.g., liver failure should be recorded rather than jaundice, elevated transaminases, and asterixis). If signs and symptoms cannot be ascertained to be due to the diagnosis at the time of reporting, they will be recorded as a separate AE/SAE. If it is determined that the signs and symptoms are caused by the diagnosis, only the diagnosis is reported separately and the symptoms and signs are included in the diagnosis. AE needs to delete the record of symptoms and signs, and SAE needs to send a follow-up update report.

Adverse Events Secondary to Other Events

In general, adverse events secondary to other events (e.g., caused by other events or clinical sequelae) should be recorded as the primary event, unless the secondary event is serious or serious. However, secondary events with significant clinical significance should be recorded as separate adverse events in the eCRF if they occur at a different time from the primary event. If the relationship between the events is unclear, they should be recorded separately in the eCRF.

Persistent or Recurrent Adverse Events

A persistent adverse event is an adverse event that persists without resolution between the subject's two evaluation time points.

A recurrent adverse event is an adverse event that has resolved between the two evaluation time points but occurs later. The occurrence of the event should be recorded separately in the eCRF.

Laboratory test abnormality

Clinically significant laboratory abnormalities should be reported as AE. It is the responsibility of the investigator to review all laboratory abnormalities and to make medical judgment as to whether each laboratory abnormality should be reported as an AE.

Pre-existing medical condition

The existing symptoms/signs of subjects during the screening period should be recorded and reported as adverse events only when the severity, frequency and nature of the symptoms/signs are aggravated (except for the deterioration of the disease condition under study) after entering the trial. Changes from the previous state such as "increased headache frequency" should be reflected in the recording.

Overdose

A dose exceeding 20% of that specified in the protocol was considered an overdose. Overdoses will be recorded in the eCRF.

7.5.4. SAE Reporting

Reporting period of SAE: for serious adverse events that occur from signing of informed consent to the end of the study, if a subject/patient experiences an SAE, the investigator should immediately complete the Serious Adverse Event Report Form after being informed of the SAE, sign and date it, and immediately report it to the sponsor at drugsafety@innoventbio.com within 24 hours after being informed of the SAE.

For deaths and life-threatening serious adverse events, the investigator should urgently follow up on missing information and provide a complete SAE report.

SAE occurring outside the above period should also be reported to the sponsor if they are considered related to the study drug.

7.5.5. Pregnancy

Drugs of the same class may have safety risks of embryotoxicity. All subjects of childbearing potential participating in this clinical study must take effective contraceptive measures.

If a pregnancy occurs in a female subject exposed to the study drug during the clinical study, the investigator should report the pregnancy to the sponsor within 24 hours of becoming aware of the pregnancy and complete a Pregnancy Report Form.

If a male subject exposed to the study drug becomes pregnant during the clinical study, the subject may continue in the clinical study. The investigator should report the pregnancy to the sponsor within 24 hours of becoming aware of it and complete the Pregnancy Report Form.

The investigator should continuously monitor and follow up the pregnancy outcome until 8 weeks after delivery of the mother, and report the outcome to the sponsor.

If the outcome of the pregnancy is stillbirth, spontaneous abortion, fetal anomaly (any congenital anomaly/birth defect), and induced abortion for medical reasons, it is considered as an SAE and needs to be reported according to the procedures and timelines for SAE.

If a SAE during pregnancy was occured, a Serious Adverse Event/Adverse Event of

Special Interest Report Form should be completed and reported according to the SAE reporting procedure.

7.5.6. AESI Reporting

Events meeting the definition of an AESI were to be entered in the eCRF in a timely manner. If the criteria for SAE are met, please also report according to the requirements and timelines for SAE.

8. Statistical Considerations

To support the sponsor's communication with regulatory authorities regarding the IBI362 development strategy and Phase 3 study design, at least one interim analysis of efficacy and safety data will be conducted in this trial prior to the primary endpoint visit and a final analysis will be performed after subjects complete the trial and database lock.

The interim analysis will not affect the operation of the study. No formal statistical hypothesis testing will be performed. It will only be used to support the selection of dose for phase 3 trial. The subsequent study progress and study design will not be changed due to the results of the interim analysis.

8.1. Statistical Analysis Plan

A detailed Statistical Analysis Plan (SAP) will be written after the first subject is enrolled and finalized prior to database lock. The SAP will provide the full content and expression of the results of the analyses to be performed in this trial.

8.2. Hypothesis testing

Stage 1:

In this trial, the primary endpoint will be tested between each IBI362 dose group and placebo:

Mean percent change from baseline body weight after 24 weeks of IBI362 treatment: w1

Mean percentage change from baseline body weight in placebo group after 24 weeks: w0

H0: $w1=w0$

Ha: $w1 \neq w0$

The mean percent change in body weight was calculated separately for each treatment group and compared to placebo. Statistical superiority of either IBI362 dose group compared to placebo in reduction of body weight from baseline after 24 weeks of dosing was considered to be established if the difference from placebo was significant ($p < 0.05$).

No multiplicity adjustment will be made in this trial.

Stage 2:

In this trial, the primary endpoint will be tested between IBI362 and placebo in the 9.0 mg dose group:

Mean percent change from baseline body weight after 24 weeks of IBI362 treatment: w1

Mean percentage change from baseline body weight in placebo group after 24 weeks: w0

H0: w1=w0

Ha: w 1 ≠ w 0

Mean percent change in body weight will be calculated for IBI362 and compared to placebo. Statistical superiority of the 9.0 mg dose of IBI362 compared to placebo in reduction of body weight from baseline after 24 weeks of dosing will be considered if the difference between IBI362 and placebo was significant ($p < 0.05$).

8.3. Sample Size Estimation

For stage 1, assuming a weight loss of approximately 2.3% from baseline after 24 weeks in the placebo group, a weight loss of up to approximately 10% from baseline after 24 weeks in the IBI362 group, a standard deviation of weight loss of approximately 8%, a dropout rate of approximately 20%, an observed efficacy of 8%, and $\alpha = 0.05$ (two-sided), the sample size of approximately 80 subjects per dose group (approximately 240 subjects in total) will provid a power > 95%, that at least one of the doses of IBI362 would result in superior weight loss versus pooled placebo.

For stage 2, assuming a body weight loss of approximately 2.3% from baseline after 24 weeks in the placebo group and 12% from baseline after 24 weeks in the IBI362 group, with a standard deviation of approximately 8%, a dropout rate of approximately 20% and an observed efficacy of approximately 10%, $\alpha = 0.05$ (two-sided), a total of approximately 80 subjects will provide a power > 95%, that IBI362 9.0 mg would result in superior weight loss versus placebo.

8.4. Statistical Analysis Populations

Safety set (SS): Subjects who signed informed consent and took at least one dose of study drug.

Modified Intent-to-Treat Set (mITT): Subjects who were a subset of the Safety Set and had at least one post-baseline assessment.

PK concentration set: includes all subjects who received at least one dose of the study drug and had at least one valid concentration data of the tested components after administration.

PD analysis set: including all subjects who received at least one dose of study drug

and had at least one valid test result at baseline and after drug administration.

Anti-drug antibody analysis set: includes all subjects who received at least one dose of study drug and had at least one valid test result.

8.5. Statistical Analysis Methods

8.5.1. General Methods of Statistical Analysis

Measurement data are described with mean, standard deviation, median, maximum and minimum; Count data were described by frequency and percentage. Data will be analyzed separately for subjects within each IBI362 dose group and for pooled placebo subjects unless otherwise specified.

All statistical analyses were performed using SAS9.4 (or higher).

8.5.2. Evaluation index

- **Efficacy indicators:**

The primary and secondary efficacy endpoints include: changes in body weight, BMI, waist circumference, HbA1c, fasting blood glucose, systolic blood pressure, diastolic blood pressure and blood lipids (TC, TG, LDL-C and HDL-C) after 24 weeks of treatment and after 12 weeks of discontinuation after 24 weeks of administration. Change in body fat (total body fat, abdominal fat, liver fat) after 24 weeks of administration.

Exploratory efficacy variables include changes in high-sensitivity C-reactive protein (hsCRP), proprotein convertase subtilisin/kexin type 9 (PCSK9), fibroblast growth factor-21 (FGF-21) and adiponectin after 24 weeks of treatment. Changes of serum uric acid and glutamic pyruvic transaminase after 12 and 24 weeks of adminstration.

- **Safety indicators:**

Incidence, relatedness to study drug and severity of all AE, TEAE, Treatment-related Adverse Event (TRAE), Immune-related Adverse Event (irAE), hypoglycemic events and SAE.

Changes in vital signs, physical examination, and laboratory results before, during, and after study treatment.

- **Immunogenicity Evaluation:**

Immunogenicity evaluation was completed based on IBI362 ADA and NAb assay results.

8.5.3. Efficacy Analysis

Efficacy analyses were performed in the modified intent-to-treat analysis set.

The primary efficacy endpoint of this trial is the percentage change from baseline in body weight after 24 weeks of injection.

An ANCOVA model with baseline body weight as a covariate will be used to estimate the percent change from baseline in mean body weight for each IBI362 dose group compared to placebo, the difference between groups, and its 95% confidence interval.

For other continuous efficacy endpoints, mean ± standard deviation, maximum, minimum, and median will be summarized by group, and p-values will be calculated for each IBI362 group versus placebo using a two-sample t-test, with point estimates and 95% confidence intervals for the corresponding differences.

For categorical endpoints, rates within each group will be calculated and 95% confidence intervals will be calculated using Clopper-Pearson, and the chi-square test will be used to compare the difference between IBI362 and placebo groups and the corresponding 95% confidence intervals will be calculated. The odds ratio and its 95% confidence interval for each IBI362 dose group versus placebo will also be estimated for the proportion of subjects with body weight changes from baseline ≥ 5. 0% and ≥ 10. 0% using a logistic model including baseline body weight as a covariate.

8.5.4. Safety Analysis

Safety analyses were performed in the Safety Analysis Set.

The number of subjects with each AE was summarized by Medical Dictionary for Regulatory Activities (MedDRA) system organ class, preferred term, and adverse event grade, the number and percentage of subjects with each category of AE (including causality, severity, SAE, etc.) were summarized, and each category of AE was further summarized by MedDRA system organ class and preferred term.

8.5.4.1. Drug Exposure

Study drug exposure, duration of administration (weeks), relative dose intensity, etc. were summarized for subjects during the study.

8.5.4.2. Adverse Events

The number of subjects with each AE was summarized by MedDRA system organ class, preferred term, and adverse event grade, and the number and percentage of subjects with each category of AE (including causality, severity, AESIs, SAE, etc.) were summarized, and each category of AE was further summarized by MedDRA system organ class and preferred term.

8.5.4.3. Laboratory Tests

Measured values and changes of hematology, blood biochemistry, physical condition, vital signs and other indicators before and after administration will be described using mean ± standard deviation, maximum, minimum and median by dose group, respectively. Cross classification table will be used to describe normal and abnormal changes before and after administration.

8.5.4.4. 12-lead ECG examination

Describe ECG measurements and changes with treatment. The changes between normal and abnormal before and after treatment will be described using cross-categorical tables.

8.5.4.5. Vital Signs, Physical Examinations, and Other Safety-Related Tests

Descriptive statistics will be provided for changes in vital signs of subjects. Describe the proportion of "abnormal and clinically significant" among subjects with abnormal changes, where the abnormality is clinically significant or not as judged by the investigator.

Urinalysis: cross classification table will be used to describe the changes of normal and abnormal before and after treatment.

8.5.4.6. Immunogenicity

The occurrence of ADA and NAb to IBI362 at all follow-up visits will be summarized separately for each dose group.

8.5.4.7. Other safety variables

Describe the measured value and change value of each measurement point of ECG. Cross classification table will be used to describe the normal and abnormal changes before and after administration in each dose group. Hypoglycemic events and injection site reactions were summarized by treatment group.

8.5.5. Pharmacokinetic Analysis

The population pharmacokinetic profile of IBI362 will be analyzed using nonlinear mixed effects modeling.

8.5.6. Pharmacodynamic Analysis

Measured values and changes from baseline for each PD parameter at baseline and at each time point after dosing were summarized separately for each treatment group and analyzed with descriptive statistics (mean ± standard deviation, maximum, minimum, median). PD parameters were: fasting plasma glucose, fasting insulin.

8.5.7. Planned Interim Analysis

In order to support the sponsor's communication with regulatory authorities, such as the IBI362 development strategy and the Phase 3 clinical study design, at least one interim analysis of efficacy and safety data will be conducted in this study before the primary endpoint visit. The results of the interim analysis will not be disclosed to the personnel involved in the operation of this study, and will not affect the operation of the trial and the subsequent study progress. The results of the interim analysis will only be used to support the dose recommendation for phase 3 trial, and the clinical study design will not be changed due to the results of the interim analysis.

The interim analysis will be completed by an unblinded sponsor team not directly involved in the trial, and the sponsor unblinded team will be identified prior to the start of the first interim analysis. The unblinded team will consist of a medical monitor, a statistician, and a programmer. Before the first interim analysis, the blinded project statistician and programmer of this trial will develop the statistical analysis plan, prepare the interim analysis procedure based on the blinded data, and submit the procedure and the statistical analysis plan to the statistician of the unblinded team. At the time of interim analysis, the unblinded drug administrator will transmit the blind code information of the randomized subjects to the statistician of the unblinded team before the analysis, and the statistician of the unblinded team will generate the interim analysis report according to the blind code and the pre-prepared procedure. Additional analyses of safety and/or efficacy will be performed if necessary. The unblinded team will submit the efficacy and safety results summarized at the treatment group level to the designated unblinded personnel of the sponsor for the design of the phase 3 trial and communication with regulatory authorities. Except for the sponsor's unblinded team and designated sponsor's unblinded personnel, the sponsor's members not in direct contact with subjects and investigators will be unblinded after all subjects have completed the week 25 primary endpoint visit and the primary endpoint database lock, and the remaining sponsor's personnel will remain blinded during this until the database is finally locked after all subjects have completed the week 36 discontinuation visit.

8.5.7.1. Independent Data Monitoring Committee (IDMC)

Not applicable.

8.5.8. Subgroup Analyses

In the first stage of the study, subgroup analyses will be performed for the stratification factor baseline BMI (24.0 to 28.0 kg/m^2 versus BMI $\geq$ 28. 0 kg/m^2). Other subgroup analyses will be defined and described in detail in the SAP.

8.5.9. Adjustment for Multiple Comparisons and Multiplicity

No adjustment for multiplicity was considered in this trial.

8.5.10. Analysis of Exploratory Endpoints

Exploratory indicators: high-sensitivity C-reactive protein (hsCRP), proprotein convertase subtilisin/kexin type 9 (PCSK9), fibroblast growth factor-21 (FGF-21) and adiponectin levels and their changes from baseline will be summarized and described in each treatment group at baseline and after 24 weeks of treatment.

Total fat content, local visceral fat content, total lean body mass as measured by dual-energy X-ray absorptiometry (DEXA), and intra-abdominal fat area (VFA), subcutaneous fat area (SFA), and total intra-abdominal fat area (TFA) as measured by MRI were summarized by treatment group at each visit, and changes from baseline after

24 weeks of dosing were compared.

Liver fat content as measured by MRI-PDFF will be summarized by treatment group at each visit and change from baseline after 24 weeks of dosing.

8.6. Control of bias

8.6.1. Randomization and blinding

Subjects will be randomly assigned in a 3: 1 ratio to IBI362 and placebo in each dose group using a block randomization method.

Subject randomization will be completed by the IWRS. Successfully randomized subjects will be given a randomization number and will receive drug treatment according to the drug number assigned by the system. The unblinded statistician will complete the blinding of the subject's randomization number and medication number, which will be transferred to the unblinded administrator of the randomization system to complete the preparation and management of IWRS blinding.

A central randomization method was used, with competing enrollments from each center. A central randomization system will be used for the central randomization procedure. If the subject completes all screening assessments of the study and meets the enrollment criteria, he/she will receive a randomization number generated by IWRS, which will connect the subject to the assigned treatment group and can be assigned to dispense the investigational product according to the amount required. The randomization number of a randomized subject will be retained regardless of withdrawal from the study for any reason. IWRS personnel will only be responsible for the configuration and management of the randomization system and will not be involved in any specific trial operations.

8.6.2. Assessment of Blinding Maintenance

The subjects in the two stages of this study were enrolled and analyzed separately, and the operation and analysis of the two stages of the study were not affected by each other. The two stages of the study will be unblinded separately.

Take the first stage as an example. All subjects in stage 1 and the investigators, monitors, sponsor personnel, and representatives involved in their treatment or clinical evaluation remained blinded until all subjects in stage 1 completed their week 25 visit. After all subjects in stage 1 have completed the week 25 visit, the subjects in stage 1 and all investigators, monitors and sponsor personnel in contact with the investigators and subjects who participate in the treatment or clinical evaluation will continue to remain blinded until the end of stage 1, and other sponsor personnel will not remain blinded.

The second stage of blinding maintenance as above.

8.6.3. Unblinding and Emergency Unblinding

Unblinding of subjects will be performed in each of the two stages. After all subjects in each stage dosing for 24 weeks, the sponsor will be unblinded after completion of the week 25 visit and after the primary endpoint database for these subjects is locked. Investigators and subjects will remain blinded until the end of this stage of the study, i.e., after the end of the first stage, all investigators, subjects in the first phase and sponsor personnel will be unblinded; All investigators, stage 2 subjects, and sponsor personnel will be unblinded at the end of the stage 2 study.

In the clinical study, the investigator may unblind the subject in case of emergency medical events due to the safety of the subject when the investigator needs to know the investigational drug used by the subject. Emergency unblinding must be performed in the system by authorized personnel designated by the site as per procedure. Before unblinding of the investigational drug, relevant personnel of the sponsor should be notified. After obtaining the approval from the principal investigator and the sponsor, the subject should be entered into the IWRS system for emergency unblinding so as to obtain the specific grouping information of the subjects. The investigator should record the time, location and reason for unblinding (the grouping information after unblinding should not be recorded on the eCRF).

9. Study Quality Assurance and Quality Control

In accordance with the guidelines of Good Clinical Practice (GCP), the sponsor is responsible for implementing and maintaining a quality assurance and quality control system according to corresponding standard operating procedures to ensure that the conduct of the clinical trial and the collection, recording and reporting of data comply with the protocol, GCP and applicable regulatory requirements.

9.1. Clinical Monitoring

Clinical monitoring of this study was performed by the Sponsor. CRAs should perform monitoring in accordance with the sponsor's standard operating procedures and have the same rights and responsibilities as the sponsor's CRAs. The monitor should maintain regular communication with the investigator and the sponsor.

Prior to the start of the study, the monitor will assess the competence of each study site and report relevant problems with facilities, technical equipment, or medical personnel to the sponsor. During the study, the monitor will be responsible for monitoring whether the investigator has obtained written informed consent from all subjects and whether the data records are correct and complete. At the same time, the monitor will also compare the data entered into the eCRF with the original data and inform the investigator of any errors or omissions. The monitor will also supervise the study site for protocol compliance, arrange for the supply of study drug, and ensure that the drug is stored under appropriate conditions.

Monitoring visits will be conducted as required by applicable laws and regulations. Beginning with subject enrollment, each site will undergo regular monitoring visits. After each visit to the investigator, the monitor should submit a written report to the sponsor.

9.2. Data Management

Electronic Data Capture (EDC) system will be used in this study, and study data will be entered into the eCRF by the investigator or authorized study personnel. Prior to site initiation or data entry, the investigator and authorized study personnel will be appropriately trained and appropriate security measures will be taken for the computers and other equipment used.

Data entry into the eCRF should be completed as soon as possible during or after the visit and updated at any time to ensure that it reflects the latest developments of the subjects participating in the study. To avoid differences in the assessment of results by different evaluators, it is recommended that baseline and all subsequent efficacy and safety assessments for the same subject be performed by the same person. The investigator was required to review the data to ensure the accuracy and validity of all data entered into the eCRF. If certain assessments are not performed during the course of the study, or certain information is not available, not applicable, or unknown, the investigator should record it in the eCRF. The investigator should electronically sign the data after verification.

The Clinical Research Associate (CRA) will review the eCRFs against the source documents and assess their completeness and consistency, and the CRA will compare the eCRFs with the source documents to ensure the consistency of key data. All data entries, corrections, and modifications will be the responsibility of the Investigator or his/her designee. Data from the eCRF will be submitted to the EDC database and any changes to the data will be recorded in the audit trail, i.e. The reason for the modification, operator username, date and time of the modification will be recorded. The roles and permissions of the site personnel responsible for data entry will be pre-determined. In case of any data query, CRA or data management personnel will issue the query in EDC, and relevant personnel of the study site will be responsible for answering the query. The EDC system will record the audit trail of queries, including user name, time, and date.

Unless otherwise specified, the eCRF will only be used as a form for data collection and will not be used as source data. Source documents are all records used by the investigator or the hospital, related to the subject and capable of proving the existence of the subject, inclusion and exclusion criteria and their participation in this study, including laboratory records, ECG results, medication records and subject folders.

The investigator is responsible for maintaining all source documents and for monitoring them by the CRA at each visit. In addition, the investigator was required to submit a completed eCRF for each enrolled subject, regardless of the duration of the

enrolled subject's participation in the study. All supporting documents (e.g., laboratory or hospital records) submitted with the eCRF should be carefully verified for the protocol number and subject number, and all personal privacy information (including subject name) should be deleted or illegible to protect subject privacy. The investigator certifies by electronic signature that he/she has reviewed all eCRF data to ensure the validity, completeness, and accuracy of the data. The electronic signature will be completed using the user ID and password of the investigator. The date and time of the signature will be automatically attached by the system. The investigator may not share the user ID and password with other personnel. Changes to data in the eCRF should be made according to the workflow defined in the EDC system. All changes and reasons for changes will be documented in the audit trail.

9.3. Quality Assurance Audit

Quality assurance audits of the study site, study database, and associated study documents may be conducted by the sponsor or authorized representatives of the sponsor during the course of the study, and inspections of the study site, study database, and associated study documents may also be conducted at the discretion of the appropriate regulatory authorities. When notified of an inspection by a regulatory authority, the investigator will notify the sponsor immediately.

Site audits were conducted by the sponsor's Quality Assurance Unit. Audits included: drug supplies, required trial documents, records of the informed consent process, and consistency of the case report forms with source documents. Audit content and scope may also be added as appropriate. After reasonable notification, the investigator should allow auditors entrusted by the sponsor to conduct trial-related audits and inspections by regulatory authorities. The main purpose of the audit or inspection is to verify that the rights or health of the subjects participating in the trial are protected, that the informed consent is signed and the trial process is properly conducted, and that all data related to the evaluation of the study drug are handled and reported in accordance with the pre-planned arrangement, protocol, facilities, ethical Standard Operating Procedure, GCP and applicable regulatory requirements. The investigator should have direct access to all trial documents, original records and raw data.

10. Ethics

10.1. Ethics Committee

The sponsor or its authorized representative of the sponsor will prepare relevant documents to be submitted to the Ethics Committee (EC) of the study site, including the trial protocol, informed consent form, investigator's brochure, subject recruitment materials or advertisements and other documents required by laws and regulations, which shall be submitted to the corresponding EC for review and approval. Written approval from the EC must be obtained and provided to the Sponsor prior to initiation of the study.

The EC approval letter must clearly describe the name, number and version number of the study protocol and the version number of other documents (such as informed consent form) and approval date. The Investigator was required to notify the Sponsor of the EC's written comments on the delay, suspension, and re-approval.

The site must comply with the requirements of the site's EC. May include protocol amendments, informed consent form amendments and subject recruitment materials amendments to be submitted to EC for review and approval, local safety reporting requirements, periodic reports and updates according to EC regulations, and final report submission. All of the above documents and EC approvals must be provided to the Sponsor or its designee.

10.2. Ethical conduct in the study

The study process and informed consent shall comply with the Declaration of Helsinki, relevant GCP requirements and relevant laws and regulations of China concerning drug and data protection.

GCP provides ethical, scientific and global quality standards for the design, conduct, recording, and reporting of clinical studies involving human subjects. This study will be conducted in accordance with GCP and relevant national regulations and in accordance with the relevant ethical principles in the Declaration of Helsinki to protect the rights, safety and well-being of the subjects.

The investigator is required to comply with the procedures specified in this trial protocol and shall not make changes without the permission of the sponsor. Any protocol deviations will be reported to the EC, the Sponsor, or regulatory authorities.

10.3. Subject Information and Informed Consent

Prior to any study procedures, the possible risks and benefits of the study will be explained to potential subjects using an informed consent form (ICF) that will be easily understood. The ICF statement should specify that the informed consent is voluntary and the possible risks and benefits of participating in the study should be specified, and the subject may withdraw from the study at any time. The investigator can only enroll a subject after fully explaining the details of the study, satisfactorily answering the subject's questions and giving sufficient time for consideration, and obtaining the written consent of the subject or his/her legal representative. All signed informed consent forms must be in the investigator's file or in the subject's folder.

The investigator is responsible for explaining the content of the informed consent to the subject and obtaining the informed consent form signed and dated by the subject or his/her legally acceptable representative prior to the start of the study. After signing, the investigator should send a signed informed consent form to the subject. The investigator should record the informed consent process in the trial source documents.

The initial informed consent form, any subsequent amendments to the written informed consent form, and any written information provided to subjects should be subject to IRB/IEC opinion prior to use. If new information becomes available that may be relevant to the subject's willingness to continue participation in the trial, the subject or his/her legally acceptable representative should be informed in a timely manner. Communication of this information will be provided and documented via a revised informed consent form or an addendum to the original informed consent form (obtaining the subject's dated signature or the subject's legally acceptable representative's dated signature).

10.4. Data Protection

Information on data protection and privacy will be included in the ICF (or, in some cases, along with the use of separate documents).

Precautions were taken to ensure the confidentiality of documents and to prevent identification of subjects. However, under special circumstances, some individuals may see genetic data and personal identification codes for a subject. For example, in the event of a medical emergency, the sponsor, its representative physician, or investigator will be aware of the subject identification code and have access to the subject's genetic data. In addition, access to relevant documents is required by the relevant regulatory authorities.

10.5. Protocol Violation

A protocol violation was defined as any non-compliance with the clinical study protocol, International Conference on Harmonisation Good Clinical Practice (ICH GCP), or Manual of Operations (MOP) requirements. Non-compliance may come from the subject, investigator, or study site staff. In response to the violation, corrective action shall be taken and completed in a timely manner.

11. Study Management

11.1. Data Handling and Record Retention

The documents in the clinical trial (protocol and protocol amendment, completed eCRF, signed ICF, etc.) should be kept and managed in accordance with the requirements of GCP. The site should retain these documents for 5 years after the end of the study.

Study documents should be properly retained for future access or data traceability. Safety and environmental risks should be considered when preserving documents.

No study documents will be destroyed without the written permission of the Sponsor and the Investigator. Only after notifying and obtaining written consent from the Sponsor, the Investigator/study site may transfer the study documents to other parties who comply with the document retention requirements or to other locations where they meet the requirements.

11.2. Access to Raw Data/Documents

The investigator agrees that the sponsor and relevant authorized regulatory authorities have direct access to all study-related documents, including the subject's medical records.

11.3. Protocol Amendment

All amendments to the protocol made during the course of the study were to be communicated and agreed upon by the sponsor and the investigator. The sponsor should ensure that protocol amendments are submitted to regulatory authorities in a timely manner.

All amendments to the protocol will be retained as protocol supplements. Any amendment to the protocol should be submitted to the Ethics Committee for approval or filing according to the provisions of the Ethics Committee. If required, it should also be submitted to regulatory authorities for approval and, if required, approved by the EC and regulatory authorities before implementation (except for changes to the protocol to eliminate an immediate hazard to trial subjects).

11.4. Investigator Responsibilities

The investigator will carry out this study in accordance with the protocol, ethical principles in the Declaration of Helsinki, China GCP and relevant laws and regulations.

The detailed responsibilities of the relevant investigators are listed in Chapter 5 of the China GCP (2020 No.57).

11.5. Publication Policy

All data generated in this study are the confidential information of the Sponsor and the Sponsor has the right to publish the results of the study. Information on the publishing policies of the sponsor and investigators will be described in the clinical trial agreement.

All information related to this trial (not limited to the following documents: protocol and investigator's brochure) must be strictly confidential. The investigator must be aware that the scientific or medical conclusions drawn from this trial may be of commercial value to the sponsor. The investigator should keep the information and data related to this trial confidential. If the data related to this trial or the conclusions drawn from the trial are to be published publicly, the investigator should negotiate with the sponsor in advance and obtain the written consent of the sponsor. In order to protect their own rights and interests, the sponsor may require the investigator not to publish relevant trial data before the investigational product is approved for marketing.

The sponsor has the right to publish or publish information or data related to this trial or to report it to the drug regulatory authorities. If the sponsor needs to include the name of the investigator in the publication, publication or advertisement, the sponsor should obtain the consent of the investigator.

11.6. Finance and Insurance

The sponsor will purchase insurance for subjects participating in this study in accordance with local regulations and minimum requirements. The terms of the insurance will be kept in the study binder.

12. References

[1]. Pocai A. Action and therapeutic potential of oxyntomodulin. Mol Metab. 2014; 3 (3): 241-51.

[2]. Tan TM, Field BC, McCullough KA, et al. Coadministration of glucagon-like peptide-1 during glucagon infusion in humans results in increased energy extension and ameliation of hyperglycemia. Diabetes. 2013; 62 (4): 1131-8.

[3]. Obesity Group, Chinese Society of Endocrinology, Chinese Medical Association. Expert consensus on prevention and treatment of adult obesity in China. Chinese Journal of Endocrinology and Metabolism. 2011; 27 (9): 711-717.

[4]. Marie Ng, Tom Fleming, et al. Global, Regional, and National Prevalence of Overweight and Obesity in Children and Adults During 1980-2013: A Systematic Analysis for the Global Burden of Disease Study 2013. Lancet. 2014; 384 (9945): 766-81.

[5]. Https://www.cdc.gov/obesity/data/index.html

[6]. Snow V, Barry P, Fitterman N, et al. Pharmacological and surgical management of obesityin primary care: a clinical practice guideline from the American College of Physicians. Ann Intern Med. 2005; 142: 525-31.

[7]. George A Bray, et al. Management of Obesity. Lancet. 2016; 387 (10031): 1947-56.

[8]. Knowler WC, Barrett-Connor E, Fowler SE, Hamman RF, Lachin JM, Walker EA, et al. Reduction in the incidence of type 2 diabetes with lifestyle intervention or metformin. N Engl J Med. 2002; 346 (6): 393-403.

[9]. Dattilo AM, Kris-Etherton PM. Effects of weight reduction on blood lipids and lipoproteins: a meta-analysis. Am J Clin Nutr. 1992; 56 (2): 320-328.

[10]. Campbell JE, Drucker DJ. Islet α cells and glucagon--critical regulators of energy homeostasis. Nature ReviewsEndocrinology. 2015; 11 (6): 329-338.

[11]. Baggio LL, Huang Q, Brown TJ, Drucker DJ. Oxyntomodulin and glucagon-like peptide-1 differentially regulated murine food intuition and energy expendability. Gastroenterology. 2004; 127 (2): 546-58.

[12]. Pocai A, Carrington PE, Adams JR, Wright M, Eiermann G, Zhu L, et al. Glucagon-like peptide 1/glucagon receptor dual agonism reverses obesity in mice. Diabetes. 2009; 58 (10): 2258-66.

[13]. Pocai A. Unraveling Oxyntomodulin, GLP1's enigmatic brother. J Endocrinol. 2012; 15: 335-346.

[14]. Day JW, Ottaway N, Patterson JT, et al. A new glucagon and GLP-1co-agonist eliminates obesity in rodentis.Nat Chem Biol. 2009; 5: 749-757.

[15]. Kosinski JR, Huber J, Carrington PE, et al. The glucagon receptor is evolved in mediating the body weight-lowering effects of oxyntomodulin. Obesity. 2012; 20: 1566-1571.

[16]. Lao J, Hansen BC, DiMarchi R, et al. Effect of GLP1R/GCGR dual agonist in monkeys. Diabetes. 2013; 62 (suppl 1): A257.

[17]. Ralf Elvert, Andreas W. Herling. Running on mixed fuel-dual agonistic approach of GLP-1 and GCG receptors leads to beneficial impact on body weight and blood glucose control: A comparative study between mice and non-human primate. Diabetes Obes Metab. 2018; 20: 1836-1851.

[18]. Jorgensen R, Kubale V, Vrecl M, Schwartz TW, Christian E Elling. Oxyntomodulin differential affects glucagon-like peptide-1 receptor β-arrestin receptor and signaling through Ga (s). Journal of Pharmacology and Experimental Therapeutics. 2007; 322: 148-154.

13. Appendices

Appendix 1 Protocol Amendment History

Table 1. Protocol Amendment History

Version	Date
V1.0	21 Dec 2020
V2.0	14 May 2021
V3.0	22 Nov 2021
V4.0	21 Mar 2022
V4.1	02 Apr 2022
V5.0	24 Aug 2022

Appendix 2 Dyslipidemia Reference Standards

Refer to the Guidelines for Prevention and Treatment of Dyslipidemia in Chinese Adults (2016 Revision).

Table 1. Appropriate level and abnormal stratification standard of blood lipids in primary prevention population of ASCVD in China [mmol/L (mg/dl)]

Stratification	**TC**	**LDL-C**	**HDL-C**	**Non-HDL-C**	**TG**
Ideal level		< 2.6 (100)		< 3.4 (130)	
Appropriate level	< 5.2 (200)	< 3.4 (130)		< 4.1 (160)	< 1.7 (150)
Increase near the edge	⩾5.2 (200) and < 6.2 (240)	⩾3.4 (130) and < 4.1 (160)		⩾ 4.1 (160) and <4.9 (190)	⩾ 1.7 (150) and <2.3 (200)
Increase	⩾6.2 (240)	⩾4.1 (160)		⩾4.9 (190)	⩾2.3 (200)
Reduce			< 1.4 (40)		

ASCVD: atherosclerotic cardiovascular disease; TC: total cholesterol;LDL-C:low-density lipoprotein cholesterol; HDL-C: high-density lipoprotein cholesterol;non-HDL-C:non high-density lipoprotein cholesterol;TG:triglycerides.

Tabel 2. Clinical classification of dyslipidemia

	**TC**	**LDL-C**	**HDL-C**	**WHO PHENOTYPE**
Hypercholesterolemia	increase			II a
Hypertriglyceridemia		increase		IV, I
Mixed hyperlipidemia	increase	increase		II b, III, IV, V
Low-HDL cholesterol			reduce	

TC: total cholesterol; HDL-C: high-density lipoprotein cholesterol; TG:triglycerides; WHO: World Health Organization.

Appendix 3 CKD-EPI formula (eGFR estimation formula)

Estimated by CKD-EPI formula: eGFR = a × [(serum creatinine (mg/dl)/b)] ^{c} × (0.993) ^{age};

Sex	A Value	B-value	C-value	
			Serum creatinine ≤ 0. 7mg/dl	Serum creatinine > 0.7 mg/dl
Female	144	0.7	-0.329	-1.209
Male	141	0.9	-0.411	-1.209

Appendix 4 Calculation formula of QTcF

QTc Fridericia formula: QTcF = QT/(RR ^ 0.33)

Appendix 5 Oral Glucose Tolerance Test (OGTT) Methods

1. Beginning at 7-9 a.m., subjects will take 75 g of anhydrous glucose powder dissolved in 300 mL of water orally after fasting (8-10 h), or 82.5 g if 1 molecule of water glucose is used. Children are given 1.75 g per kilogram of body weight, not to exceed 75 g. Sugar water will be taken within 5 minutes.
2. Blood samples will be taken from forearm before and 2 hours after taking glucose from the first mouth of glucose.
3. During the course of the study, the subjects can not drink tea or coffee, can not smoke, can not do strenuous exercise, but are not absolutely bed-ridden.
4. Blood samples should be submitted as soon as possible.
5. Within 3 days before the test, the daily carbohydrate intake shall not be less than 150 g.
6. The drugs that may affect OGTT, such as contraceptives, diuretics or phenytoin sodium, will be stopped for 3 ~ 7 days before the test.

Appendix 6 Pancreatic enzyme monitoring and management procedures for asymptomatic patients with pancreatitis[1, 2]

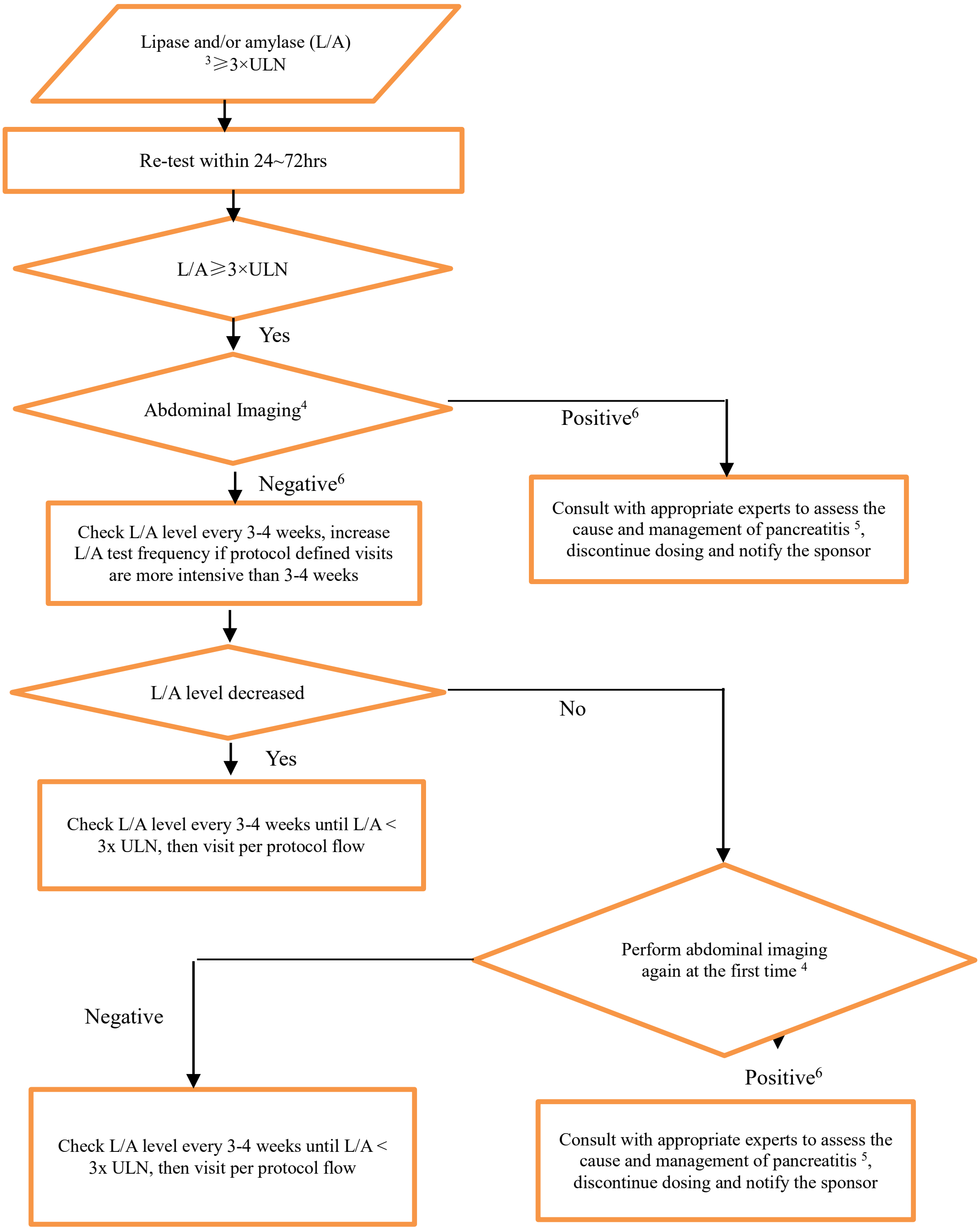

1. Symptomatic mainly refers to abdominal pain associated with pancreatitis, and severe nausea, vomiting and other symptoms can also be considered relevant by the investigator.
2. At any time, if the investigator believes that a subject has symptoms of acute pancreatitis, whether or not based on the L/A test results, he/she should consult a specialist for evaluation, management, assess the cause of pancreatitis, stop dosing,

and notify the sponsor.

3. Either or both of serum lipase and serum amylase can be used as evaluation criteria.
4. The best time for abdominal imaging is when pancreatic enzymes are elevated. If judged safe by the investigator or radiologist, contrast-enhanced abdominal CT is preferred, and MRI is acceptable.
5. At a minimum, liver function tests, triglycerides, and blood calcium levels will be checked, and all concomitant medications will be recorded.
6. Negative or positive imaging results specifically indicate acute pancreatitis.

Appendix 7 List of Causal Relationship between Adverse Events and Study Drug

**Relationship to Sponsor Product**	Was the study drug responsible for the adverse event? The medically qualified investigator is required to provide a causal assessment of the relationship between the study drug and the adverse event. The investigator will sign/date (initials) the source document or worksheet to support the causality assessment on the AE form to ensure a medically qualified causality assessment. This signed document must be retained for the required regulatory timeframe. The following criteria are intended to serve as a reference guide to assist the investigator in assessing the relationship between the investigational product and the occurrence of an adverse event based on the available information.<br>**The following elements were used to assess the relationship between the study drug and the AE; The greater the correlation (in terms of number and/or intensity) between the items and their corresponding elements, the greater the likelihood that the study drug will cause an adverse event;**	
	**Exposure**	Is there evidence that the subject is indeed exposed to the trial drug, e.g., a true and credible past medical history, acceptable compliance assessments (drug counts, logs, etc.), expected pharmacological effects, measurement of drug/metabolites in in vivo collected specimens?
	**Time course**	Is there a reasonable temporal sequence between the adverse event and treatment with the study drug?<br>Did the adverse event occur at a time consistent with a drug-induced adverse event?
	**Reason other than study drug**	Is there an alternative etiology for the adverse event, such as underlying disease, other drugs/vaccines, or other host or environmental factors
	**Dechallenge**	Was study drug discontinued or dose/exposure/frequency reduced?<br>If yes, did the AE resolve or improve?<br>If yes, a positive dechallenge is indicated. If not, a negative dechallenge is indicated.<br>Note: This criterion does not apply if: (1) an adverse event results in death or permanent disability; (2) AE recovered/improved despite continued use of study drug; (3) The trial was a single-dose trial of the drug; (4) Only one dose of the study drug.

	**Rechallenge**	Has the subject been repeatedly exposed to the study drug in this trial?<br>If yes, did the AE resolve or improve?<br>If yes, a positive rechallenge. If not, the rechallenge test was negative.<br>Note: This criterion does not apply if: (1) the initial AE resulted in death or permanent disability, (2) the trial was a single-dose clinical trial, and (3) only one dose of study drug was administered.<br>Note: If a rechallenge is planned for an adverse event that is serious and possibly attributable to the investigational product, or if reexposure to the investigational product may pose a serious potential risk to the subject/patient, rechallenge is not recommended, unless it is considered that continuation of the investigational product may be beneficial to the patient and no alternative treatment is available, and may be conducted after prior approval by the sponsor.
	**Consistency with trial treatment characteristics**	Is the clinical/pathological presentation of the adverse event consistent with previous data on the investigational drug or pharmacology and toxicology studies of such drugs?
The medically qualified investigator will record the causality assessment based on his/her best clinical judgment, including consideration of the above causality factors. The causality assessment of the adverse event to the study drug will be recorded as "related" and "not related".		
**Record Causality**		**The table below can be used for causality assessment (not all criteria need to be met)**
**Related**		There was generally evidence of exposure to study drug. An AE with a reasonable temporal sequence from administration of the sponsor product. The occurrence of an AE is more likely to be explained by the study drug than by other causes.
**Not related**		Generally, it means that the subject does not take the study drug, the time between the occurrence of adverse event and the occurrence of adverse event is unreasonable or there are other reasons that can better explain the adverse event, but not the study drug.

Appendix 8 SOP for Fasting Body Weight Measurement

For each subject, weight measurements should be performed in a uniform manner at each clinic visit using a calibrated scale (either mechanical or electronic). Each weight measurement of the subject should be performed on the same scale after the subject has emptied his bladder. For weight measurement, subjects should take off weight-added clothing such as coat/pants/hat/scarf/necklace/waistband, only single clothes (one top and one undercoat limited), and shoes. Ensure that the scale is zeroed prior to weighing.

a) Make sure the scale is placed on a firm and smooth surface before weighing (do not place on carpets or sloping surfaces or rough surfaces).
b) Before weighing, the symmetry area of the curtain should be pulled to isolate, so as to protect the privacy of the subject when changing clothing.
c) Ensure that the subject does not feel cold after undressing prior to weighing.
d) After verifying whether the subject has voided, guide the subject into the weighing area, pull the curtain, and ask the subject to take off the clothes and clothing for weight increase such as coat/pants/hat/scarf/necklace/belt, and only wear single clothes (one top and one undercoat limited) and take off the shoes.
e) Subjects will be asked to step on the scale with their feet on each side of the scale.
f) Subjects will be asked to stand still with their arms on their sides, and their weight was recorded in kilograms (kg).

Appendix 9 Waist Circumference Measurement Instructions

A. Standing position, with your shoulders and abdomen relaxed, breathing smoothly, feet separated by 25-30cm.

B. Horizontal position measured: midpoint of the line between the anterior superior iliac spine and the inferior margin of the 12th costal line on the midaxillary line.

C. Use a tape ruler around the abdomen in the above horizontal position. Measure with the tape ruler against the skin, but not against the skin.

D. Unable to consciously tuck or lift the abdomen during the measurement, take the reading at the calm end of expiration, and the waist circumference is in cm to the nearest mm (e.g., 89.3 cm).

Appendix 10 Hip Circumference Measurement Instructions

A. The subject will stand naturally, with shoulders relaxed, arms naturally drooped and moderately spread, legs together, weight bearing evenly on both legs, hips relaxed, and looking forward.

B. Horizontal position of measurement: symphysis pubis anteriorly and greater trochanter posteriorly. Generally equivalent to the most protruding part of the buttocks.

C. Use a tape measure to circle the buttocks horizontally and record the values.

Appendix 11 Blood Pressure Test Instructions

Mercury sphygmomanometer or electronic sphygmomanometer can be used for blood pressure measurement, but the same device can only be used for the same subject and cannot be replaced.

1. At the screening visit, blood pressure will be measured in both arms. If the difference in systolic or diastolic blood pressure is greater than 10mmHg, the arm with the higher blood pressure measured at the screening visit must be used for blood pressure measurements at subsequent visits.

2. Blood pressure (BP) measurement must be performed continuously throughout the study. Only the same arm should be used for BP measurement at each visit, and the arm used for measurement should be recorded. After ingestion of caffeine, alcohol, or nicotine, these measurements should be performed at intervals of at least 10 hours.

3. Subjects should rest for at least 5 minutes prior to measurement. For sitting blood pressure measurements, subjects should have their arms on a table with back support and feet on the floor.

4. It is required to repeat the measurement twice, with an interval of at least 1 minute each time. Calculate the mean blood pressure according to the two measurements (If the difference between the two measurements is large (above 5mmHg), it is necessary to make measurement again, and record the mean value of the 3 readings), and record it in the original medical record.

Supplementary Note 2: Statistical Analysis Plan

Statistical Analysis Plan

A Phase 2, Randomized, Double-Blind, Placebo-Controlled Study to Evaluate the Efficacy and Safety of IBI362 in Chinese Subjects with Overweight and Obesity

Protocol No.: CIBI362B201

Version No.: **1.0**
Version Date: **Sep. 29, 2022**

Statistician: **Yanqi Wang, Parasat Adil**

SIGNATURE APPROVAL PAGE

Author:

______________________________ Date: _______________

< Yanqi Wang, Senior Biostatistician >

Innovent Biologics (Suzhou) Co., Ltd.

______________________________ Date: _______________

< Parasat Adil, Biostatistician >

Innovent Biologics (Suzhou) Co., Ltd.

Approved by:

______________________________ Date: _______________

< Jie Wen, Senior Clinical Research Physician >

Innovent Biologics (Suzhou) Co., Ltd.

______________________________ Date: _______________

< Li Li, Associate Director, Biostatistics >

Innovent Biologics (Suzhou) Co., Ltd.

VERSION HISTORY

SAP Version No.	Approval Date	Description of Change
V1.0	2022-09-29	Final

ABBREVIATIONS

Abbreviations	Full name
ADA	Anti-drug Antibody
AE	Adverse Event
ALB	Albumin
ALP	Alkaline Phosphatase
ALT	Alanine Aminotransferase
ANC	Absolute Neutrophil Count
APTT	Activated Partial Thromboplastin Time
AST	Aspartate Aminotransferase
ANCOVA	Analysis of Covariance
BMI	Body Mass Index
BUN	Blood Urea Nitrogen
CK	Creatine Kinase
CK-MB	Creatine kinase-MB
Cr	Creatinine
C-SSRS	Columbia-Suicide Severity Rating Scale
Cl	Chlorine
Ca	Calcium
CTnI	Cardiac Troponin I
CTnT	Cardiac Troponin T
DBIL	Direct Bilirubin
DEXA	Dual Energy X-ray Absorptiometry
ECRF	Electronic Case Report Form
ECG	Electrocardiograms
FBG	Fasting Blood Glucose
FGF21	Fibroblast Growth Factor-21
FT3	Free Triiodothyronine
FT4	Free Thyroxine
GCP	Good Clinical Practice
GGT	Glutamyl Transpeptidase
HbA1c	Hemoglobin A1c
HCV	Hepatitis C Virus
HGB	Hemoglobin
HIV	Human Immunodeficiency Virus
HR	Heart Rate

HDL-C	High-Density Lipoprotein Cholesterol
HsCRP	High-sensitivity C-Reactive Protein
HOMA1-IR	Homeostatic Model Assessment for Insulin Resistance
HBcAb	Hepatitis B Core Antibody
HBeAb	Hepatitis B E Antibody
HBeAg	Hepatosis B E Antigen
HBsAb	Hepatitis B Surface Antibody
HBsAg	Hepatosis B Surface Antigen
ICH	International Conference on Harmonisation
INR	International Normalized Ratio
IWRS	Interactive Web Response System
IWQoL-Lite	Impact of Weight on Quality of Life-Lite
LDH	Lactate Dehydrogenase
LDL-C	Low-Density Lipoprotein Cholesterol
LOCF	Last Observation Carried Forward
MedDRA	Medical Dictionary for Regulatory Activities
MMRM	Mix model for Repeated Measurement
MRI	Magnetic Resonance Imaging
MRI-PDFF	Magnetic Resonance Imaging Derived Proton Density Fat Fraction
NAb	Neutralizing Antibody
OGTT	Oral Glucose Toleracnce Test
PLT	Platelet
PT	Prothrombin Time
PD	Pharmacodynamics
PK	Pharmacokinetics
PCSK9	Proproteinconvertase Subtilisin/Kexin Type 9
PHQ-9	Patient Health Questionnaire-9
Q1	First Quartile
Q3	Third Quartile
RBC	Red Blood Cell
REML	Restricted Maximum Likelihood
SAP	Statistical Analysis Plan
SAE	Severe Adverse Event
SFA	Subcutaneous Fat Area
SOC	System Organ Class
TBIL	Total Bilirubin

TEAE	Treatment Emergent Adverse Event
TRAE	Treatment-Related Adverse Event
TG	Triglyceride
TC	Total Cholesterol
TP	Total Protein
TFA	Total Intra-abdominal Fat Area
TSH	Thyroid Stimulating Hormone
UA	Uric Acid
UGLU	Urine Glucose
UPRO	Urine Protein
URBC	Urine Red Blood Cell
UWBC	Urinary White Blood Cell
VFA	Intraabdominal Fat Area
WBC	White Blood Cell
WHO	World Health Organization

TABLE OF CONTENTS

SIGNATURE APPROVAL PAGE **2**

VERSION HISTORY **3**

ABBREVIATIONS **4**

TABLE OF CONTENTS **7**

1 INTRODUCTION **9**

2 PROTOCOL DETAILS **9**

2.1 STUDY OBJECTIVES 9
2.2 TRIAL DESIGN 9
2.3 SAMPLE SIZE AND POWER 12
2.4 RANDOMIZATION AND BLINDING 12
2.5 CHANGES TO THE PLANNED STATISTICAL ANALYSES IN THE PROTOCOL 13
2.5.1 Changes to the Statistical Analysis Planned in the Protocol 13

3 ENDPOINTS **15**

3.1 PRIMARY ENDPOINT 15
3.2 SECONDARY EFFICACY ENDPOINTS 15
3.3 SAFETY ENDPOINTS 16
3.4 PHARMACOKINETIC AND PHARMACODYNAMIC ENDPOINTS 16
3.5 EXPLORATORY ENDPOINTS 16

4 ANALYSIS SETS **17**

4.1 ANALYSIS SETS 17

5 DATA PROCESSING **17**

5.1 GENERAL SPECIFICATIONS 17
5.2 SPECIFICATIONS FOR TREATMENT OF EFFICACY DATA 17
5.3 MISSING VALUES AND OUTLIERS 17
5.4 TIME POINTS AND VISIT WINDOWS 18

6 STATISTICAL ANALYSIS METHODS **18**

6.1 GENERAL PRINCIPLES 18
6.2 SUBJECT DISPOSITION 19
6.2.1 Subject Disposition 19
6.2.2 Protocol Deviations 19
6.3 DEMOGRAPHIC AND OTHER BASELINE CHARACTERISTICS 19
6.3.1 Demographic and Baseline Characteristics 19
6.3.2 Medical History 20
6.3.3 Prior/Concomitant Therapy 20
6.4 EFFICACY ANALYSIS 21
6.4.1 Analysis of Primary Efficacy Endpoint 21
6.4.2 Analysis of Secondary Efficacy Endpoints 23
6.4.3 Analysis of Exploratory Endpoints 25
6.4.4 Subgroup Analyses 26
6.4.5 Multiplicity 26
6.5 EXTENT OF STUDY DRUG EXPOSURE AND COMPLIANCE 26
6.6 SAFETY ANALYSIS 27
6.6.1 Adverse Events 27
6.6.2 Laboratory Tests 29

6.6.3 *Vital Signs* 30
6.6.4 *Electrocardiogram* 30
6.6.5 *Physical examination* 30
6.6.6 *C-SSRS Questionnaire* 30
6.6.7 *PHQ-9 questionnaire* 30
6.7 Quality Of Life Analysis 31
6.8 Immunogenicity Analysis 31
6.9 Pharmacokinetic Analysis 31
6.10 Pharmacodynamic Analysis 32
6.11 Interim Analysis And Data Monitoring Meetings 32
7 REFERENCES 32

1 Introduction

This document is the Statistical Analysis Plan (SAP) for Protocol CIBI362B201, which is prepared according to the Protocol, "A Randomized, Double-Blind, Placebo-Controlled Phase 2 Clinical Study to Assess the Efficacy and Safety of IBI362 in Chinese Overweight and Obese Subjects" (Protocol No. CIBI362B201, Version 5.0, dated 24 Aug 2022). This SAP summarizes the design and objectives of the protocol and is intended to provide detailed definitions of the endpoints in the protocol and a detailed description of the planned statistical analyses. The final SAP will be approved and signed prior to database lock in Stage 1 of the study.

2 Protocol Details

2.1 Study Objectives

- Primary Objective
    - To evaluate the change from baseline in body weight after administration of IBI362 for 24 weeks, and to recommend the appropriate dose for Phase 3 clinical trial.
- Secondary Objectives
    - To evaluate the safety of IBI362 administered for 24 weeks;
    - To evaluate the changes from baseline in indicators related to comorbidities after administration of IBI362 for 24 weeks;
    - To evaluate the rebound of body weight from baseline after administration of IBI362 for 24 weeks and 12 weeks after discontinuation;
    - To assess the population pharmacokinetic profile and pharmacodynamic profile of IBI362 in subjects with overweight and obesity.
- Exploratory Objectives
    - To explore the effect of IBI362 on the related indicators of metabolic-related fatty liver disease;
    - To explore the effect of IBI362 on body fat.

2.2 Trial Design

This will be a multicenter, double-blind, randomized, placebo-controlled study in subjects with Overweight or Obesity. In the first and second stages of this study, subjects will be enrolled and analyzed separately, with no influence on each other.

Approximately 240 subjects will be enrolled in the first stage of the study, and eligible subjects will receive a 2-week placebo lead-in, then will be randomized to 3.0 mg, 4.5 mg, and 6.0 mg dose groups and will be randomized in a 3: 1 ratio to receive IBI362 and placebo within each group; Randomization will be stratified by BMI < 28.0 kg/m^2 and BMI ≥ 28. 0 kg/m^2 at randomization. Subjects will receive weekly dosing for a total of 24 weeks of double-blind treatment. The dose regimens are as follows: 3.0

mg dose group will be titrated from 1.5 mg, 4 weeks later (4 doses) to 3.0 mg, and the treatment will be maintained for 20 weeks; The 4.5 mg dose group should be titrated from 1.5 mg to 3.0 mg after 4 weeks (4 doses), 3.0 mg to 4.5 mg after 4 weeks, and maintained for 16 weeks; The 6.0 mg dose group should be titrated from 2.0 mg to 4.0 mg after 4 weeks (4 doses), 4.0 mg to 6.0 mg after 4 weeks, and maintained for 16 weeks.

Approximately 80 subjects are planned to be enrolled in the second stage of the study, and eligible subjects will receive a placebo lead-in for 2 weeks and then be randomized at a ratio of IBI362: placebo = 3: 1 after successful lead-in. Subjects will be dosed weekly, starting at 3.0 mg and titrated to 6.0 mg after 4 weeks (4 doses) and 6.0 mg to 9.0 mg after 4 weeks, and maintained for 16 weeks.

The entire trial period consisted of a 3-week screening period, a 2-week placebo lead-in period, a 24-week double-blind treatment period, and a 12-week off-treatment follow-up period. Subjects will be required to maintain diet and exercise control throughout the study.

Dose Modification Criteria:

1. If the subject in the 4.5 mg dose group cannot tolerate the dose after reaching the specified target titration dose (4.5 mg) and the investigator considers that the patient cannot tolerate the dose for further exposure, after discussion with the sponsor, it is recommended that the dose can be reduced to 3.0 mg 2 weeks after reaching the specified target titration dose (4.5 mg) and maintained at this dose until the end of the trial.
2. If the subject in the 6.0 mg dose group cannot tolerate the dose after reaching the specified target titration dose (6.0 mg), and the investigator considers that the patient cannot tolerate the dose for further exposure, after discussion with the sponsor, it is recommended that the dose can be reduced to 4.5 mg 2 weeks after reaching the specified target titration dose (6.0 mg) and maintained at this dose until the end of the trial.
3. If the subject in the 9.0 mg dose group cannot tolerate the dose of 6.0 mg after reaching the titration dose (e.g., there is moderate to severe vomiting or diarrhea, and the gastrointestinal adverse reactions have not been relieved after symptomatic treatment and/or 1-week interruption of the dose), and the investigator considers that the patient cannot tolerate the dose for further exposure, the dose can be reduced to 3.0 mg after the discussion with the sponsor, and the dose can be re-increased to 6.0 mg 2 weeks later according to the subject's condition. If the dose is still intolerable after increasing to 6.0 mg, the dose can be adjusted back to 3mg and maintained at this dose until the end of the study; If the 6.0 mg dose will be tolerated, the dose will be continued increased to 9.0 mg 4 weeks later according to the subject's condition.
4. If the subject in the 9.0 mg dose group can not tolerate the dose after reaching the specified target titration dose (9.0 mg) (e.g., there is moderate to severe vomiting or diarrhea, gastrointestinal adverse reactions remain unresolved after symptomatic treatment and/or 1-week interruption), and the investigator considers that the patient cannot tolerate the dose for further exposure, the dose can be reduced to 6.0 mg after the discussion with the sponsor and maintained at this dose until

the end of the study or continued to be up-titrated to 9.0 mg after 2 weeks of 6.0 mg according to the subject's condition.

The study design is shown in Figure 1:

Stage 1:

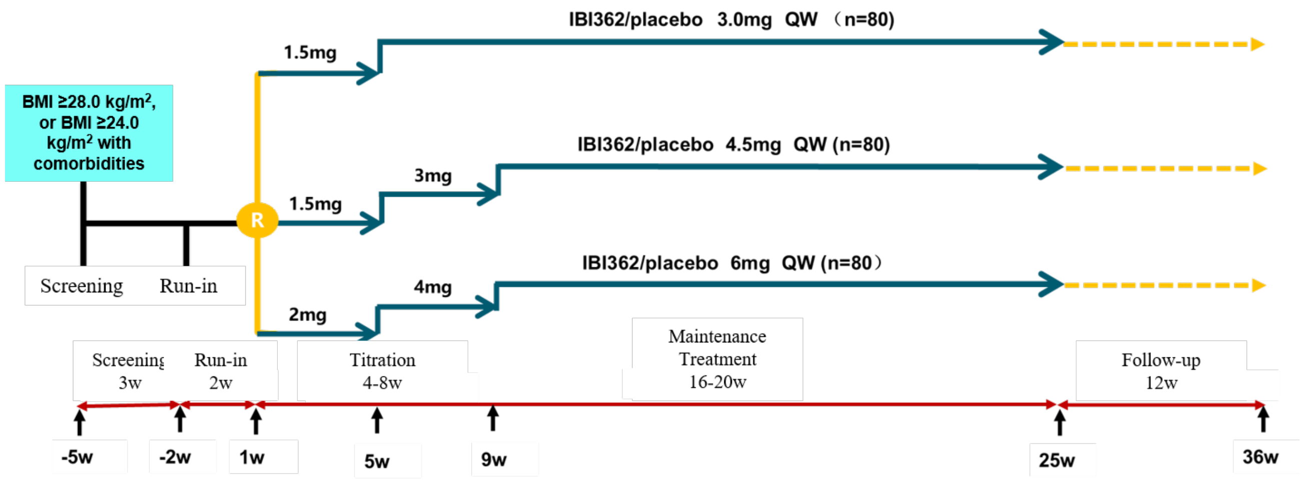

1. If the subjects in the 4.5 mg dose group still cannot tolerate the dose after reaching the specified target titration dose (4.5 mg), and the investigator considers that the patients cannot tolerate the dose for further exposure, after discussion with the sponsor, it is recommended that the dose can be reduced to 3.0 mg after reaching the specified target titration dose (4.5 mg) for 2 weeks, and the dose can be maintained until the end of the trial.

2. If the subject in the 6.0 mg dose group still cannot tolerate the dose after reaching the specified target titration dose (6.0 mg), and the investigator thinks that the patient cannot tolerate the dose for further exposure, after discussion with the sponsor, it is recommended that the dose can be reduced to 4.5 mg 2 weeks after reaching the specified target titration dose (6.0 mg) and maintained at this dose until the end of the trial.

Stage 2:

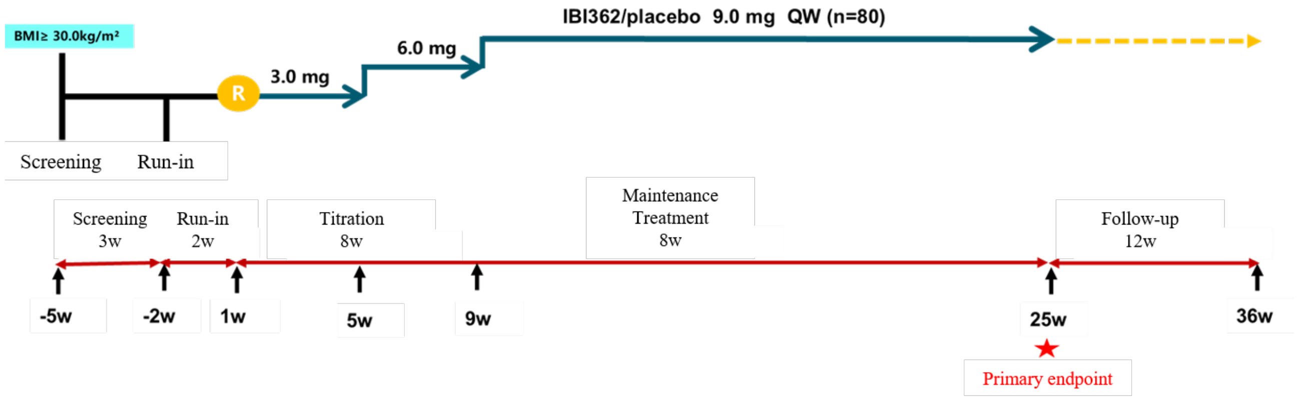

1. If the subject in the 9.0 mg dose group cannot tolerate the dose of 6.0 mg after reaching the titration dose (e.g., there is moderate to severe vomiting or diarrhea, and the gastrointestinal adverse reactions have not been relieved after symptomatic treatment and/or 1-week interruption of the dose), and the investigator considers that the patient cannot tolerate the dose for further exposure, the dose can be reduced to 3.0 mg after the discussion with the sponsor, and the dose can be re-increased to 6.0 mg 2 weeks later according to the subject's condition. If the dose is still intolerable after up-titration to 6.0 mg, the dose can be adjusted back to 3mg and maintained at this dose until the end of the study; If the 6.0 mg dose will be tolerated, the dose will be continued up-titrated to 9.0 mg 4 weeks later according to the subject's condition.

2. If the subject in the 9.0 mg dose group cannot tolerate the dose after reaching the specified target titration dose (9.0 mg) (e.g., there is moderate to severe vomiting or diarrhea, gastrointestinal adverse reactions remain unresolved after symptomatic treatment and/or 1-week interruption), and the investigator considers that the patient cannot tolerate the dose for further exposure ,the dose can be reduced to 6.0 mg after the discussion with the sponsor and maintained at this dose until the end of the study or continued to be up-titrated to 9.0 mg after 2 weeks of 6.0 mg according to the subject's condition.

Figure 1 Schematic of Study Design

2.3 Sample Size And Power

For stage 1, assuming a weight loss of approximately 2.3% from baseline after 24 weeks in the placebo group, a weight loss of up to approximately 10% from baseline after 24 weeks in the IBI362 group, a standard deviation of weight loss of approximately 8%, a dropout rate of approximately 20%, an observed efficacy of 8%, and α = 0.05 (two-sided), the sample size of approximately 80 subjects per dose group (approximately 240 subjects in total) will provid a power > 95%, that at least one of the doses of IBI362 would result in superior weight loss versus pooled placebo.

For stage 2, assuming a body weight loss of approximately 2.3% from baseline after 24 weeks in the placebo group and 12% from baseline after 24 weeks in the IBI362 group, with a standard deviation of approximately 8%, a dropout rate of approximately 20% and an observed efficacy of approximately 10%, α = 0.05 (two-sided), a total of approximately 80 subjects will provide a power > 95%, that IBI362 9.0 mg would result in superior weight loss versus placebo.

2.4 Randomization And Blinding

Subjects will be randomly assigned in a 3: 1 ratio to IBI362 and placebo in each dose group using a block randomization method.

Subject randomization will be completed by the IWRS. Successfully randomized subjects will be given a randomization number and will receive drug treatment according to the drug number assigned by the system. The unblinded statistician will complete the blinding of the subject's randomization number and medication number, which will be transferred to the unblinded administrator of the randomization system to complete the preparation and management of IWRS blinding.

A central randomization method will be used, with competing enrollments from each center. A central randomization system will be used for the central randomization procedure. If the subject completes all screening assessments of the study and meets the enrollment criteria, he/she will receive a randomization number generated by IWRS, which will connect the subject to the assigned treatment group and can be assigned to dispense the investigational product according to the amount required. The randomization number of a randomized subject will be retained regardless of withdrawal from the study for any reason. IWRS personnel will only be responsible for the configuration and management of the randomization system and will not be involved in any specific trial operations.

The subjects in the two stages of this study will be enrolled and analyzed separately, and the operation and analysis of the two stages of the study will be not affected by each other. The two stages of the study will be unblinded separately.

Take the first stage as an example. All subjects in stage 1 and the investigators, monitors, sponsor personnel, and representatives involved in their treatment or clinical evaluation remained blinded until all subjects in stage 1 completed their week 25 visit. After all subjects in stage 1 have completed the week 25 visit, the subjects in stage 1 and all investigators, monitors and sponsor personnel in contact with the investigators and subjects who participate in the treatment or clinical evaluation will continue to remain blinded until the end of stage 1, and other sponsor personnel will not remain blinded.

The second stage of blinding maintenance as above.

Unblinding of subjects will be performed in each of the two stages. After all subjects in each stage dosing for 24 weeks, the sponsor will be unblinded after completion of the week 25 visit and after the primary endpoint database for these subjects is locked. Investigators and subjects will remain blinded until the end of this stage of the study, i.e., after the end of the first stage, all investigators, subjects in the first stage and sponsor personnel will be unblinded; All investigators, stage 2 subjects, and sponsor personnel will be unblinded at the end of the stage 2 study.

In the clinical study, the investigator may unblind the subject in case of emergency medical events due to the safety of the subject when the investigator needs to know the investigational drug used by the subject. Emergency unblinding must be performed in the system by authorized personnel designated by the site as per procedure. Before unblinding of the investigational drug, relevant personnel of the sponsor should be notified. After obtaining the approval from the principal investigator and the sponsor, the subject should be entered into the IWRS system for emergency unblinding so as to obtain the specific grouping information of the subjects. The investigator should record the time, location and reason for unblinding (the grouping information after unblinding should not be recorded on the eCRF).

2.5 Changes To The Planned Statistical Analyses In The Protocol

2.5.1 Changes to the Statistical Analysis Planned in the Protocol

**Protocol**	**Statistical Analysis Plan**	
**Statistical Description in Protocol**	**Statistical Description in Statistical Analysis Plan**	**Rationale for change**
Not described in the original protocol.	Exploratory Endpoint: change from baseline in HOMA-IR after 24 weeks of study drug administration.<br>Exploratory Endpoint: change from baseline in HOMA-IR after 24 weeks of study drug administration and 12 weeks of discontinuation.	Added exploratory endpoints.

Protocol	Statistical Analysis Plan	
**Statistical Description in Protocol**	**Statistical Description in Statistical Analysis Plan**	**Rationale for change**
	Exploratory endpoint: change and Percent change of aspartate aminotransferase from baseline after 12 weeks and 24 weeks of study drug administration.	
Changes from baseline in waist circumference, BMI, HbA1c, fasting plasma glucose, systolic blood pressure, diastolic blood pressure and blood lipids (TC, TG, LDL-C and HDL-C) after 24 weeks of study drug administration and 12 weeks of discontinuation.	Changes from baseline in waist circumference, BMI, systolic blood pressure and diastolic blood pressure after 24 weeks of study drug administration and 12 weeks of discontinuation.	No central laboratory data will be collected for HbA1c, fasting plasma glucose, and lipids (TC, TG, LDL-C, HDL-C) in the safety follow-up.
Other laboratory indicators: blood amylase and blood lipase	Other laboratory indicators: blood amylase, blood lipase, calcitonin	added calcitonin
Blood biochemistry: liver function (TBIL, DBIL, ALT, AST, GGT, ALP, ALB, TP, LDH), renal function (BUN, UA, Cr), blood electrolytes (Na, K, Cl, Ca), FBG	Liver function (serum total bilirubin (TBIL), direct bilirubin (DBIL), alanine aminotransferase (ALT), aspartate aminotransferase (AST), aminoacyl transpeptidase (GGT), alkaline phosphatase (ALP), albumin (ALB), total protein (TP), lactate dehydrogenase (LDH)), renal function (urea/urea nitrogen (BUN), uric acid (UA), creatinine (Cr), blood electrolytes (sodium (Na), potassium (K), chloride (Cl), calcium (Ca))	FBG will be removed and analysed as an efficacy endpoint.
Safety set (SS): Subjects who signed informed consent and took at least one dose of study drug.	Safety Analysis Set: Subjects who signed informed consent and took at least one dose of study drug.	Revision
Modified Intent-to-Treat Analysis Set (mITT): Subjects who will be a subset of the Safety Set and had at least one post-baseline assessment.	Modified Intent-to-Treat Analysis Set: Subjects who will be a subset of the Safety Analysis Set and had baseline and at least one post-baseline body weight assessment.	Revision

Protocol	Statistical Analysis Plan	
**Statistical Description in Protocol**	**Statistical Description in Statistical Analysis Plan**	**Rationale for change**
PD analysis set: including all subjects who received at least one dose of study drug and had at least one valid test result at baseline and after drug administration.	Pharmacodynamic analysis set: subjects who are a subset of the safety analysis set and have baseline and at least one post-baseline valid pharmacodynamic parameter test result.	Revision

3 Endpoints

Refer to Table 1 and Table 2 of the clinical study protocol for the assessment schedule of endpoint in this trial.

3.1 Primary Endpoint

- Percent change (%) of body weight from baseline after 24 weeks of administration.

3.2 Secondary Efficacy Endpoints

- The proportion of subjects with body weight loss ≥ 5.0% from baseline after administration for 24 weeks;
- The proportion of subjects with body weight loss ≥ 10.0% from baseline after administration for 24 weeks;
- Change from baseline in body weight (Kg) after administration for 24 weeks;
- Changes from baseline in waist circumference, BMI, glycated hemoglobin A1c (HbA1c), fasting plasma glucose, systolic blood pressure, diastolic blood pressure, blood lipids (TC, TG, LDL-C, HDL-C) after administration for 24 weeks;
- Percent change (%) of body weight from baseline after 24 weeks of study drug administration and 12 weeks of discontinuation;
- Change in body weight (kg) from baseline after 24 weeks of study drug administration and 12 weeks of discontinuation;
- The proportion of subjects with body weight loss ≥ 5.0% and 10.0% from baseline after treatment for 24 weeks and 12 weeks of discontinuation;
- Changes from baseline in waist circumference, BMI, systolic blood pressure and diastolic blood pressure after 12-week discontinuation after administration for 24 weeks;
- To evaluate the changes from baseline in serum uric acid and alanine aminotransferase levels after 12 and 24 weeks of administration;
- To evaluate the improvement of quality of life (IWQoL-Lite questionnaire) after 24 weeks of

administration.

3.3 Safety Endpoints

- To evaluate the safety of IBI362 in subjects with different doses (adverse events, hypoglycemic events, vital signs, physical examination, laboratory tests, 12-lead ECG, etc.);
- Evaluate the mental health status of subjects after administration (C-SSRS questionnaire, PHQ-9 questionnaire);
- Incidence of anti-drug antibody (ADA) and neutralizing antibody (NAb) against IBI362 in serum before and after administration.

3.4 Pharmacokinetic And Pharmacodynamic Endpoints

- To assess the population pharmacokinetic and pharmacodynamic profile of IBI362 in subjects with overweight or obesity:
  - The population pharmacokinetic profile of IBI362 will be analyzed using nonlinear mixed effects modeling;
  - Pharmacodynamic parameters, including changes in fasting plasma glucose and fasting insulin at different time points before and after administration.

3.5 Exploratory Endpoints

- To evaluate the changes of high-sensitivity C-Reactive Protein (hsCRP), Proproteinconvertase Subtilisin/Kexin Type 9 (PCSK9), Fibroblast Growth Factor-21 (FGF-21) and adiponectin levels from baseline after administration for 24 weeks;
- The total fat mass, regional visceral fat mass and total lean body mass measured by Dual Energy X-ray Absorptiometry (DEXA) will be evaluated, and the changes of each index from baseline at week 24 of dosing;
- Evaluate the intra-abdominal fat area (VFA), subcutaneous fat area (SFA) and total intra-abdominal fat area (TFA) measured by Magnetic Resonance Imaging (MRI), and the changes of each index from baseline after administration for 24 weeks;
- To assess the liver fat content as measured by Magnetic Resonance Imaging Derived Proton Density Fat Fraction (MRI-PDFF), and compare the change from baseline after 24 weeks of dosing.
- Change from baseline in HOMA-IR after 24 weeks of continuous administration.
- To evaluate the change and Percent change of aspartate aminotransferase from baseline after 12 and 24 weeks of administration.

4 Analysis Sets

4.1 Analysis Sets

All Screened Subjects: Subjects who have signed informed consent and have been assigned a subject number.

Randomized Subjects: Subjects who signed informed consent and will be randomized to the study.

Safety Analysis Set: Subjects who signed informed consent and took at least one dose of study drug.

Modified Intent-to-Treat Analysis Set: Subjects who will be a subset of the Safety Analysis Set and had baseline and at least one post-baseline body weight assessment.

Pharmacodynamic analysis set: Subjects who are a subset of the safety analysis set and have baseline and at least one post-baseline valid pharmacodynamic parameter test result.

5 Data Processing

5.1 General Specifications

The entire trial consisted of a 3-week screening period, a 2-week placebo lead-in period, a 24-week double-blind treatment period, and a 12-week off-treatment follow-up period.

5.2 Specifications For Treatment Of Efficacy Data

In this trial, efficacy data will not be processed and missing values will not be imputed unless otherwise specified. Missing data imputation for primary and secondary efficacy variables will be described in detail in 6.2.

5.3 Missing Values And Outliers

- Handling of Completely/Partially Missing Values for Prior/Concomitant Medications/Non-Drug Therapy Dates
  - Prior/Concomitant Medication/Non-Medication Start/End Date or Time completely or partially missing will not be imputed. Complete or partial absence cannot be identified as prior or concomitant and will be considered concomitant.
- Handling of Completely/Partially Missing Values for Adverse Event Dates
  - An adverse event is considered to be on-treatment if the start date of the event is completely or partially missing and the data collected on the eCRF is not clear whether it is before or after the first dose of study medication. Unless otherwise specified, completely or partially missing adverse event dates will not be imputed.
- If start date/time of first study medication is missing, mark the event as a medication period adverse event
  - Adverse events that occurred on or after the day of randomization will be considered treatment-emergent adverse events.

The above imputation of completely/partially missing dates will be only used to flag or calculate new variables for summary, and the original date recorded on the eCRF will be still presented in the listings.

- Missing relationship of adverse event to study drug
  - An adverse event with a missing relationship to study drug is considered related.
- Severity of adverse event
  - In summaries by severity, adverse events with missing severity will be counted in the number/frequency of severe events.
  - Subjects with missing baseline values will be classified as "missing" in the shift tables summarizing baseline and worst post-baseline results.
- Outlier Handling
  - There will be no planned outlier checks and treatments for this trial.
- Other Data Handling Considerations
  - If the reported values for laboratory parameters cannot be used in the statistical summary tables (e.g., values for numeric parameters are reported as strings), the coded values will be appropriately determined and applied to the statistical analysis. Generally, 0.0001 will be added when the upper limit of detection is exceeded, for example, "> 100" will be regarded as "100.0001"; Usually below the lower limit of detection will be substrated 0.0001, e.g. "< 100" will be treated as "99.9999". The actual values reported in the database will still be presented in the listings.

5.4 Time Points And Visit Windows

In the statistical analysis of summary by visit, only scheduled visits will be used.

Unscheduled visit data will be used for baseline value flagging. Unscheduled visit data of laboratory tests, vital signs, ECG and other safety variables will be used for analysis of abnormality changes before and after baseline (shift table), and will not be used for summary analysis by visit.

6 Statistical Analysis Methods

6.1 General Principles

All statistical analyses will be performed using SAS 9.4 (or higher).

Categorical varibles will be summarized using frequency (percentage). Unless otherwise specified, the number of subjects in each treatment group in the corresponding analysis set will be used as the denominator for percentages. Percentages will be rounded to 1 decimal place.

Continuous varibles will be summarized using the number of subjects, mean (standard deviation), median (Q1, Q3), minimum and maximum. Means and medians will be to 1 more decimal place than

the raw data, standard deviations will be to 2 more decimal places than the raw data, and minimum and maximum will be to the same decimal place as the raw data. For the PK parameters, please refer to the PK analysis plan for details.

Baseline value: defined as the last available measurement prior to the first dose of study medication.

Subject disposition, major protocol deviations, demographic and other baseline characteristics, medical history, prior/concomitant medications, efficacy analyses, exposure of study drug, compliance, safety analyses, quality of life, immunogenicity, and pharmacodynamic will be summarized separately by study stage (Stage 1 and Stage 2).

6.2 Subject Disposition

6.2.1 Subject Disposition

Subject disposition includes subject screening, subject disposition, and analysis set.

Subject screening will be summarized as categorical varibles for all screened subjects, including entry to screening, screen failure, lead-in failure, screen success, and primary reason for screen failure.

Subject disposition will be summarized by planned treatment group as categorical varibles for randomized subjects, including randomization, early discontinuation from the double-blind treatment period, reason for early discontinuation from the double-blind treatment period, early discontinuation from the study, and reason for early discontinuation from the study.

The analysis set will be summarized as categorical varibles for randomized subjects including Safety Analysis Set, Modified Intent-to-Treat Analysis Set, and Pharmacodynamic Analysis Set.

Among randomized subjects, randomization schemes, a list of randomization numbers, a list of subjects who prematurely withdrew from the study, and a list of subjects excluded from the modified intent-to-treat analysis set will be provided.

6.2.2 Protocol Deviations

Major protocol deviations will be summarized as categorical varibles by planned treatment group and type of protocol deviation in randomized subjects.

In randomized subjects, a listing of major protocol deviations will be provided.

6.3 Demographic And Other Baseline Characteristics

Demographic and other baseline characteristics include demographics, baseline characteristics, medical history, prior medications, concomitant medications, prior non-drug therapies, and concomitant non-drug therapies.

6.3.1 Demographic and Baseline Characteristics

Demographic and baseline characteristics will be summarized by planned treatment group as categorical or continuous varibles in randomized subjects.

Demographics includes the following variables: baseline age (years), gender (male, female), female of childbearing potential (yes, no), ethnicity (Han, Other), baseline height (cm), baseline fasting body weight (kg), baseline fasting body weight groups (< 50, ≥ 50 to < 70, ≥ 70 to < 90, and ≥ 90 kg), baseline body mass index (kg/m^2), baseline body mass index groups (< 24, ≥ 24 to < 28, ≥ 28 kg/m^2) (only applicable to Stage 1 of the study), and alcohol use status (not drinking, drinking, and abstinent). Baseline age (years) will be calculated as: (date of informed consent-date of birth + 1)/365.25, whichever is the smallest integer. Baseline body mass index (kg/m^2) will be calculated as baseline weight (kg)/(baseline height (m)) ^{2}. Females of childbearing potential are defined as those who have had menarche, have not undergone sterilization (i.e., bilateral tubal ligation, bilateral salpingectomy, or total hysterectomy), and have not yet reached menopause.

Baseline characteristics includes the following variables: type of subject (overweight, obese), overweight with manifestation 1 (Strong appetite, hunger before meal, more food intake per meal), overweight with manifestation 2 (Comorbid with one or more of pre-diabetes (impaired fasting glucose and/or impaired glucose tolerance), hypertension, dyslipidemia and fatty liver (within 6 months prior to screening)), overweight with manifestation 3 (combined with weight-bearing joint pain), and overweight with manifestation 4 (obesity-induced dyspnea or obstructive sleep apnea syndrome).

In randomized subjects, baseline measures will be summarized as continuous varibles by planned treatment group, including baseline waist circumference (cm), baseline hip circumference (cm), baseline serum uric acid (umol/L), baseline alanine aminotransferase (U/L), baseline aspartate aminotransferase (U/L), baseline triglycerides (mmol/L), baseline total cholesterol (mmol/L), baseline LDL-C (mmol/L), baseline HDL-C (mmol/L), baseline glycated hemoglobin (%), baseline fasting plasma glucose (mmol/L), baseline systolic blood pressure (mmHg), baseline diastolic blood pressure (mmHg), baseline fasting insulin (mU/L), and baseline HOMA1-IR. Where HOMA1-IR = (fasting insulin (mU/L) × fasting plasma glucose (mmol/L))/22.5.

In randomized subjects, a listing of demographic and baseline characteristics will be provided.

6.3.2 Medical History

The MedDRA dictionary (version 25.0 or higher) will be used to code the system organ class (SOC) and preferred term (PT) of medical history.

Medical history will be summarized by planned treatment group using SOC and PT as categorical varibles in randomized subjects. If a subject had more than one medical history within the same SOC or PT category, the subject will be counted only once within that SOC and PT category.

In randomized subjects, a listing of medical, alcohol, and allergy history will be provided.

6.3.3 Prior/Concomitant Therapy

Prior/Concomitant Medications

The WHO-DD dictionary (version WHODrug 2022Mar or higher) will be used to code Anatomical Therapeutic Chemical (ATC) level 2 and 4 terms and drug names (PN) for prior and concomitant medications, respectively.

Prior and concomitant medications will be summarized as categorical varibles by planned treatment group using ATC Level 2, ATC Level 4, and PN in randomized subjects. If a subject had more than one concomitant medication in the same ATC level 2 or PN category, the subject will be counted only once in that ATC level 2 or PN category.

Prior medications will be defined as medications that started and ended prior to the first study medication.

Concomitant medications will be defined as medications taken from the start of the first study medication to the last study medication + 56 days, including medications that started before the first study medication and will be ongoing after the first study medication, and medications taken from the day of the first study medication to the last medication + 56 days.

Prior/Concomitant Non-Drug Therapies

The MedDRA dictionary (version 25.0 or higher) will be used to code the system organ class (SOC) and preferred term (PT) for prior and concomitant non-drug therapies.

Prior and concomitant non-drug therapies will be summarized as categorical varibles by planned treatment group using SOC and PT in randomized subjects. If a subject had more than one prior and concomitant non-drug therapy within the same SOC or PT category, the subject will be counted only once within that SOC and PT category.

Prior non-drug therapies are defined as non-drug therapies that have been initiated prior to the first study medication and have been discontinued prior to the first study medication.

Concomitant non-drug therapies are defined as non-drug therapies that started or will be ongoing from the first dose of study medication to the last dose of study medication + 56 days, including non-drug therapies that started before the first dose of study medication and will be ongoing after the first dose of study medication, and non-drug therapies that started on the day of the first dose of study medication to the last dose of study medication + 56 days.

In randomized subjects, a listing of prior/concomitant medications and prior/concomitant non-drug therapies will be provided.

6.4 Efficacy Analysis

Efficacy analyses will be analyzed in the modified intent-to-treat analysis set.

6.4.1 Analysis of Primary Efficacy Endpoint

Primary Analysis of Primary Efficacy Endpoint

The primary efficacy endpoint will be the percent change (%) from baseline in body weight after 24 weeks of continuous dosing. The primary efficacy endpoint will be analyzed using an ANCOVA

model with treatment group and the randomization stratification factors (24.0 to < 28.0 kg/m^2 vs. BMI ≥ 28.0 kg/m^2) as fixed effects and baseline body weight as a covariate for the first stage of the study. For the second stage of the study, treatment group will be included as a fixed effect and baseline body weight will be included as a covariate in the ANCOVA model. For missing value of body weight at week 25, the LOCF method will be used.

Baseline body weight and body weight at week 25 will be presented as continuous data by planned treatment group, and the adjusted least squares mean (standard error) and 2-sided 95% confidence interval will be estimated for body weight after 24 weeks of dosing (Week 25) for each planned treatment group, and the point estimate for the difference between each IBI362 dose group and pooled placebo will be estimated, along with the corresponding standard error, 2-sided 95% confidence interval, and p-value.

Sensitivity Analyses of the Primary Efficacy Endpoint

A sensitivity analysis of the primary efficacy endpoint will also be performed using the Mix-Effect Model for Repeated Measurement (MMRM). For the first stage of the study, the MMRM model (using PROC MIXED process step in SAS) included treatment group, visit (Weeks 2, 3, 4, 5, 6, 7, 8, 9, 10, 11, 13, 17, 21, 24), treatment group-by-visit interaction, and the randomization stratification factor (24.0 ≤ BMI < 28.0 kg/m^2 vs. BMI ≥ 28.0 kg/m^2) as fixed categorical effects, and baseline weight value and baseline weight value-by-visit interaction as fixed continuous covariates. Within-subject errors will be modeled using an unstructured correlation matrix in the model. Parameters will be estimated using the Restricted Maximum Likelihood (REML) method, calculated using the Newton-Raphson algorithm. Denominator degrees of freedom will be estimated using the Kenward-Roger approximation. For the second stage of the study, the MMRM model included treatment group, visit (Weeks 3, 5, 9, 13, 17, 21, 24), treatment group by visit interaction as fixed categorical effects, and baseline weight value and baseline weight by visit interaction as fixed continuous covariates. Within-subject errors will be modeled using an unstructured correlation matrix in the model. Parameters will be estimated using the Restricted Maximum Likelihood (REML) method, calculated using the Newton-Raphson algorithm. Denominator degrees of freedom will be estimated using the Kenward-Roger approximation.

Baseline body weight and body weight at week 25 will be presented as continuous data by planned treatment group, and the adjusted least squares mean (standard error) and 2-sided 95% confidence interval will be estimated for body weight at week 25 for each planned treatment group, and the point estimate for the difference between each IBI362 dose group and pooled placebo will be estimated with the corresponding standard error, 2-sided 95% confidence interval, and p-value to test whether the treatment group difference is significant. Mean body weight, change from baseline in body weight, and percent change from baseline in body weight will be summarized by visit, and mean body weight, mean change from baseline, and mean percent change from baseline in body weight over time will be plotted by visit.

A listing of body weight will also be provided.

6.4.2 Analysis of Secondary Efficacy Endpoints

Continuous endpoint

The following continuous secondary efficacy endpoints will be analyzed using the same MMRM model as for the primary efficacy endpoint in Section 6.4. 1. For the first stage of the study, the MMRM model included treatment group, visit (visits will be confirmed according to the scheduled measurement visit for different variables), treatment group-by-visit interaction, and randomization stratification factors (24.0 ≤ BMI < 28.0 kg/m^2 vs. BMI ≥ 28.0 kg/m^2) as fixed categorical effects, and baseline value and baseline value-by-visit interaction as fixed continuous covariates. Within-subject errors will be modeled using an unstructured correlation matrix in the model. Parameters will be estimated using the Restricted Maximum Likelihood (REML) method, calculated using the Newton-Raphson algorithm. Denominator degrees of freedom will be estimated using the Kenward-Roger approximation. For the second stage of the study, the MMRM model included treatment group, visit (visits will be confirmed according to the scheduled measurement visits for different variables), treatment group-by-visit interaction as fixed categorical effects, and baseline value and baseline value-by-visit interaction as fixed continuous covariates. Within-subject errors will be modeled using an unstructured correlation matrix in the model. Parameters will be estimated using the Restricted Maximum Likelihood (REML) method, calculated using the Newton-Raphson algorithm. Denominator degrees of freedom will be estimated using the Kenward-Roger approximation.

Measurements of each of the following secondary efficacy variables at baseline and after 24 weeks of treatment (Week 25) will be presented as continuous data by planned treatment group, and the adjusted least squares mean (standard error) and two-sided 95% confidence interval will be estimated for each of the planned treatment groups after 24 weeks of treatment (Week 25), and the point estimate of the difference between each IBI362 dose group and pooled placebo will be estimated, along with the corresponding standard error, two-sided 95% confidence interval and p-value to test whether the treatment group difference is significant.

For blood lipids (TC, TG, LDL-C, HDL-C), serum uric acid and alanine aminotransferase, the changes and Percent changes of corresponding indicators from baseline after continuous administration for 24 weeks will be analyzed. For waist circumference, BMI, glycosylated hemoglobin, fasting plasma glucose, systolic blood pressure and diastolic blood pressure, the changes of corresponding indicators from baseline after continuous administration for 24 weeks will be analyzed. For waist circumference, BMI, systolic blood pressure and diastolic blood pressure, the changes of corresponding indicators from baseline after 12 weeks of discontinuation after administration for 24 weeks will be analyzed.

- Change from baseline in body weight (Kg) after 24 weeks of study drug administration.
- Changes from baseline in waist circumference, BMI, glycosylated hemoglobin A1c (HbA1c), fasting plasma glucose, systolic blood pressure, diastolic blood pressure and blood lipids (TC, TG, LDL-C and HDL-C) after 24 weeks of study drug administration.

- Percent change (%) of body weight from baseline after 24 weeks of study drug administration and 12 weeks of discontinuation.
- change from baseline in body weight (Kg) after 24 weeks of study drug administration and 12 weeks of discontinuation.
- Changes from baseline in waist circumference, BMI, systolic blood pressure and diastolic blood pressure after 24 weeks of study drug administration and 12 weeks of discontinuation.
- To evaluate the changes from baseline in serum uric acid and alanine aminotransferase levels after 12 weeks and 24 weeks of study drug administration.

Change from baseline and percent change from baseline of the following efficacy indicators will be summarized by visit by planned treatment group, and the mean value over time, change from baseline and percent change from baseline of these indicators will be plotted by visit by planned treatment group.

- TC
- TG
- LDL-C
- HDL-C
- Serum uric acid
- ALT

Changes from baseline of the following secondary efficacy indicators will be summarized descriptively by visit in the form of measurement data in each planned treatment group, and the mean values of these indicators over time and the visit figures of changes from baseline will be plotted by planned treatment group.

- Waist circumference
- BMI
- HbA1c
- Fasting blood glucose
- Systolic blood pressure
- Diastolic blood pressure

Categorical endpoint indicators

For the following secondary efficacy endpoints, a logistic regression model will be used. For Stage 1 and Stage 2 of the study, the planned treatment group and baseline body weight will be included in the model as independent variables, and the subjects who lost ≥ 5.0% or 10.0% of body weight from

baseline after 24 weeks of continuous dosing (Week 25) (yes or no) and the subjects who lost ≥ 5.0% or 10.0% of body weight from baseline after 24 weeks of study drug administration and 12 weeks of discontinuation (yes or no) will be included as dependent variables to fit a logistic regression model. Subjects with missing data at week 25 will be imputed as non-responder, i.e. body weight reduction form baseline will be considered as less than 5.0% or 10.0%..

The number and percentage of subjects with body weight ≥ 5. 0% and ≥ 10. 0% from baseline in the week after 24 weeks of dosing (Week 25) will be estimated for each planned treatment group, and the odds ratio, 2-sided 95% confidence interval, and p-value will be provided.

- The proportion of subjects with body weight loss ≥ 5. 0% from baseline after 24 weeks of continuous administration.
- The proportion of subjects with body weight loss ≥ 10. 0% from baseline after 24 weeks of continuous administration.
- The rate of subjects with body weight loss ≥ 5. 0% and 10.0% from baseline after treatment for 24 weeks and 12 weeks of discontinuation.

The analysis methods for the secondary efficacy endpoint of evaluating the improvement of quality of life (IWQoL-Lite questionnaire) after 24 weeks of dosing are described in Section 6.7.

6.4.3 Analysis of Exploratory Endpoints

The following exploratory endpoints will be analyzed using the methods described in Section 6.4. 2 for continuous secondary efficacy endpoints.

- To evaluate the change and Percent change of aspartate aminotransferase from baseline after 12 and 24 weeks of administration.

Change from baseline and percent change from baseline of the following exploratory endpoints will be summarized descriptively by visit for each planned treatment group, and the mean, change from baseline and percent change from baseline over time will be plotted by visit for the following variables by planned treatment group.

- hsCRP
- PCSK9
- FGF21
- Adiponectin
- Total fat content
- Local visceral fat content
- Total lean body mass
- Intra-abdominal fat area

- Subcutaneous fat area
- Total abdominal fat area
- Liver fat content
- HOMA-IR
- AST

6.4.4 Subgroup Analyses

In the modified intent-to-treat analysis set, body weight after 24 weeks of treatment, change and Percent change from baseline in body weight after 24 weeks of treatment will be summarized in the following subgroups:

- Gender (male and female)
- Age (divided into two subgroups by median age: < median and ≥ median)
- BMI (≥ 24 and < 28 and ≥ 28 kg/m^2 (only applicable to Stage 1 of the study) ; < median baseline BMI and ≥ median baseline BMI)

6.4.5 Multiplicity

No adjustment for multiplicity will be conducted in this trial.

6.5 Extent Of Study Drug Exposure And Compliance

Extent of exposure to study drug and compliance will be summarized by actual treatment group in the safety analysis set, including days of exposure (days), weeks of exposure (weeks), number of doses (times), actual cumulative exposure (mg), and compliance (%). The number of doses will be classified as 1-4, 5-8, 9-12, 13-16, 17-20, 20-24.

The following definitions are for days of exposure, weeks of exposure, planned cumulative exposure, actual cumulative exposure, and medication compliance:

- Days of exposure (days) = last dose date-first dose date + 7 days
- Weeks of exposure (weeks) = days of exposure/7 to 1 decimal place.
- Planned times of administration: the sum of planned times of administration of the subject. The planned times of administration up to the last actual treatment, and the drug interrupted due to medical order are not the planned times of administration.
- Actual times of administration: the sum of the actual times of administration by the subject.
- Actual cumulative exposure (mg): the sum of the actual doses taken by the subject.
- Medication compliance (%) = (actual times of medication/planned times of medication) × 100%

Listings of drug exposure will be provided in safety analysis set.

6.6 Safety Analysis

Safety analyses will be performed on the Safety Analysis Set and will be assigned by actual treatment. Safety variables include adverse events, laboratory tests, vital signs, electrocardiograms, and physical examinations.

6.6.1 Adverse Events

Adverse events will be classified by the date of event onset:

- Pre-treatment adverse events: adverse events occurring from the signing of informed consent until the first dose of study medication.
- Treatment-emergent adverse events (TEAE): adverse events that occur or worsen from the start of the first dose of study medication to +56 days after the last dose of study medication.
- Post-treatment adverse events: adverse events that occurred or worsened after the last dose of study treatment + 56 days until the end of the trial.

If the AE start date is completely/partially missing, pre-medication/medication period AE will be flagged after imputation according to the rules in Section 5.3.

All adverse events will be coded using MedDRA (version 25.0 or higher).

Adverse events will be summarized by actual treatment group in count data. Include the following information: All adverse events, pretreatment adverse events, treatment-emergent adverse events (TEAE), serious TEAE, TEAE leading to study drug discontinuation, TEAE leading to study drug dose reduction, TEAE leading to study drug interruption, TEAE leading to death, severe TEAE, severe TEAE leading to study drug discontinuation, severe TEAE leading to study drug dose reduction, severe TEAE leading to study drug interruption, TEAE related to study drug (TRAE), serious TRAE, severe TRAE, TRAE leading to study drug discontinuation, TRAE leading to study drug dose reduction, TRAE leading to study drug interruption, TRAE leading to death, TEAE of special interest, and post-treatment adverse events.

Hypoglycemic events will be summarized by actual treatment group as count data. This included information on grade 1 (plasma glucose ≤ 3. 9 mmol/L), grade 2 (plasma glucose < 3.0 mmol/L), grade 3 (severe hypoglycemia), relative hypoglycemia, possible symptomatic hypoglycemic events, and the presence of other precipitating factors for hypoglycemia.

Adverse events will be summarized by MedDRA coded SOC and PT for the following categories:

- TEAE
- Serious TEAE
- TEAE Leading to Study Drug Discontinuation
- TEAE leading to dose reduction of study drug
- TEAE leading to study drug interruption

- TEAE leading to death
- TRAE
- Serious TRAE
- TRAE leading to study drug discontinuation
- TRAE leading to dose reduction of study drug
- TRAE leading to study drug interruption
- TRAE leading to death

Adverse events will be summarized by MedDRA coded SOC, PT and severity for the following categories:

- TEAE by maximum severity
- Serious TEAE by maximum severity
- TEAE Leading to Study Drug Discontinuation by Maximum Severity
- TEAE Leading to Study Drug Dose Reduction by Maximum Severity
- TEAE Leading to Study Drug Interruption by Maximum Severity
- TEAE Leading to Death by Maximum Severity
- TRAE by maximum severity
- Serious TRAE by maximum severity
- TRAE Leading to Study Drug Discontinuation by Maximum Severity
- TRAE Leading to Study Drug Dose Reduction by Maximum Severity
- TRAE Leading to Study Drug Interruption by Maximum Severity
- TRAE Leading to Death by Maximum Severity
- TEAE of Special Interest
- TEAE of Special Interest by Maximum Severity
- TEAE of Hypoglycemic Events
- Hypoglycemic events TEAE by maximum severity

The summaries of the above AE categories are sorted by the overall incidence of AE in the corresponding category from high to low.

Subject listings of all TEAE, treatment-emergent adverse events, TEAE leading to permanent discontinuation of study drug, serious TEAE, and deaths (TEAE start date, end date, severity, relationship to drug, action taken, outcome) will be provided.

6.6.2 Laboratory Tests

Laboratory tests include hematology, blood biochemistry, blood lipids, coagulation function indicators, urinalysis, myocardial enzymes, virus serology, thyroid function indicators and other indicators. See Table 1 for specific laboratory parameters and classification.

Table 1. Routine Laboratory Test Results

Category	Indicators
Blood routine	Red blood cell count (RBC), hemoglobin (HGB), hematocrit (HCT), white blood cell count (WBC), platelet count (PLT), differential white blood cell count (lymphocytes (LYM), neutrophils (ANC), monocytes (MONO), eosinophils (EOS), basophils (BASO), lymphocyte proportion (LYMLE), neutrophil proportion (NEUTLE), monocyte proportion (MONOLE), eosinophil proportion (EOSLE), basophil proportion (BASOLE))
Blood biochemistry	Liver function (serum total bilirubin (TBIL), direct bilirubin (DBIL), alanine aminotransferase (ALT), aspartate aminotransferase (AST), aminoacyl transpeptidase (GGT), alkaline phosphatase (ALP), albumin (ALB), total protein (TP), lactate dehydrogenase (LDH)), renal function (urea/urea nitrogen (BUN), uric acid (UA), creatinine (Cr), blood electrolytes (sodium (Na), potassium (K), chloride (Cl), calcium (Ca))
Blood lipids	Total cholesterol (TC), triglycerides (TG), high-density lipoprotein cholesterol (HDL-C), low-density lipoprotein cholesterol (LDL-C)
Coagulation function indicators	Prothrombin time (PT), activated partial thromboplastin time (APTT), international normalized ratio (INR)
Urinalysis	Urine pH, urine protein, urine glucose, urine red blood cells, urine white blood cells
Myocardial enzyme spectrum	Creatine kinase (CK), creatine kinase isoenzyme (CK-MB), troponin I (cTnI) or troponin T (cTnT), myoglobin
Other	Blood amylase, blood lipase, calcitonin
Viral serology	Hepatitis B surface antigen (HBsAg), hepatitis B surface antibody (HBsAb), hepatitis B core antibody (HBcAb), hepatitis B E antibody (HBeAb), hepatitis B E antigen (HBeAg), hepatitis C virus (HCV) antibody, human immunodeficiency virus (HIV) antibody, syphilis antibody
Thyroid function indicators	Thyroid stimulating hormone (TSH), free triiodothyronine (FT3), free thyroxine (FT4)

Measured values and changes from baseline of hematology, blood chemistry (except urea and urea nitrogen), lipids, coagulation parameters, myocardial enzymes (except cTnI), thyroid function parameters and other parameters will be summarized descriptively by actual treatment group by visit.

Shift tables showing whether hematology, blood biochemistry, blood lipids, coagulation function indicators, myocardial enzymes, thyroid function indicators, urinalysis and other indicators before and

after treatment will be normal and abnormal with clinical significance will be summarized descriptively by actual treatment group.

A listing of all laboratory test results will be provided.

6.6.3 Vital Signs

Vital signs include temperature, pulse, respiratory rate, and blood pressure.

Measured values and changes from baseline of vital signs will be summarized by actual treatment group in the form of measured data at each visit.

A listing of vital signs will be provided.

6.6.4 Electrocardiogram

Electrocardiogram parameters included PR interval, heart rate, RR interval, QT interval, and QTcF (calculation formulas are provided in Appendix 4 of the clinical study protocol).

The measured values and changes from baseline of each indicator of ECG will be summarized by actual treatment group in the form of measurement data by visit.

Shift tables of normal and abnormal clinically significant ECG and echocardiogram values before and after treatment will be summarized by actual treatment group in the form of count data.

6.6.5 Physical examination

Physical examinations included general, respiratory, cardiovascular, abdomen, skin, head and neck (including ears, eyes, nose, and throat), lymph nodes, thyroid, musculoskeletal (including spine and extremities), genital/anal, and neurological assessments.

Physical examination findings will be summarized by visit as counts by actual treatment group.

A listing of physical examinations will be provided.

6.6.6 C-SSRS Questionnaire

Data collected on the C-SSRS questionnaire in the eCRFs will be summarized descriptively by visit in the form of count data or measurement data for each actual treatment group. Includes 1-2 questions for the suicidal ideation section, all questions for suicidal behavior.

6.6.7 PHQ-9 questionnaire

The PHQ-9 questionnaire total score, change from baseline in total score, and percent change from baseline in total score will be summarized descriptively by visit as measured data for the actual treatment group in the eCRF.

Each subject can be classified as minimally depressed, mildly depressed, moderately depressed, moderately to severely depressed, and severely depressed based on the total score collected on the PHQ-9 questionnaire in the eCRF. The rules for dividing depression by total score are as follows:

- Minimal depression: total score 1-4

- Mild depression: total score 5-9 points
- Moderate depression: total score of 10-14 points
- Moderate to severe depression: total score 15-19 points
- Major depression: total score 20-27 points

Depression will be summarized by visit as count data for each actual treatment group.

6.7 Quality Of Life Analysis

Quality of life analyses will be performed in the modified intent-to-treat analysis set.

The secondary efficacy endpoint will be to evaluate the improvement in quality of life (IWQoL-Lite questionnaire) after 24 weeks of dosing. The answers to the 20 questions included in the IWQoL-Lite questionnaire in the eCRF will be assigned according to the following rules, and the total score of the IWQoL-Lite questionnaire will be calculated for each subject:

- Never = 1, rarely = 2, sometimes = 3, often = 4, and always = 5.
- Totally incorrect = 1, some correct = 2, moderately correct = 3, mostly correct = 4, and completely correct = 5.

The sum of the scores of 1-5, 16, and 17 items of the IWQoL-Lite questionnaire in the eCRF will be defined as the physical composite score, the sum of the scores of 1-3, 16, and 17 items will be defined as the physical function composite score, and the sum of the scores of 6-8, 9-15, and 18-20 items will be defined as the psychological composite score.

Measurements, changes from baseline, and percent changes from baseline for the following IWQoL-Lite questionnaire-related variables will be summarized descriptively by visit in the form of measurement data for each planned treatment group. The mean values, changes from baseline, and percent changes from baseline over time will be also plotted by visit by planned treatment group.

- Total score
- Physical Composite Score
- Physical function composite score
- Psychological composite score

6.8 Immunogenicity Analysis

Immunogenicity analyses will be performed in the Safety Analysis Set.

The positive rates of anti-IBI362 antibodies (ADA) and neutralizing antibodies (NAb) will be summarized by actual treatment group.

6.9 Pharmacokinetic Analysis

Detailed PK analysis will be provided in a separate PK analysis report.

6.10 Pharmacodynamic Analysis

The pharmacodynamic analysis will be performed in the pharmacodynamic analysis set.

Pharmacodynamic parameters included fasting insulin.

Pharmacodynamic parameters will be summarized descriptively by visit for each planned treatment group, and mean values, changes from baseline, and percent changes from baseline will be plotted by visit for each planned treatment group over time.

6.11 Interim Analysis And Data Monitoring Meetings

To support the sponsor's communication with regulatory authorities regarding the IBI362 development strategy and Phase 3 study design, at least one interim analysis of efficacy and safety data will be performed in this trial prior to the primary endpoint visit. The results of the interim analysis will not be disclosed to the personnel involved in the operation of this study, and will not affect the operation of the trial and the subsequent study progress. The results of the interim analysis will only be used to support the dose recommendation for Phase 3 trial, and the clinical study design will not be changed due to the results of the interim analysis.

The interim analysis will be completed by an unblinded sponsor team not directly involved in the operation of the trial, and the sponsor unblinded team will be identified prior to the start of the first interim analysis. The unblinded team will consist of a medical monitor, a statistician, and a programmer. Before the first interim analysis node, the blinded project statistician and programmer of this trial will develop the statistical analysis plan, prepare the interim analysis procedure based on the blinded data, and submit the procedure and the statistical analysis plan to the statistician of the unblinded team. At the node of interim analysis, the unblinded drug administrator will transmit the blind code information of the randomized subjects before the analysis node to the statistician of the unblinded team, and the statistician of the unblinded team will generate the interim analysis report according to the blind code and the pre-prepared procedure. Additional analyses of safety and/or efficacy will be performed if necessary. The unblinded team will submit the efficacy and safety results summarized at the treatment group level to the designated unblinded personnel of the sponsor for the design of the phase 3 trial and communication with regulatory authorities.

Except for the sponsor's unblinded team and designated sponsor's unblinded personnel, the sponsor's members not in direct contact with subjects and investigators will be unblinded after all subjects have completed the Week 25 primary endpoint visit and the primary endpoint database lock, and the remaining sponsor's personnel will remain blinded during this phase of the study until the database is finally locked after all subjects have completed the Week 36 discontinuation visit.

There will be no planned data monitoring meetings for this trial.

7 References

[1]. China Food and Drug Administration. Good Clinical Practice (GCP). 2013.

[2]. China Food and Drug Administration. Guidelines for the Structure and Content of Clinical Study Reports for Chemical Drugs. March 2005.

[3]. China Food and Drug Administration. Biostatistical Guidelines for Drug Clinical Trials (Draft for Comments). June 3, 2016.

[4]. ICH. ICH E9 Guideline: Statistical Principles for Clinical Trials, 1998. Available at https://www.ich.org/fileadmin/Public _Web_Site/ICH _Products/Guidelines/Efficiacy/E9/Step4/E9_Guideline. Pdf

[5]. ICH. ICH E3 Guideline: Structure and Content of Clinical Study Reports Questions & Answers, 2012. Available at http://www.ich.org/fileadmin/Public _Web_Site/ICH _Products/Guidelines/Efficiacy/E3/E3_QAs_R1_Step4. Pdf

[6]. Patel JS, Oh Y, Rand KL, Wu W, Cyders MA, Kroenke K, Stewart JC. Measurement variability of the patient health questionnaire-9 (PHQ-9) depression screen in U.S. Adults across sex, race/ethnicity, and education level: NHANES 2005-2016. Depress Anxiety.2019 Sep; 36 (9): 813-823. Doi: 10.1002/da.22940. Epub 2019 Jul 29. PMID: 31356710; PMCID: PMC6736700.
