# Supplementary material for: A phase 2 randomised controlled trial of mazdutide in Chinese overweight adults or adults with obesity
Source: Nat Commun. 2023 Dec 14;14:8289. doi: 10.1038/s41467-023-44067-4 (PMC10719339; doi:10.1038/s41467-023-44067-4)
Supplement: Supplementary file 3 — Reporting Summary [file 41467_2023_44067_MOESM3_ESM.pdf]

nature portfolio

Corresponding author(s): Linong Ji, Lei Qian

Last updated by author(s): Nov 28, 2023

Reporting Summary

Nature Portfolio wishes to improve the reproducibility of the work that we publish. This form provides structure for consistency and transparency in reporting. For further information on Nature Portfolio policies, see our Editorial Policies and the Editorial Policy Checklist.

Statistics

For all statistical analyses, confirm that the following items are present in the figure legend, table legend, main text, or Methods section.

n/a	Confirmed	
☐	☒	The exact sample size (*n*) for each experimental group/condition, given as a discrete number and unit of measurement
☐	☒	A statement on whether measurements were taken from distinct samples or whether the same sample was measured repeatedly
☐	☒	The statistical test(s) used AND whether they are one- or two-sided<br>*Only common tests should be described solely by name; describe more complex techniques in the Methods section.*
☐	☒	A description of all covariates tested
☐	☒	A description of any assumptions or corrections, such as tests of normality and adjustment for multiple comparisons
☐	☒	A full description of the statistical parameters including central tendency (e.g. means) or other basic estimates (e.g. regression coefficient) AND variation (e.g. standard deviation) or associated estimates of uncertainty (e.g. confidence intervals)
☐	☒	For null hypothesis testing, the test statistic (e.g. *F*, *t*, *r*) with confidence intervals, effect sizes, degrees of freedom and *P* value noted<br>*Give P values as exact values whenever suitable.*
☒	☐	For Bayesian analysis, information on the choice of priors and Markov chain Monte Carlo settings
☐	☒	For hierarchical and complex designs, identification of the appropriate level for tests and full reporting of outcomes
☒	☐	Estimates of effect sizes (e.g. Cohen's *d*, Pearson's *r*), indicating how they were calculated

Our web collection on statistics for biologists contains articles on many of the points above.

Software and code

Policy information about availability of computer code

Data collection	Data were collected using TrialMaster Electronic Data Capture 5.0.
Data analysis	All statistical analyses were done using SAS version 9·4.

For manuscripts utilizing custom algorithms or software that are central to the research but not yet described in published literature, software must be made available to editors and reviewers. We strongly encourage code deposition in a community repository (e.g. GitHub). See the Nature Portfolio guidelines for submitting code & software for further information.

Data

Policy information about availability of data

All manuscripts must include a data availability statement. This statement should provide the following information, where applicable:

- Accession codes, unique identifiers, or web links for publicly available datasets
- A description of any restrictions on data availability
- For clinical datasets or third party data, please ensure that the statement adheres to our policy

Data underlying the analyses in this study cannot be publicly available due to the sponsor's (Innovent Biologics) contractual obligations and data privacy laws. Innovent Biologics will provide individual de-identified participant data underlying the results reported in this article. Data are available to request 6 months after the acceptance of this article. No expiration of data requests is currently set. Requests should be made to the corresponding authors (L.J. [jiln@bjmu.edu.cn] or L.Q. [cnradium@126.com]) and will be evaluated within 3 months. Access is provided after proposed use of the data has been approved by a review committee and receipt of a signed data access agreement with Innovent Biologics. The clinical study protocol and statistical analysis plan are provided as Supplementary Note 1 and

Supplementary Note 2, respectively. Source data are provided with this paper.

Research involving human participants, their data, or biological material

Policy information about studies with human participants or human data. See also policy information about sex, gender (identity/presentation), and sexual orientation and race, ethnicity and racism.

Reporting on sex and gender	Self-reporting sex was collected as a baseline characteristics. Of 248 participants enrolled, 48% were male and 52% were female.<br>In this phase 2 study, sample size within each treatment group was limited and sex-based subgroup analysis was not pre-specified in the protocol and not conducted therefore.<br>In addition, male participants generally had a greater body weight at baseline than females. Baseline body weight was used as a covariate in both primary and sensitivity analyses of the primary endpoint.
Reporting on race, ethnicity, or other socially relevant groupings	This study was conducted in China and all participants were Asian. No other socially relevant groupings were collected.
Population characteristics	This study enrolled Chinese adults (aged 18–75 years, both inclusive) with overweight (body-mass index [BMI] ≥24 kg/m2) accompanied by hyperphagia and/or at least one obesity-related comorbidity (pre-diabetes, hypertension, dyslipidaemia, fatty liver, weight-bearing arthralgia, dyspnoea or obstructive sleep apnoea syndrome) or with obesity (BMI ≥28 kg/m2), and without diabetes.<br>The enrolled participants had a mean age of 35·5 years, a mean baseline bodyweight of 89·4 kg and a mean baseline BMI of 31·8 kg/m2. A total of 208 participants had a BMI of 28 kg/m2 or greater at baseline.
Recruitment	Investigators from 20 hospitals in China screened and enrolled participants into this study. Participants must meet the all the inclusion criteria and should not meet any one of the exclusion criteria as defined in the clinical study protocol. All participants provided written informed consent before study entry. This was a randomized, double-blind study where the participants, investigators, study site personnel involved in treating ans assessing participants ans sponsor personnel were masked to the study treatment allocation. Possibility for potential self-selection bias or other biases were minimized with the strict adherence to the inclusion and exclusion criteria, and randomization and masking procedures as defined in the protocol.
Ethics oversight	The study protocol and informed consent form were approved by ethics committees of all study sites: Peking University People's Hospital; The First Affiliated Hospital and Clinical Medicine College of Henan University of Science and Technology; The Fourth Affiliated Hospital of Harbin Medical University; Huzhou Central Hospital; Shandong Province Qianfoshan Hospital; Pingxiang People' s Hospital; The First Affiliated Hospital of Bengbu Medical College; Jinan Central Hospital; The Second Hospital of Hebei Medical University; Chu Hsien-I Memorial Hospital; Jiangsu Province Hospital; Sun Yat-Sen Memorial Hospital; Shanghai Tenth People's Hospital of Tong Ji University; Qilu Hospital of Shandong University; Inner Mongolia Autonomous Region People's Hospital; Luoyang Central Hospital; Tonghua Central Hospital; Jingzhou Central Hospital; The First Affiliated Hospital of Nanyang Medical College.

Note that full information on the approval of the study protocol must also be provided in the manuscript.

Field-specific reporting

Please select the one below that is the best fit for your research. If you are not sure, read the appropriate sections before making your selection.

☒ Life sciences ☐ Behavioural & social sciences ☐ Ecological, evolutionary & environmental sciences

For a reference copy of the document with all sections, see nature.com/documents/nr-reporting-summary-flat.pdf

Life sciences study design

All studies must disclose on these points even when the disclosure is negative.

Sample size	The sample size of 240 participants was estimated to provide at least 95% power to show the superiority of optimal mazdutide dose to placebo in terms of bodyweight change from baseline to week 24, at a two-sided significance level of 0·05. In the calculation of the sample size, it was assumed the mean percentage change in bodyweight from baseline to week 24 of 2·3% with placebo and −10% with optimal mazdutide dose, a common standard deviation of approximately 8%, and a dropout rate of 20%.
Data exclusions	No data was excluded from the analysis.
Replication	The study has three cohorts, with 62 receiving mazdutide 3 mg, 63 receiving mazdutide 4·5 mg, 61 receiving mazdutide 6 mg and 62 receiving placebo. This allowed replication and reproducibility of findings across different treatment groups.
Randomization	A block randomisation method with a block size of 24 was used to randomly assign eligible participants 3:1:3:1:3:1 to receive mazdutide 3 mg, 4·5 mg, 6 mg or matching placebo using an interactive web response system, stratified by BMI at screening (<28 kg/m2 vs ≥28 kg/m2). Randomisation list was generated by an independent statistician who was not involved in the clinical operations of the study.
Blinding	The study drugs and placebo were identically labelled and indistinguishable in appearance. The participants, investigators, study site personnel involved in treating and assessing participants and sponsor personnel in contact with the investigators and participants were masked to treatment allocation until the database lock of the first part, while other sponsor personnel remained blinded until all participants

in the first part had finished week 24.

Reporting for specific materials, systems and methods

We require information from authors about some types of materials, experimental systems and methods used in many studies. Here, indicate whether each material, system or method listed is relevant to your study. If you are not sure if a list item applies to your research, read the appropriate section before selecting a response.

Materials & experimental systems

n/a	Involved in the study	
x		Antibodies
x		Eukaryotic cell lines
x		Palaeontology and archaeology
x		Animals and other organisms
	x	Clinical data
	x	Dual use research of concern
x		Plants

Methods

n/a	Involved in the study	
x		ChIP-seq
x		Flow cytometry
x		MRI-based neuroimaging

Clinical data

Policy information about clinical studies
All manuscripts should comply with the ICMJEguidelines for publication of clinical research and a completedCONSORT checklist must be included with all submissions.

Clinical trial registration: This study is registered with ClinicalTrials.gov, number NCT04904913.

Study protocol: The full clinical study protocol was supplemented as the supplementary information file.

Data collection: Between June 11, 2021 and Oct. 1, 2021, patients were enrolled at and data were collected from the following study centers: Peking University People's Hospital, The First Affiliated Hospital and Clinical Medicine College of Henan University of Science and Technology, The Fourth Affiliated Hospital of Harbin Medical University, Huzhou Central Hospital, Shandong Province Qianfoshan Hospital, Pingxiang People' s Hospital, The First Affiliated Hospital of Bengbu Medical College, Jinan Central Hospital, The Second Hospital of Hebei Medical University, Chu Hsien-I Memorial Hospital, Jiangsu Province Hospital, Sun Yat-Sen Memorial Hospital, Shanghai Tenth People's Hospital of Tong Ji University, Qilu Hospital of Shandong University, Inner Mongolia Autonomous Region People's Hospital, Luoyang Central Hospital, Tonghua Central Hospital, Jingzhou Central Hospital, The First Affiliated Hospital of Nanyang Medical College.

Outcomes: The primary endpoint was percentage change from baseline to week 24 in bodyweight. Secondary efficacy endpoints included proportion of participants achieving bodyweight loss of 5% or more and 10% or more from baseline to week 24, change from baseline to week 24 in bodyweight, waist circumference and BMI, change from baseline to week 24 in glucose metabolism parameters (HbA1c, fasting plasma glucose and fasting insulin), changes from baseline to week 24 in cardiovascular risk factors (blood pressure, lipids [total cholesterol, low density lipoprotein (LDL) cholesterol, high density lipoprotein (HDL) cholesterol and triglycerides]), changes from baseline to week 12 and week 24 in serum uric acid and alanine aminotransferase (ALT), changes in IWQOL-Lite scores, as well as changes from baseline to week 36 in bodyweight, waist circumference, BMI, glucose and cardiovascular factors.

Secondary safety endpoints included safety, tolerability and immunogenicity of mazdutide. Secondary safety endpoints also included C-SSRS and PHQ-9 assessments.

Bodyweight, vital signs, electrocardiogram and adverse events were monitored at every visit. Waist circumference was measured at screening, end of lead-in, day 1 and every 4 weeks thereafter through week 36. Local laboratory parameters were monitored at screening, day 1, every 4 weeks thereafter through week 24 and week 36. Central laboratory parameters (lipids and glycated haemoglobin A1c [HbA1c]) were monitored at day 1, week 12 and week 24 (Wuxi AppTec Inc., Shanghai). Pharmacodynamic (fasting plasma glucose and fasting insulin) and immunogenicity parameters were monitored at day 1 and weeks 2, 4, 8, 16, 24 and 32 (Wuxi AppTec Inc., Shanghai). Impact of Weight on Quality of Life–Lite (IWQOL-Lite) questionnaire assessment was conducted at baseline, week 12 and week 24. Patient Health Questionnaire-9 (PHQ-9) and the Columbia-Suicide Severity Rating Scale (C-SSRS) assessment were conducted at baseline and weeks 12, 24 and 36.
